# Supplementary material for: Survival and new-onset morbidity after critical care admission for acute pancreatitis in Scotland: a national electronic healthcare record linkage cohort study
Source: BMJ Open. 2018 Dec 14;8(12):e023853. doi: 10.1136/bmjopen-2018-023853 (PMC6303604; doi:10.1136/bmjopen-2018-023853)
Supplement: Supplementary file 1 [file bmjopen-2018-023853supp001.pdf]

Supplementary data.

Title: Survival and new-onset morbidity after critical care admission for acute pancreatitis: a national electronic healthcare record linkage study.

Authors: Chiara Ventre, Sian Nowell, Cat Graham, Doug Kidd, Christos Skouras and Damian J. Mole

Supplementary material includes:

1 table

11 supplementary figures

On 13 pages

Supplementary Table 1. Categorisation of Causes of Death. The ICD-10 categories for cause of death were allocated to six groups as shown.

**Category of death**	**ICD 10 Code**
Circulatory/Cardiovascular system	IX Diseases of the circulatory system
Digestive/Metabolic System	III Diseases of the blood and blood-forming organs and certain disorders involving the immune mechanism<br>*D50-D53 Nutritional anaemias*
	IV Endocrine, nutritional and metabolic diseases
	XI Diseases of the digestive system
	XVIII Symptoms, signs and abnormal clinical and laboratory findings, not elsewhere classified<br>*R10-R19 Symptoms and Signs involving the digestive system and abdomen*
Neoplasms	II Neoplasms
Respiratory System	X Diseases of the Respiratory System
Other	I Certain infectious and parasitic diseases
	III Diseases of the blood and blood-forming organs and certain disorders involving the immune mechanism<br>*Excluding D50-D53*
	V Mental and behavioural disorders
	VI Diseases of the nervous system
	VII Diseases of the eye and adnexa
	VIII Diseases of the ear and mastoid process
	XII Diseases of the skin and subcutaneous tissue
	XIII Diseases of the musculoskeletal system and connective tissue
	XIV Diseases of the genitourinary system
	XV Pregnancy, childbirth and the puerperium
	XVI Certain conditions originating in the perinatal period
	XVII Congenital malformations, deformations and chromosomal abnormalities
	XVIII Symptoms, signs and abnormal clinical and laboratory findings, not elsewhere classified<br>*Excluding R10-R19*
	XIX Injury, poisoning and certain other consequences of external causes
	XX External causes of morbidity and mortality
	XXI Factors influencing health status and contact with health services
	XXII Codes for special purposes

A

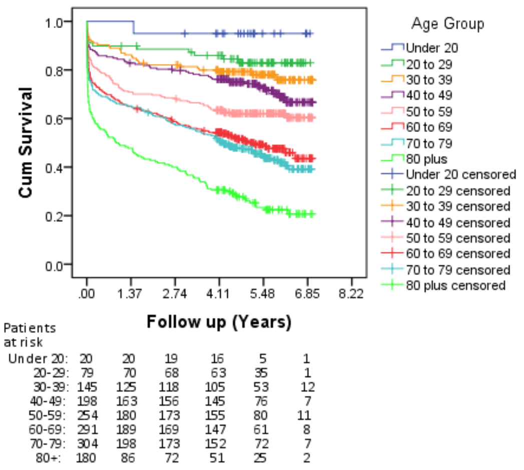

B

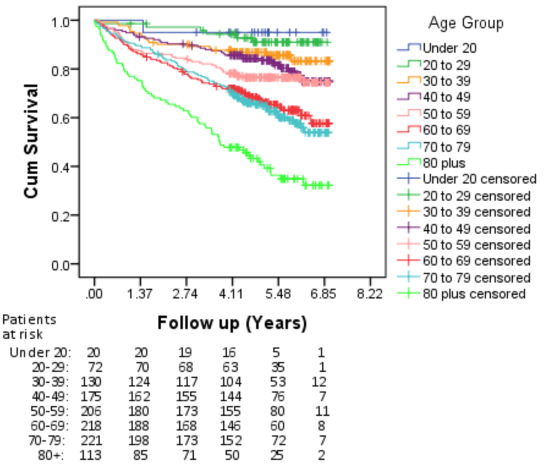

Pairwise Comparisons – including in hospital deaths

	Age group	Under 20		20 to 29		30 to 39		40 to 49		50 to 59		60 to 69		70 to 79		80 plus	
		Chi-Square	Sig.	Chi-Square	Sig.	Chi-Square	Sig.	Chi-Square	Sig.	Chi-Square	Sig.	Chi-Square	Sig.	Chi-Square	Sig.	Chi-Square	Sig.
Log Rank (Mantel-Cox)	Under 20			1.614	.204	2.907	.088	4.199	.040	7.380	.007	11.599	.001	13.092	.000	24.091	.000
	20 to 29	1.614	.204			.954	.329	3.962	.047	11.683	.001	25.914	.000	32.015	.000	66.499	.000
	30 to 39	2.907	.088	.954	.329			1.486	.223	10.898	.001	31.203	.000	40.173	.000	92.223	.000
	40 to 49	4.199	.040	3.962	.047	1.486	.223			5.256	.022	25.194	.000	35.378	.000	92.343	.000
	50 to 59	7.380	.007	11.683	.001	10.898	.001	5.256	.022			8.366	.004	15.589	.000	63.022	.000
	60 to 69	11.599	.001	25.914	.000	31.203	.000	25.194	.000	8.366	.004			1.094	.296	30.182	.000
	70 to 79	13.092	.000	32.015	.000	40.173	.000	35.378	.000	15.589	.000	1.094	.296			21.152	.000
	80 plus	24.091	.000	66.499	.000	92.223	.000	92.343	.000	63.022	.000	30.182	.000	21.152	.000		

Pairwise Comparisons

	Age group	Under 20		20 to 29		30 to 39		40 to 49		50 to 59		60 to 69		70 to 79		80 plus	
		Chi-Square	Sig.	Chi-Square	Sig.	Chi-Square	Sig.	Chi-Square	Sig.	Chi-Square	Sig.	Chi-Square	Sig.	Chi-Square	Sig.	Chi-Square	Sig.
Log Rank (Mantel-Cox)	Under 20			.195	.659	1.212	.271	1.981	.159	3.281	.070	5.997	.014	6.718	.010	15.130	.000
	20 to 29	.195	.659			1.730	.188	4.552	.033	7.764	.005	17.852	.000	21.251	.000	49.740	.000
	30 to 39	1.212	.271	1.730	.188			.986	.321	4.151	.042	16.184	.000	20.474	.000	59.844	.000
	40 to 49	1.981	.159	4.552	.033	.986	.321			1.272	.259	12.308	.000	17.081	.000	60.511	.000
	50 to 59	3.281	.070	7.764	.005	4.151	.042	1.272	.259			5.838	.016	9.458	.002	47.244	.000
	60 to 69	5.997	.014	17.852	.000	16.184	.000	12.308	.000	5.838	.016			.326	.568	22.791	.000
	70 to 79	6.718	.010	21.251	.000	20.474	.000	17.081	.000	9.458	.002	.326	.568			19.713	.000
	80 plus	15.130	.000	49.740	.000	59.844	.000	60.511	.000	47.244	.000	22.791	.000	19.713	.000		

Supplementary Figure 1. Survival by age. Kaplan-Meier plots of the proportion of surviving patients over time in years. Patients were categorised by age. Vertical dashes represent right-censored patients. The number of patients remaining at risk at each time point are presented under the chart. Log-rank pair-wise analyses are presented above the corresponding chart **a.** Analyses including in-hospital deaths. **b.** Analyses excluding in-hospital deaths

A

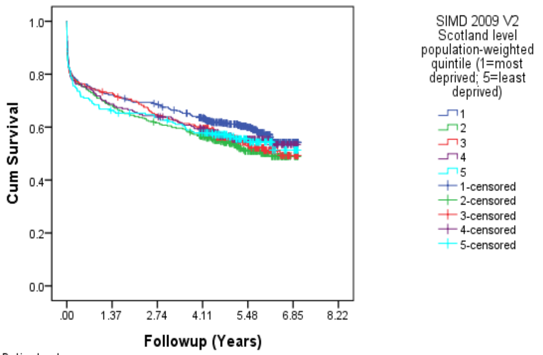

Patients at risk

1:	438	316	298	261	134	15
2:	344	235	211	184	93	8
3:	262	190	167	151	75	10
4:	227	156	145	125	57	6
5:	193	127	120	106	46	10

B

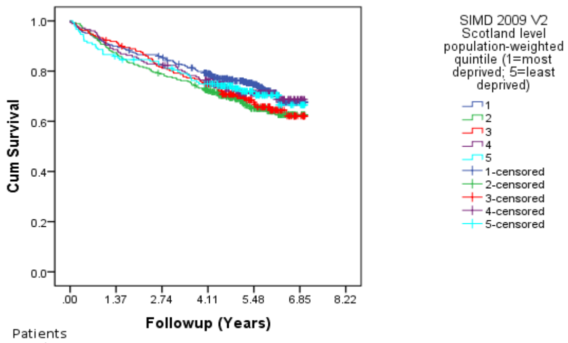

Patients at risk:

1:	349	314	296	259	133	15
2:	269	235	211	184	92	8
3:	208	190	167	151	75	10
4:	175	155	144	124	57	6
5:	149	127	120	106	45	10

Pairwise Comparisons – including in hospital deaths

	SIMD 2009 V2 Scotland level population-weighted quintile (1= most deprived; 5=least deprived)	1 Chi-Square	1 Sig.	2 Chi-Square	2 Sig.	3 Chi-Square	3 Sig.	4 Chi-Square	4 Sig.	5 Chi-Square	5 Sig.
Log Rank (Mantel-Cox)	1			3.634	.057	1.801	.180	.248	.618	.456	.499
	2	3.634	.057			.175	.676	1.207	.272	.695	.405
	3	1.801	.180	.175	.676			.447	.504	.229	.632
	4	.248	.618	1.207	.272	.447	.504			.030	.864
	5	.456	.499	.695	.405	.229	.632	.030	.864		

Pairwise Comparisons – excluding in hospital deaths

	SIMD 2009 V2 Scotland level population-weighted quintile (1 = most deprived; 5 = least deprived)	1 Chi-Square	1 Sig.	2 Chi-Square	2 Sig.	3 Chi-Square	3 Sig.	4 Chi-Square	4 Sig.	5 Chi-Square	5 Sig.
Log Rank (Mantel-Cox)	1			3.473	.062	1.339	.247	.602	.438	1.130	.288
	2	3.473	.062			.293	.588	.643	.423	.205	.650
	3	1.339	.247	.293	.588			.075	.784	.000	.993
	4	.602	.438	.643	.423	.075	.784			.075	.785
	5	1.130	.288	.205	.650	.000	.993	.075	.785		

Supplementary Figure 2. Survival by Scottish Index of Multiple Deprivation (SIMD) quintile. Kaplan-Meier plots of the proportion of surviving patients over time in years. Patients were categorised by SIMD quintile. Vertical dashes represent right-censored patients. The number of patients remaining at risk at each time point are presented under the chart. Log-rank pair-wise analyses are presented above the corresponding chart **a.** Analyses including in-hospital deaths. **b.** Analyses excluding in-hospital deaths

A

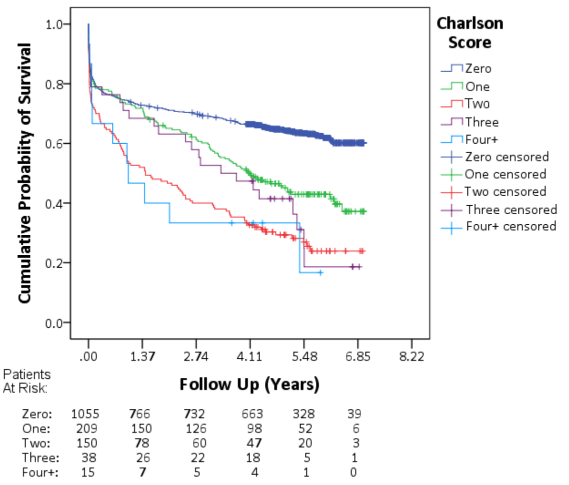

B

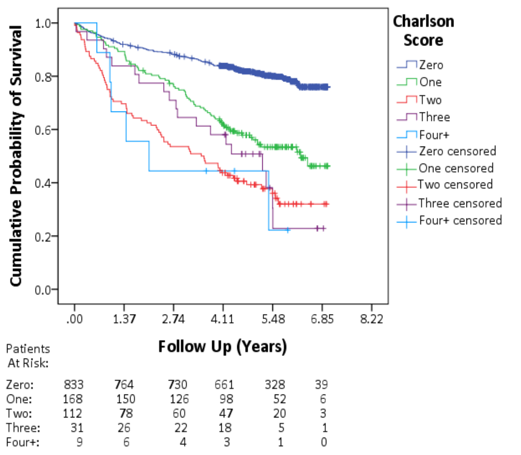

Pairwise Comparisons – including in-hospital deaths

	Charlson score	0		1		2		3		4+	
		Chi-Square	Sig.	Chi-Square	Sig.	Chi-Square	Sig.	Chi-Square	Sig.	Chi-Square	Sig.
Log Rank (Mantel-Cox)	0			22.485	.000	74.261	.000	12.801	.000	9.426	.002
	1	22.485	.000			13.189	.000	1.166	.280	3.167	.075
	2	74.261	.000	13.189	.000			1.147	.284	.038	.846
	3	12.801	.000	1.166	.280	1.147	.284			.782	.376
	4+	9.426	.002	3.167	.075	.038	.846	.782	.376		

Pairwise Comparisons – excluding in hospital deaths

		0		1		2		3		4+	
	Charlson score	Chi-Square	Sig.	Chi-Square	Sig.	Chi-Square	Sig.	Chi-Square	Sig.	Chi-Square	Sig.
Log Rank (Mantel-Cox)	0			52.398	.000	135.915	.000	35.007	.000	19.238	.000
	1	52.398	.000			12.621	.000	2.097	.148	3.383	.066
	2	135.915	.000	12.621	.000			.495	.482	.067	.796
	3	35.007	.000	2.097	.148	.495	.482			.441	.507
	4+	19.238	.000	3.383	.066	.067	.796	.441	.507		

Supplementary Figure 3. Survival by Charlson Score. Kaplan-Meier plots of the proportion of surviving patients over time in years. Patients were categorised by Charlson score. Vertical dashes represent right-censored patients. The number of patients remaining at risk at each time point are presented under the chart. Log-rank pair-wise analyses are presented above the corresponding chart **a.** Analyses including in-hospital deaths. **b.** Analyses excluding in-hospital deaths

A

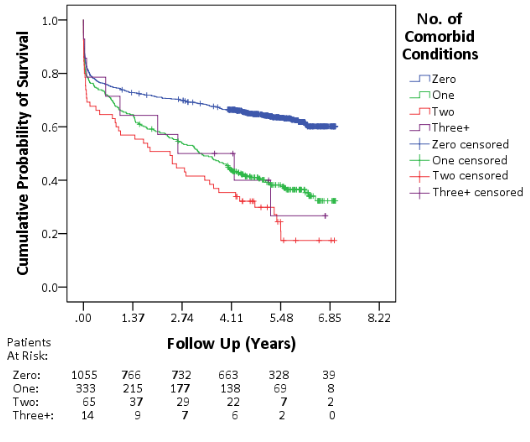

B

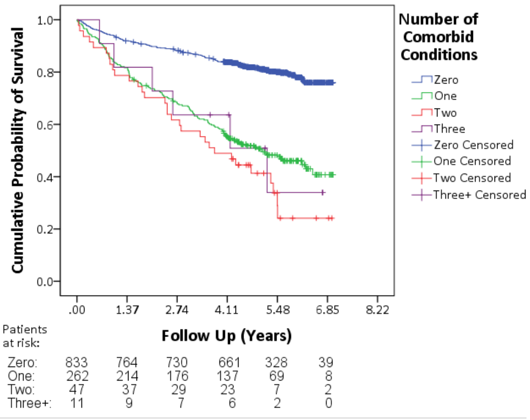

Pairwise Comparisons – including in hospital deaths									
		0		1		2		3+	
	Charlson No. of Conditios	Chi-Square	Sig.	Chi-Square	Sig.	Chi-Square	Sig.	Chi-Square	Sig.
Log Rank (Mantel-Cox)	0			57.146	.000	40.233	.000	3.749	.053
	1	57.146	.000			4.247	.039	.002	.968
	2	40.233	.000	4.247	.039			.607	.436
	3+	3.749	.053	.002	.968	.607	.436		

Pairwise Comparisons – excluding in hospital deaths									
	Charlson No. of Conditions	.00		1		2		3+	
		Chi-Square	Sig.	Chi-Square	Sig.	Chi-Square	Sig.	Chi-Square	Sig.
Log Rank (Mantel-Cox)	.00			113.399	.000	69.509	.000	9.289	.002
	1	113.399	.000			2.654	.103	.003	.954
	2	69.509	.000	2.654	.103			.324	.569
	3+	9.289	.002	.003	.954	.324	.569		

Supplementary Figure 4. Survival by number of conditions contributing to the Charlson Score. Kaplan-Meier plots of the proportion of surviving patients over time in years. Patients were categorised by the number of conditions contributing to their Charlson score. Vertical dashes represent right-censored patients. The number of patients remaining at risk at each time point are presented under the chart. Log-rank pair-wise analyses are presented above the corresponding chart **a.** Analyses including in-hospital deaths. **b.** Analyses excluding in-hospital deaths

A B

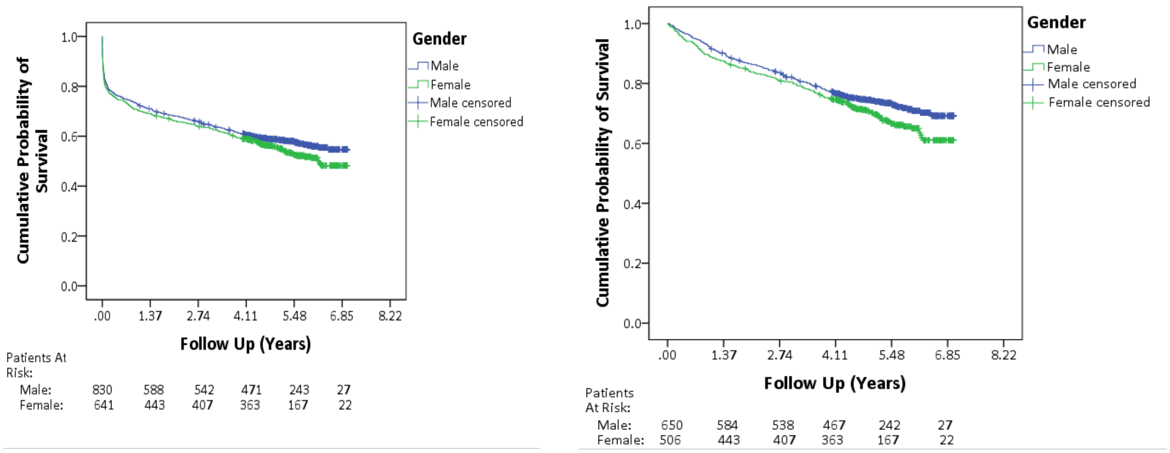

Supplementary Figure 5. Gender. Kaplan-Meier plots of the proportion of surviving patients over time in years. Patients were categorised by gender. Vertical dashes represent right-censored patients. The number of patients remaining at risk at each time point are presented under the chart. Log-rank analyses are presented to the right of the corresponding chart **a.** Analyses including in-hospital deaths (Log Rank test, P = 0.134). **b.** Analyses excluding in-hospital deaths (Log Rank test, P = 0.049)

A

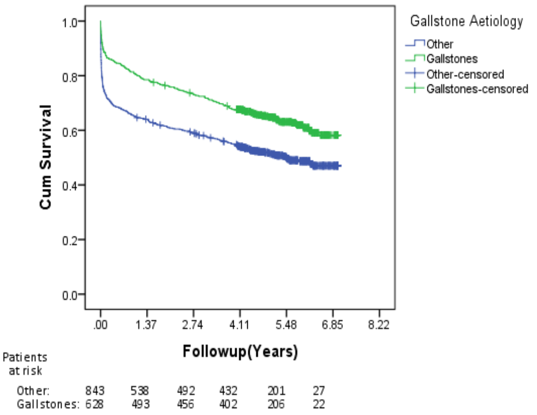

B

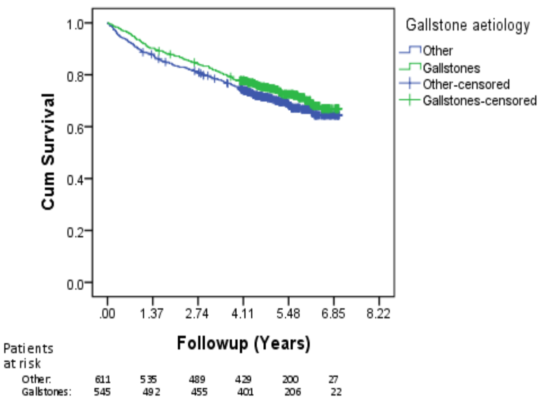

Supplementary Figure 6. Survival by aetiology of pancreatitis. Kaplan-Meier plots of the proportion of surviving patients over time in years. Patients were categorised by aetiology – gallstones or other causes. Vertical dashes represent right-censored patients. The number of patients remaining at risk at each time point are presented under the chart. Log-rank analyses are presented to the right of the corresponding chart **a.** Analyses including in-hospital deaths. (Log Rank test, Gallstones vs Other, P<0.001) **b.** Analyses excluding in-hospital deaths (Log Rank test, Gallstones vs Other, P=0.139)

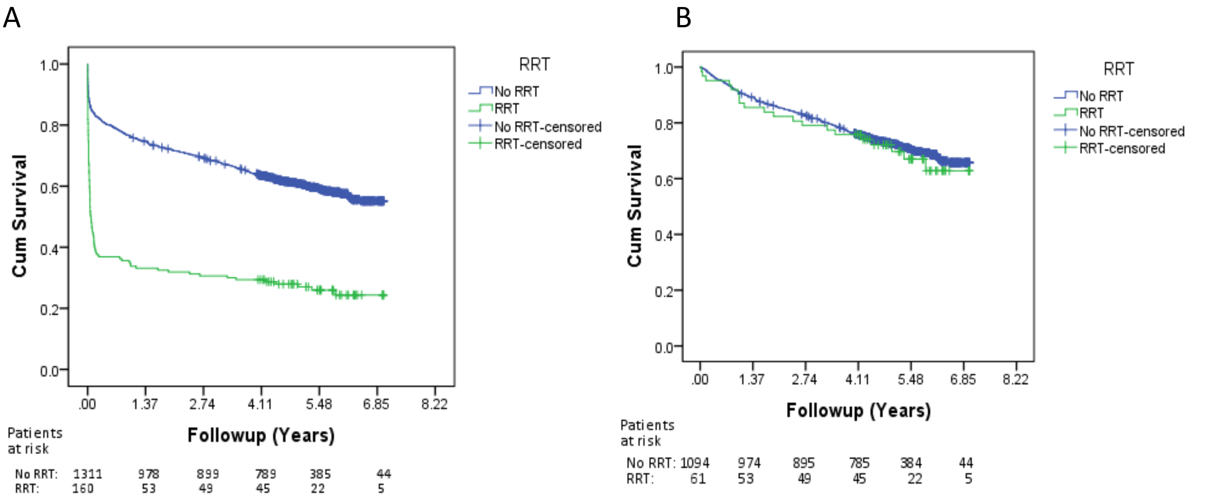

Supplementary Figure 7. Survival comparing use of renal replacement therapy (RRT). Kaplan-Meier plots of the proportion of surviving patients over time in years. Patients were categorised by whether they required RRT during the index admission. Vertical dashes represent right-censored patients. The number of patients remaining at risk at each time point are presented under the chart. Log-rank analyses are presented to the right of the corresponding chart **a.** Analyses including in-hospital deaths (Log Rank test RRT vs no RRT, P <0.001). **b.** Analyses excluding in-hospital deaths (Log Rank test RRT vs no RRT, P = 0.634).

A

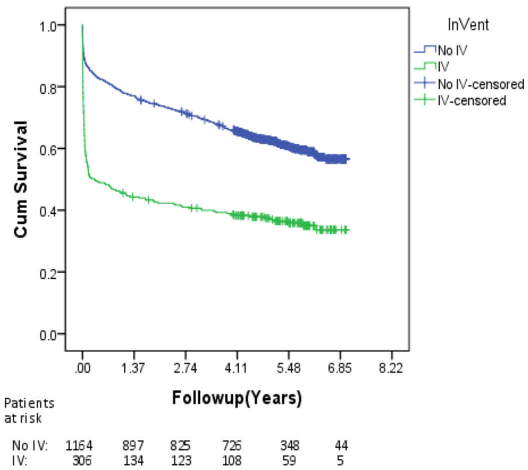

B

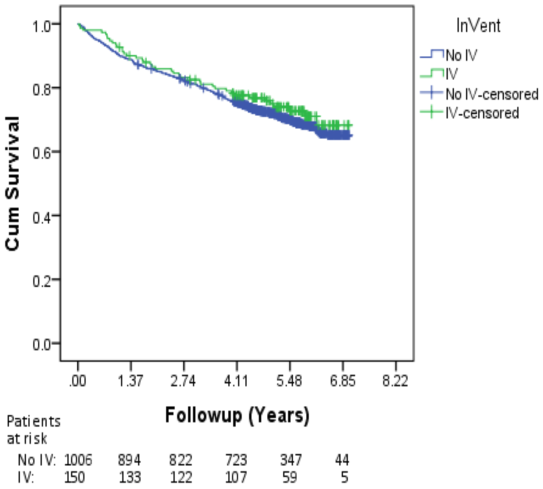

Supplementary Figure 8. Survival according to use of invasive ventilation. Kaplan-Meier plots of the proportion of surviving patients over time in years. Patients were categorised by whether they required invasive ventilation during the index admission. Vertical dashes represent right-censored patients. The number of patients remaining at risk at each time point are presented under the chart. Log-rank analyses are presented to the right of the corresponding chart **a.** Analysis including in-hospital deaths (Log Rank test inv. vent vs no inv. vent, P < 0.001). **b.** Analysis excluding in-hospital deaths (Log Rank test inv. vent vs no inv. vent, P = 0.428).

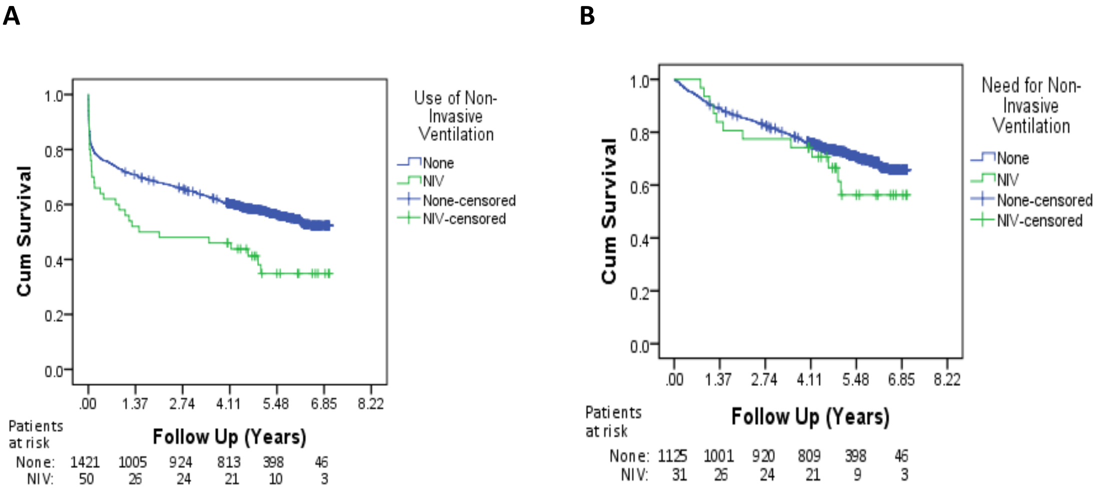

Supplementary Figure 9. Use of non-invasive ventilation (NIV). Kaplan-Meier plots of the proportion of surviving patients over time in years. Patients were categorised by whether or not they required NIV during the index admission. Vertical dashes represent right-censored patients. The number of patients remaining at risk at each time point are presented under the chart. Log-rank analyses are presented to the right of the corresponding chart **a.** Analyses including in-hospital deaths (Log Rank test NIV vs no NIV, P = 0.008). **b.** Analysis excluding in-hospital deaths (Log Rank test NIV vs no NIV, P = 0.301)

A B

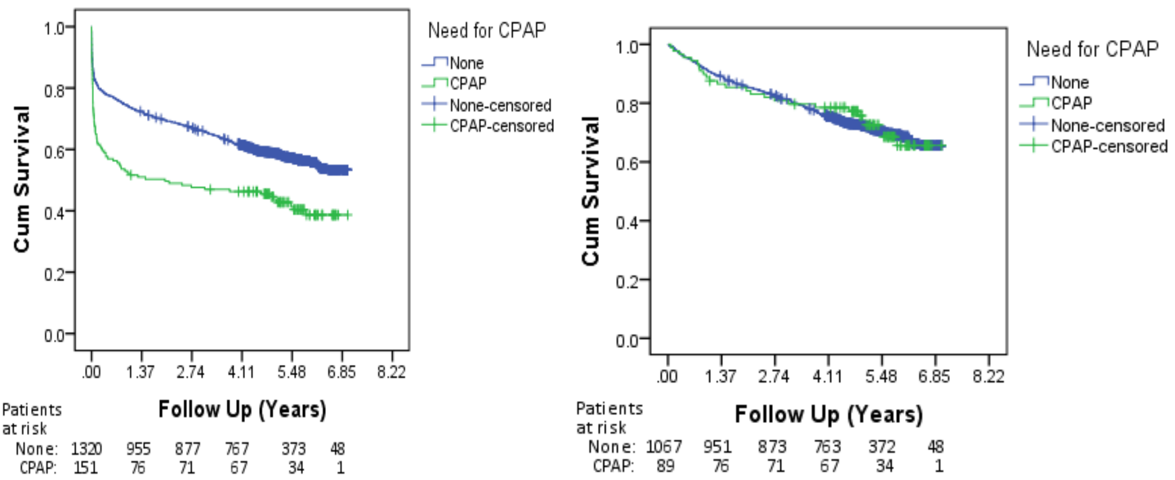

Supplementary Figure 10. Survival according to use of continuous positive airway pressure (CPAP). Kaplan-Meier plots of the proportion of surviving patients over time in years. Patients were categorised by whether they required CPAP during the index admission. Vertical dashes represent right-censored patients. The number of patients remaining at risk at each time point are presented under the chart. Log-rank analyses are presented to the right of the corresponding chart **a.** Analyses including in-hospital deaths (Log Rank test CPAP vs no CPAP, P < 0.001). **b.** Analysis excluding in-hospital deaths (Log Rank test CPAP vs no CPAP, P = 0.930)

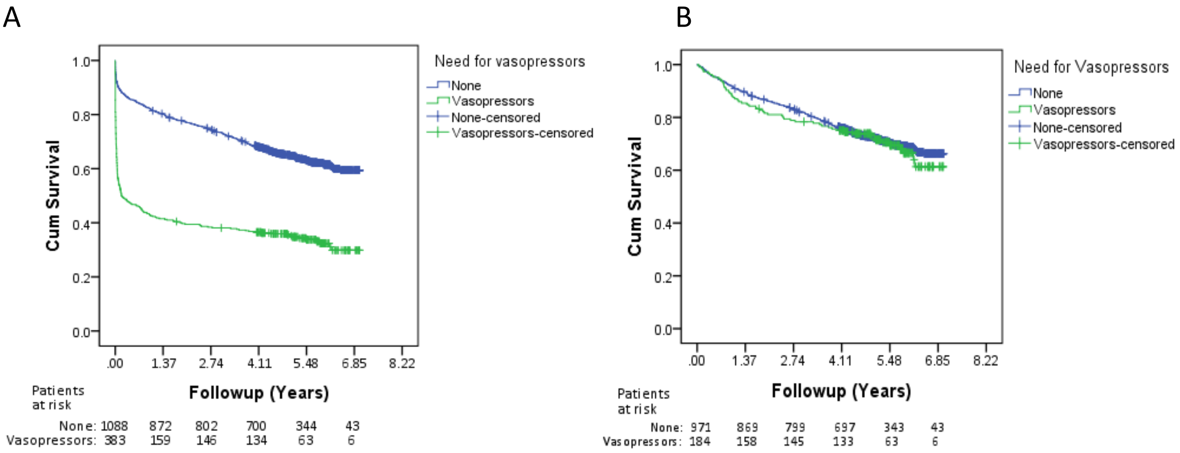

Supplementary Figure 11. Survival according to use of vasopressors. Kaplan-Meier plots of the proportion of surviving patients over time in years. Patients were categorised by whether they required vasopressors during the index admission. Vertical dashes represent right-censored patients. The number of patients remaining at risk at each time point are presented under the chart. Log-rank analyses are presented above the corresponding chart **a.** Analyses including in-hospital deaths (Log Rank test Vasopressors vs no Vasopressors, P < 0.001). **b.** Analysis excluding in-hospital deaths (Log Rank test Vasopressors vs no Vasopressors, P = 0.579)
