# Supplementary material for: Survival and new-onset morbidity after critical care admission for acute pancreatitis in Scotland: a national electronic healthcare record linkage cohort study
Source: BMJ Open. 2018 Dec 14;8(12):e023853. doi: 10.1136/bmjopen-2018-023853 (PMC6303604; doi:10.1136/bmjopen-2018-023853)
Supplement: Author's manuscript [file bmjopen-2018-023853.draft_revisions.pdf]

BMJ Open is committed to open peer review. As part of this commitment we make the peer review history of every article we publish publicly available.

When an article is published we post the peer reviewers' comments and the authors' responses online. We also post the versions of the paper that were used during peer review. These are the versions that the peer review comments apply to.

The versions of the paper that follow are the versions that were submitted during the peer review process. They are not the versions of record or the final published versions. They should not be cited or distributed as the published version of this manuscript.

BMJ Open is an open access journal and the full, final, typeset and author-corrected version of record of the manuscript is available on our site with no access controls, subscription charges or pay-per-view fees (http://bmjopen.bmj.com).

If you have any questions on BMJ Open's open peer review process please email info.bmjopen@bmj.com

BMJ Open is committed to open peer review. As part of this commitment we make the peer review history of every article we publish publicly available.

When an article is published we post the peer reviewers' comments and the authors' responses online. We also post the versions of the paper that were used during peer review. These are the versions that the peer review comments apply to.

The versions of the paper that follow are the versions that were submitted during the peer review process. They are not the versions of record or the final published versions. They should not be cited or distributed as the published version of this manuscript.

BMJ Open is an open access journal and the full, final, typeset and author-corrected version of record of the manuscript is available on our site with no access controls, subscription charges or pay-per-view fees (http://bmjopen.bmj.com).

If you have any questions on BMJ Open's open peer review process please email info.bmjopen@bmj.com

BMJ Open

Survival and new-onset morbidity after critical care admission for acute pancreatitis: a national electronic healthcare record linkage cohort study.

Journal:	*BMJ Open*
Manuscript ID	bmjopen-2018-023853.R1
Article Type:	Research
Date Submitted by the Author:	10-Jul-2018
Complete List of Authors:	Ventre, Chiara; The University of Edinburgh,<br>Nowell, Sian; NHS Scotland National Services Division<br>Graham, Catriona<br>Kidd, Doug; NHS Scotland National Services Division<br>Skouras, Christos; The University of Edinburgh,<br>Mole, Damian; Royal Infirmary of Edinburgh, General Surgery Department; The University of Edinburgh,
**Primary Subject Heading**:	Intensive care
Secondary Subject Heading:	Epidemiology, Gastroenterology and hepatology, Surgery, Diabetes and endocrinology
Keywords:	acute pancreatitis, multiple organ failure, long-term outcomes

SCHOLARONE™
Manuscripts

Title:

Survival and new-onset morbidity after critical care admission for acute pancreatitis: a national electronic healthcare record linkage cohort study.

Authors: Chiara Ventre[1], Sian Nowell[2], Catriona Graham[3], Doug Kidd[2], Christos Skouras[1] and Damian J. Mole[1,4]

Institutions:

1. Surgery, School of Medicine, University of Edinburgh.
2. Electronic Data Research and Innovation Service (eDRIS), NHS National Services Scotland
3. Edinburgh Clinical Trials Unit, University of Edinburgh.
4. MRC Centre for Inflammation Research, University of Edinburgh

Corresponding author:

Professor Damian J Mole, MRC Senior Clinical Fellow, MRC Centre for Inflammation Research, 47 Little France Crescent, Edinburgh EH16 4SA.

damian.mole@ed.ac.uk

For peer review only

Funding Statement. This study did not receive specific funding. Data retrieval costs were covered by existing research funds.

Original Study Protocol. Not applicable

Competing Interests: None declared.

Data sharing statement for supplementary data: Original data are available on request through the NHS National Services Division Safe Haven, subject to an approval process. Further details may be found at http://www.isdscotland.org/Products-and-Services/eDRIS. Please contact the corresponding author in the first instance.

Keywords: acute pancreatitis; long-term outcomes; multiple organ failure

For peer review only - http://bmjopen.bmj.com/site/about/guidelines.xhtml

Abstract:

Introduction: Severe acute pancreatitis (AP) requiring critical care admission (ccAP) impacts negatively on long-term survival.

Objective: To document organ-specific new morbidity and identify risk factors associated with premature mortality after an episode of ccAP.

Design: Cohort study

Setting: Electronic healthcare registries in Scotland.

Participants: ccAP cohort: 1471 patients admitted to critical care with AP between 1st January 2008 and 31st December 2010 followed up until 31st December 2014; population cohort: 3450 individuals from the general population of Scotland frequency matched for age, sex and social deprivation.

Methods: Record linkage of routinely-collected electronic health data with population matching.

Primary and secondary outcome measures: Patient demographics, co-morbidity (Charlson Index), acute physiology, organ support and other critical care data were linked to records of mortality (death certificate data) and new-onset morbidity. Kaplan Meier and Cox regression analyses were used to identify risk factors associated with mortality.

Results: 310 patients with AP died during the index admission. Outcomes were not ascertained for 5 patients, and the deprivation quintile was not known for 6 patients. 340 of 1150 patients in the resulting post-discharge ccAP cohort died during the follow-up period. Greater co-morbidity measured by the Charlson score, prior to ccAP, negatively influenced survival in hospital and after discharge. The odds of developing new-onset diabetes mellitus after ccAP compared to the general population was 10.70 (95% C.I. 5.74 to 19.94). A new diagnosis of myocardial infarction, stroke, heart failure, liver disease, peptic ulcer, renal failure, cancer, peripheral vascular disease and lung disease was more frequent in the ccAP cohort than the general population.

Conclusions: The persistent deleterious impact of severe AP on long-term outcome and survival is multifactorial in origin, influenced by pre-existing patient characteristics and acute episode features. Further mechanistic and epidemiologic investigation is warranted.

Abstract Word Count: 288 words

For peer review only - http://bmjopen.bmj.com/site/about/guidelines.xhtml

Article summary

Strengths and limitations of this study

1. This study is in a large contemporary cohort of patient with AP covering a national population (Scotland).
2. Through secure record linkage, post-ccAP morbidity data are analysed in context of episode specific and pre-existing morbidity data
3. The use of pre-existing national databases resulted in low, but not negligible, amounts of missing data. The amount of missing data might have been further reduced had it been possible to prospectively capture all primary data.
4. Only gallstone aetiology could be specifically examined due to data inaccuracies in the recording of other aetiologies of acute pancreatitis, specifically alcohol excess.
5. The analysis of existing and new comorbidities was limited by the relatively small proportion of patients affected by each comorbidity, and, because co-morbidities derived from SMR01 data only reveal diagnoses made at the time of a hospital admission and therefore are an underestimate of the true population prevalence.

Author contributions. DJM, CS and CV conceived the study. Data retrieval, linkage and secure storage was done by DK and SN. Statistical design and analysis was done by SN and CG. CV and DJM drafted the initial version of the manuscript. All authors revised and approved the final version of the paper. DJM is guarantor.

For peer review only

For peer review only - http://bmjopen.bmj.com/site/about/guidelines.xhtml

Introduction

Acute pancreatitis (AP) is the most common gastrointestinal cause of emergency hospital admission. The incidence of AP is increasing, and in Scotland is 31.8 per 100,000[1-5]. Overall case fatality in AP is 5%[5]. Although most cases are mild and self-limiting, 1 in 4 patients with AP develop multiple organ dysfunction syndrome (AP-MODS) and require critical care admission[6]. AP-MODS is the single most important determinant of death from AP[7], with mortality in patients with AP-MODS reaching 21.7% [5]. Recently, we reported that AP-MODS has detrimental consequences even for those who survive the acute episode, who have a reduced overall survival compared to AP without MODS[8]. Prevention of AP-MODS in humans remains an elusive goal[9], and it is therefore important to characterise the lasting impact on survivors to help maximise their long-term well-being.

AP has many potential causes, of which gallstones and alcohol are most frequently implicated[6 10]. The resulting inflammatory reaction within the pancreas may become over-amplified and precipitate a systemic inflammatory response, shock and organ dysfunction[6 10-13]. There is marked inter-individual heterogeneity in the number of organ systems involved and AP-MODS can affect any organ system, with the respiratory and renal systems most frequently affected [14-17]. Moreover, the severity of organ dysfunction is highly variable, and interventions including invasive ventilation and renal replacement therapy can be required for durations raging from 1 day to 10 weeks[18 19]. AP-MODS determines mortality during the index admission[20] but it is not certain which organ-specific failures are particularly associated with deterioration to death. One study linked hepatic and renal failures with the highest mortality risk[19], whereas another placed greater negative influence after failure of the cardiovascular, pulmonary and gastrointestinal systems[16].

Importantly, it is not completely understood which specific organ deficits may persist in survivors of AP-MODS. AP-MODS has been associated with an increased incidence of diabetes in AP survivors[15 21 22], and age and working status are important in predicting recovery of quality of life and functional capacity[21]. Moreover, given the heterogeneity of the course of AP-MODS, it is unclear if a subgroup of AP-MODS survivors is at particularly high risk of a poor outcome. In the absence of an intervention to prevent AP-MODS, a deeper understanding of the persistent pathophysiological impact left by AP-MODS is needed. Therefore, our aim in this study was to integrate routinely-collected data to investigate the causes and predictors of mortality in the years following an episode of AP requiring critical care admission.

For peer review only - http://bmjopen.bmj.com/site/about/guidelines.xhtml

Methods

Study Design, Data Security and Patient Confidentiality

This retrospective cohort study was conducted in collaboration with eDRIS to facilitate record linkage from multiple national databases. Approval was obtained from the Privacy Advisory Committee of Information Services Division (ISD) Scotland. Information governance and security protocols were adhered throughout the investigation. All primary data was stored securely. Individual informed consent was not required or sought for this study.

Patient and Public involvement

We work closely with our patient and public involvement group, APPLe (Acute Pancreatitis Patient Liaison), to develop our research projects and strategies. This study received general input from members of APPLe as part of a consortium building workshop for the APPreSci Consortium (Acute Pancreatitis Precision Science, www.appresci.com), but APPLe was not involved in the data collection, analysis or manuscript preparation.

Patient Identification & Data Collection

All data were handled according to the Charter for Safe Havens in Scotland[23]. The Scottish Intensive Care Society Audit Group (SICSAG) WardWatcher database[24] was used to identify all patients admitted to critical care with AP between 1st January 2008 and 31st December 2010. AP was defined as any admission to critical care where the primary diagnosis coded by the intensive care senior clinician on duty was recorded as ICD-10 classification K85 (acute pancreatitis). Where an individual was admitted with AP on more than one occasion, the earliest AP episode was taken as the index episode. There were no additional exclusion criteria. We performed a record-linkage analysis of Scottish Morbidity Record (SMR) 01 (General Acute Inpatient and Day Cases), General Register Office (GRO) death records, SICSAG (critical care) and Community Health Index (CHI) databases. General population of Scotland causes of death were obtained from National Records of Scotland Vital Events Tables[25]. Patient outcomes were recorded from the date of their index admission until the end of the follow-up period on 31st December 2014. Those lost to follow up were censored at the point of last known contact. Prior to analysis, data records were linked using unique patient identifiers in order to maintain confidentiality.

For peer review only - http://bmjopen.bmj.com/site/about/guidelines.xhtml

Variables of Interest

The primary outcome of interest was death. The secondary outcomes were cause of death and new-onset morbidity. The following details of the index AP episode were recorded for each patient: gallstone aetiology (from SMR01 data), APACHE II score (acute physiology and chronic health evaluation score, version 2), length of stay in critical care, level of critical care admission (high-dependency unit (HDU) or intensive care unit (ICU))[26], and the requirement for renal replacement therapy, invasive ventilation, non-invasive ventilation, continuous positive airways pressure (CPAP) or vasopressor support (all from SICSAG data). In addition, the following patient characteristics were recorded: age on admission (from SICSAG), gender (from the CHI database), Scottish Index of Multiple Deprivation (SIMD), Charlson score for comorbidity (calculated from SMR01 records for each patient in the 5 years prior to admission)[27] and the number of comorbid conditions contributing to the Charlson score. The cause of death was obtained for each deceased patient and sorted according to ICD-10 code into one of five categories: Cardiovascular/Circulatory, Respiratory, Neoplasia, Digestive/Metabolic, or Other Causes as shown in **Supplementary Table 1**.

Statistical Analysis

IBM SPSS Statistics Version 21 (IBM Corp., Armonk, New York) was used for all analyses. Categorical variables were reported as the absolute frequency and percentage. Continuous variables were reported as the mean ± standard deviation (SD) or the median ± interquartile range (IQR). Kaplan-Meier analysis was used to demonstrate survival with respect to demographic and clinical factors, with the significance of differences assessed using the log-rank (Mantel-Cox) test. Survival was calculated as the time from the index admission to hospital with AP to death; analyses and plots were done for the whole cohort, and for the cohort excluding those members who died during the index episode of AP in order to allow for analysis of long-term outcomes in survivors of the index episode, as specified in the Results and Figures.

The proportional hazards assumption was tested using log(-log) plots of the survival function over time, to confirm that the curves were approximately parallel. A multivariate Cox regression model was constructed to account for potential interactions between predictor variables. Covariates were added to the model using a forward stepwise method. At each step, the covariate found to be most significant was retained in the model. The threshold for retention in models created using SPSS was p=0.01. After each addition, the covariates already present in the model were tested for removal depending on the significance of the likelihood ratio with and without each covariate.

A P value of 0.05 or less was considered significant. Where multiple pairwise comparisons were made – age group (< 20, 20 to 29, 30 to 39, 40 to 49, 50 to 59, 60 to 69, 70-79, > 80 years), Charlson score

For peer review only - http://bmjopen.bmj.com/site/about/guidelines.xhtml

(0, 1, 2, 3, 4+), number of comorbid conditions (0, 1, 2, 3+) – the Bonferroni correction was applied to account for the quantity of comparisons being made.

Secondary Analysis of Associated Comorbidities

A control group was created from the general population using the CHI database register of all patients in Scotland. Controls were frequency matched on deprivation quintile, age and sex. Three controls were selected for every member of the exposed group. Comorbidities at index admission for AP were obtained from the SMR01 computerised acute hospital discharge records (day cases or inpatients) in the 5-year look back period from date of admission for the index AP episode. Comorbidities that developed after discharge were ascertained from admissions after the index admission for AP up to 31st December 2014. The comorbidities that developed after the index event were then compared in the exposed and unexposed groups with a two-sample z test. We calculated the odds ratio (and 95% C.I) of developing each comorbidity given previous admission for AP needing critical care, compared to people with no previous AP admission.

For peer review only

Results

Follow-up and Survival

Between 1st January 2008 and 31st December 2010, 1471 patients were admitted to HDU or ICU with AP. The length of the follow-up period ranged from 4.0 to 7.0 years. The median duration of follow-up from the date of index admission for all AP patients was 4.4 years (IQR 0.6 to 5.6 years) and 4.9 years (IQR 4.0 to 5.8 years) when patients who died in hospital during the index admission were excluded. 16 patients moved to another country and were censored at the point of last known contact. **Figure 1** outlines the demographics of the study population. Demographic data for the cohort are presented in **Table 1**.

		Died during index admission	(% of total)	Survived index admission	(% of total)	Died after hospital discharge	(% of total)
**Gender**							
	**Male**	175	21	475	58	175	21
	**Female**	135	21	341	53	165	26
**Age group**							
	**<20**	0	0	19	95	1	5
	**20-29**	7	9	66	84	6	8
	**30-39**	13	9	112	78	19	13
	**40-49**	22	11	141	72	34	17

For peer review only - http://bmjopen.bmj.com/site/about/guidelines.xhtml

	**50-59**	47	19	157	62	49	19
	**60-69**	72	25	143	49	75	26
	**70-79**	83	27	135	44	86	28
	**80+**	66	37	43	24	70	39
**Scottish Index of Multiple Deprivation (SIMD) Quintile**							
	**1 – most deprived**	86	20	257	59	92	21
	**2**	75	22	179	52	90	26
	**3**	54	21	141	54	67	26
	**4**	51	23	126	56	49	22
	**5 – least deprived**	44	23	107	55	42	22
	**Not known**	0	0	6	100	0	0
**Charlson comorbidity index**							
	**0**	219	21	666	63	167	16
	**1**	41	20	91	44	77	37
	**2**	38	25	41	27	71	47
	**3**	7	18	12	32	19	50
	**4+**	5	36	3	21	6	43
	**Not known**	0	0	3	100	0	0
**Number of comorbid conditions contributing to the Charlson Index**							
	**0**	219	21	666	63	167	16
	**1**	70	21	126	38	136	41
	**2**	18	28	16	25	31	48
	**3+**	3	21	5	36	6	43
	**Not known**	0	0	3	100	0	0

Table 1. Demographic characteristics of the ccAP cohort. The absolute number of patients and row percentages per each category for the following variables of interest are presented: gender, age group, Scottish Index of Multiple Deprivation (SIMD)[28], Charlson co-morbidity index[27], and number of co-morbid conditions contributing to the Charlson index.

During the follow-up period 651 of 1471 (44.3%) patients died. 310 of 651 (47.8%) deaths occurred during the index admission, the outcome of 5 was not known and the deprivation quintile for 6 other patients was not known. The post-discharge critical care AP (ccAP) cohort therefore included 1150 patients, of which 340 died during the follow-up period. As over half of the study cohort survived to the end of follow-up, the median survival time could not be determined. The mean (± standard deviation) survival time in the whole cohort was 4.4 ± 0.1 years and 5.6 ± 0.1 years once in-hospital deaths were excluded.

Cause of death

For peer review only

For peer review only - http://bmjopen.bmj.com/site/about/guidelines.xhtml

In the post-discharge ccAP cohort, neoplasms were the leading cause of death (27.9%), followed by cardiovascular (27.1%) and digestive/metabolic deaths (25.9%) **(Figure 2).** Other causes contributed only 5.3% of the total. This contrasted with the general population of Scotland for which a lower proportion of deaths was attributed to digestive causes (7.3%) while a markedly greater proportion of the general population controls were due to other causes (21.0%) (**Figure 2**).

Predictors of mortality in the post-discharge cohort

Independent negative risk factors for long term survival included age **(Suppl Fig. 1),** a Charlson score of 1 or greater (**Table 2** and **Figure 3a**), and the number of comorbid conditions contributing to the Charlson score (**Table 2** and **Figure 3b**). Survival did not differ significantly with the degree of social deprivation **(Suppl. Fig 2).** Female gender was associated with a shorter survival on univariate analysis, but gender as a risk factor on the multivariate analysis was not significant (**Table 3** and **Figure 3c**). Gallstone aetiology was associated with a lower mortality after discharge (**Table 3**). Comparison with analyses that included in-hospital deaths indicated that these differences emerged post-discharge (**Suppl. Fig 1-6**). Multivariate Cox regression analysis also identified increased age group and Charlson comorbidity score as poor prognostic factors (**Table 3**).

For peer review only

For peer review only - http://bmjopen.bmj.com/site/about/guidelines.xhtml

Risk factor		n	Hazard Ratio	95% CI	p value
**Age**	**under 20**	20	–		
(reference <20 years)	**20 - 29**	72	1.6	0.2, 13.1	0.674
	**30 - 39**	131	2.9	0.4, 21.8	0.296
	**40 - 49**	175	3.9	0.5, 28.5	0.180
	**50 - 59**	206	5.0	0.7, 36.5	0.110
	**60 - 69**	218	7.9	1.1, 57.0	0.040
	**70 - 79**	221	8.7	1.2, 62.4	0.032
	**80+**	113	17.3	2.4, 124.4	0.005
**Gender**	**Male**	650	–		
(reference Male)	**Female**	506	1.2	1.0, 1.5	0.049
**Charlson Score**	**0**	833	–		
(reference 0)	**1**	168	2.6	2.0, 3.4	<0.001
	**2**	112	4.7	3.6, 6.2	<0.001
	**3**	31	3.8	2.4, 6.1	<0.001
	**4 or more**	9	5.3	2.4, 12.0	<0.001
**Number of comorbid conditions**	**0**	833	–		
(reference 0)	**1**	262	3.2	2.6, 4.0	<0.001
	**2**	47	4.5	3.0, 6.7	<0.001
	**3**	11	3.3	1.5, 7.5	0.004
**Length of stay**	**less than 20 days**	1079	–		
(reference <20 days)	**longer than 20 days**	77	0.4	0.2, 0.8	0.006
**Level of critical care**	**ICU**	251	–		
(reference ICU)	**HDU**	905	1.2	1.0, 1.4	0.019

Table 2. Predictors of long-term mortality - univariate regression analysis. The hazard ratio, 95% confidence intervals (CI) and p value of Wald's test are presented for each variable found to significantly affect post-discharge survival on univariate regression analysis. p<0.05 was considered significant. The reference category for each variable is appended. Age has been transformed to a categorical variable for the purposes of the analysis. ***n****: number of patients per category;* ***HDU****: High-Dependency Unit;* ***ICU****: Intensive Care Unit.*

For peer review only - http://bmjopen.bmj.com/site/about/guidelines.xhtml

Risk Factor	Hazard Ratio	95% CI	p value
**Age**	1.0	1.0, 1.1	<0.001
**Charlson score**	1.5	1.4, 1.7	<0.001
**Female gender**	1.2	1.0, 1.5	0.058
**Gallstone aetiology**	0.7	0.6, 0.9	0.003

Control variables not in the final model: renal replacement therapy, invasive ventilation, non-invasive ventilation, vasopressor use and SIMD (deprivation index)

*Age group as defined in Table 2

Table 3. Final model of prognostic factors of post-discharge mortality. The hazard ratio, 95% confidence intervals and p value of Wald's test are presented for each variable retained in the final multivariate Cox regression model. p<0.05 was considered significant.

A significant relationship was observed between the requirement for renal replacement therapy, respiratory or circulatory support and an increased risk of death when in-hospital deaths were included **(Suppl. Fig 7-11)**. However, no correlation between mortality and any of the aforementioned medical interventions, or gallstone aetiology, was observed when considering only post-discharge outcomes **(Suppl. Figs 6-11)**. Long-term survival of those who survived the index episode was significantly better where the length of stay in critical care during the index episode exceeded 20 days, compared to admissions of 0-4 or 10-19 days (**Figure 4a**). The critical care setting was important – patients with AP-MODS admitted to ICU, and who survived that event, had better long-term survival compared to those who were admitted to HDU and survived (**Figs 4b** and **4c,** and **Table 2**).

Development of new specific comorbidities

Patients in the ccAP cohort were significantly more likely to develop a range of cardiovascular, gastrointestinal, pulmonary and neoplastic conditions than matched controls (**Table 4**). A particularly high risk of developing new-onset diabetes was noted (OR 10.70, 95% C.I. 5.74 to 19.94), with 3.9% of the ccAP cohort developing new diabetes during the follow-up period compared to 0.4% of the matched control group. The risk of developing renal disease requiring hospital admission was also markedly increased (OR 9.15, 95% C.I. 2.95 to 28.43), but whether this was confounded by new or existing diabetes could not be ascertained, and the numbers of people affected by renal disease was small in both cohorts. Risks for developing other comorbidities are presented in **Table 4**.

For peer review only - http://bmjopen.bmj.com/site/about/guidelines.xhtml

Comorbidity	Co-morbidities at SICSAG admission			New co-morbidities developed after discharge					
	% of AP cohort	% of controls	P value	% of AP cohort	% of controls	P value	Odds Ratio[1]	95% CI Lower	95% CI Upper
AMI	2.7	1.2	<0.001	3.7	1.8	<0.001	2.09	1.4	3.12
cerebral vascular accident	1.9	1.7	0.555	3.5	2.2	0.021	1.58	1.07	2.35
Congestive heart failure	2.3	0.4	<0.001	2.5	0.8	<0.001	3.11	1.83	5.28
Connective tissue disorder	0.8	0.3	0.024	0.1	0.1	0.641	0.6	0.07	5.16
Diabetes & Diabetes complications	1.8	0.2	<0.001	3.9	0.4	<0.001	10.7	5.74	19.94
Liver disease & Severe Liver Disease	0.5	0.1	0.004	0.6	0.1	0.003	5.3	1.55	18.14
Peptic ulcer	1.9	0.3	<0.001	1.6	0.3	<0.001	5.06	2.38	10.74
Peripheral vascular disease	1.5	0.6	0.007	1.3	0.5	0.002	2.86	1.41	5.81
Pulmonary disease	3.8	1.1	<0.001	2.5	1.6	0.033	1.65	1.04	2.62
Cancer & Metastatic cancer	7.2	2.8	<0.001	8.4	5.4	<0.001	1.62	1.25	2.11
Renal disease	1	0.2	0.001	1.1	0.1	<0.001	9.15	2.95	28.43

Table 4. Baseline comorbid status and risk of developing new comorbidities after the index AP episode. The percentage of patients and controls who developed each specified comorbidity in the 5 years before and 5 years after the index AP episode are presented. The odds ratio, as well as the 95% confidence intervals for the development of each comorbidity after the AP episode are included. Total number of patients from the AP cohort: 1150, total number of controls: 3450. The p values were obtained by applying the 2-sample Z-test. $p<0.05$ was considered significant. ***SICSAG****: The Scottish Intensive Care Society Audit Group software;* ***AMI****: acute myocardial infarction.*

In addition to evaluating the risk of new onset co-morbidity, we examined whether the baseline comorbidities of the population who experience an episode of AP might be different to the general population (**Table 4**). At the time of presentation with their index episode, patients with AP needing critical care were significantly more likely to have existing co-morbidities that included cardiac, lung, renal or peripheral vascular disease, heart failure, connective tissue disorders, peptic ulcers and cancer than matched general population controls. ccAP therefore appears to be a feature associated with members of the population that are already less healthy.

For peer review only - http://bmjopen.bmj.com/site/about/guidelines.xhtml

Discussion

This retrospective data linkage cohort study aims to investigate the causes and predictors of mortality in the years following an episode of AP requiring critical care admission. In so doing, statistically significant differences in frequency of the causes of death have been demonstrated between the ccAP patient cohort and the general population. In addition, the results indicate that long-term prognosis after a critical care admission for AP is influenced to a greater extent by age at the time of index AP admission and existing comorbidity than by specific features of the index AP episode. New-onset comorbidity, particularly diabetes, is more frequent following ccAP than in the general population. We acknowledge that ccAP patients may have additional diagnoses made because these individuals seek more frequent contact with healthcare and therefore have the opportunity to get diagnosed with comorbidities. Furthermore, co-morbidities derived from SMR01 data only reveal diagnoses made at the time of a hospital admission and are therefore an underestimate of the true population prevalence. From our analysis it is not possible to discern whether those individuals were destined to develop those co-morbidities regardless of their episode of AP, especially given that the AP cohort is less healthy overall than the matched general population. A similar association between MODS and mortality has been demonstrated in patients who have sustained trauma[29]. Our results support our previously-observed concept that ccAP is associated with a persistent deleterious impact on survivors. Inter-individual heterogeneity in the clinical course of the AP critical care episode was not associated with any organ-specific long-term outcomes in this analysis, but we acknowledge that our approach was limited in the ability to discriminate these with certainty.

Together, these findings lend weight to the hypothesis that severe AP episodes do not fully resolve, with particular emphasis on the impact of the associated systemic dysfunction. Our study has added data and analysis to underpin this concept by investigating the specific details of the deleterious legacy of ccAP. Given that the variation in causes of death is largely due to an increased proportion of deaths from metabolic disease, it is reasonable to infer that AP mediates the long-term effects primarily through ongoing metabolic pathology. This result concurs with outcomes in a Danish cohort that demonstrated a marked increase in deaths from digestive system causes in AP survivors compared to the general population[30]. Exact mechanisms underpinning the metabolic disturbance remain to be elucidated and will almost certainly require a prospective experimental medicine study. Taken together, the reported high incidence of diabetes mellitus after AP, the correlation of AP severity with lasting pancreatic exocrine dysfunction (as shown by others), and the negative effect of exocrine pancreatic insufficiency, suggest that impairment of the endocrine and exocrine pancreas is the main driver of the lasting overall dysfunction[15 21 22]. Additionally, it is reasonable to expect that aspects of the acute systemic dysfunction associated with MODS, for example insulin resistance and mitochondrial dysfunction, fail to resolve entirely[31], although we have not tested this experimentally in this study.

For peer review only - http://bmjopen.bmj.com/site/about/guidelines.xhtml

Identifying predictors of post-discharge mortality will facilitate appropriate targeting of preventative interventions. The identification of greater pre-existing comorbidity as a key negative predictive factor is consistent with previous research correlating more extensive comorbid disease with a worse prognosis after critical illness[32]. In the present study, our observation that post-discharge outcomes were better for ICU than HDU patients by univariate analysis could be explained by comorbidity – those requiring ICU admission theoretically experience the worst AP episodes, and therefore only relatively fitter individuals may survive to discharge. In contrast, those with greater comorbidity, and hence a higher risk of later mortality, may survive an AP episode managed in HDU. We acknowledge that the preceding statement is somewhat speculative, despite being highly plausible. A similar argument may explain the association of better long-term outcomes with a critical care stay exceeding 20 days, in that individuals with less associated comorbidity at AP onset may be more resilient to a prolonged critical care admission. This finding is in contrast to data on long-term survival in all ICU patients, where prolonged admission was associated with a shorter long-term survival[33 34]. However, the positive association between duration of organ support in ICU and post-ccAP survival is likely subject to iatrogenic influences. For example, a willingness to persist with organ support in critical care by the physician-led multidisciplinary team in those without significant medical comorbidity prior to ICU admission, may result organ support being continued for longer, a form of survivor treatment selection bias[35].

We observed that gallstone aetiology had a less negative effect on prognosis. While this might be explained by the additional burden of morbidity and mortality carried by alcohol misuse, the other key cause of AP[36-38], our data implies that gallstone AP requiring critical care has less severe long-term consequences. This is in contrast to previous studies by others, where, in acute AP, a gallstone aetiology was associated with more severe MODS than alcohol-induced cases [39], and separately, no effect of gallstone aetiology on long-term prognosis after accounting for the detrimental impact of alcohol[30]. It is important to note that alcohol-related AP was not specifically known in our study population.

A strength of this study is that the applicability of these observations to all ccAP survivors has been enhanced by using primary data from a population basis rather than a single centre, in collaboration with Farr@Scotland. This UK-wide network was created to facilitate the storage, sharing and analysis of population and health-related datasets in an environment that protects patient confidentiality and data security[40]. The employment of this resource facilitated the achievement of larger sample population than would have been possible with a single-centre study and reduced the risk of the results being modified by, for example, regional variations in treatment or population demographics[41 42].

We acknowledge specific limitations of our study. Firstly, the use of pre-existing national databases requires an acceptance of low amounts of missing data that might have been avoided had it been possible to prospectively capture all primary data. However, the expense and time needed to do that would make a study of this size extremely unwieldy, and we regard our approach to be preferable to that, at this stage.

For peer review only - http://bmjopen.bmj.com/site/about/guidelines.xhtml

The incidence of missing data was very low, with the exception of APACHE II scores. In order to overcome data inaccuracies, only gallstone aetiology was specifically noted. Our experience of using these records to correctly attribute alcohol aetiology have been not sufficiently reliable as a foundation for a robust analysis. Though not possible within the limitations of the current study, this will be an important consideration in advancing this research. Because there was uncertainty in our attribution of ccAP aetiology, with the exception of those diagnosed with gallstones, coupled with our use of relatively healthy controls from the general population, we were unable to analyse future causes of death and survival bias based on that factor. Insufficient detail in this dataset precluded a robust analysis regarding the frequency of recurrent episodes of AP in the cohort, because it was not possible to distinguish repeat hospital admissions due to complications arising from the index episode from true *de novo* recurrent episodes. This would be addressed by a prospective study. Finally, the analysis of existing and new comorbidities was limited by relatively low proportions of patients affected by each comorbidity. The value of replicating these findings using larger patient cohorts would need to be weighed against the practical challenges but should be considered. Further clarification of this phenomenon, and the impact on other body systems, is in progress through a prospective experimental medicine cohort study[43]. The identification of specific goals for intervention in the follow-up period after AP will require that detailed assessment of alterations in patients' physiological status over time.

In conclusion, long-term outcomes after AP requiring critical care are influenced by pre-existing patient characteristics and specific factors associated with an episode of critical care admission. Persisting metabolic derangement after ccAP is associated with premature death. The persistent deleterious impact of severe AP on survival is multifactorial, and further mechanistic and epidemiologic investigation is required.

Acknowledgments

DJM acknowledges the support of the MRC through a Senior Clinical Fellowship. We are grateful to members of APPLe for their personal insight and advise on our overall programme of work.

For peer review only - http://bmjopen.bmj.com/site/about/guidelines.xhtml

References

1. O'Farrell A, Allwright S, Toomey D, et al. Hospital admission for acute pancreatitis in the Irish population, 1997–2004: could the increase be due to an increase in alcohol-related pancreatitis? *Journal of Public Health* 2007;29(4):398-404.
2. Fagenholz PJ, Castillo CF-D, Harris NS, et al. Increasing United States Hospital Admissions for Acute Pancreatitis, 1988–2003. *Annals of Epidemiology* 2007;17(7):491.e1-91.e8. doi: http://dx.doi.org/10.1016/j.annepidem.2007.02.002
3. Sandzén B, Rosenmüller M, Haapamäki MM, et al. First attack of acute pancreatitis in Sweden 1988 – 2003: incidence, aetiological classification, procedures and mortality – a register study. *BMC Gastroenterology* 2009;9:18-18. doi: 10.1186/1471-230X-9-18
4. McKay C, Evan S, Sinclair M, et al. High early mortality rate from acute pancreatitis in Scotland: 1984-1995. *Br J Surg* 1999;86(10):1302-05.
5. Mole D, Gungabissoon U, Johnston P, et al. Identifying risk factors for progression to critical care admission and death among individuals wih acute pancreatitis by utilizing linked Scottish healthcare databases. *Lancet* 2015
6. Hayden P, Wyncoll D. Severe acute pancreatitis. *Current Anaesthesia & Critical Care* 2008;19(1):1-7. doi: http://dx.doi.org/10.1016/j.cacc.2007.01.009
7. Andersson R, Andersson B, Haraldsen P, et al. Incidence, management and recurrence rate of acute pancreatitis. *Scandinavian Journal of Gastroenterology* 2004;39(9):891-94.
8. Skouras C, Hayes AJ, Williams L, et al. Early organ dysfunction affects long-term survival in acute pancreatitis patients. *HPB* 2014;16(9):789-96. doi: 10.1111/hpb.12259
9. Johnson C. Organ Failure and Acute Pancreatitis. In: Forsmark CE, Gardner TB, eds. Prediction and Management of Severe Acute Pancreatitis: Springer New York 2015:15-27.
10. Wang G-J, Gao C-F, Wei D, et al. Acute pancreatitis: Etiology and common pathogenesis. *World Journal of Gastroenterology : WJG* 2009;15(12):1427-30. doi: 10.3748/wjg.15.1427
11. Raraty MT, Connor S, Criddle D, et al. Acute pancreatitis and organ failure: Pathophysiology, natural history, and management strategies. *Current Gastroenterology Reports* 2004;6(2):99-103. doi: 10.1007/s11894-004-0035-0
12. Norman Md J. The Role of Cytokines in the Pathogenesis of Acute Pancreatitis. *The American Journal of Surgery* 1998;175(1):76-83. doi: http://dx.doi.org/10.1016/S0002-9610(97)00240-7
13. Neoptolemos JP. Compartments That Cause the Real Damage in Severe Acute Pancreatitis. *American Journal of Respiratory and Critical Care Medicine* 2003;168(2):141-42. doi: 10.1164/rccm.2305003
14. Johnson C, Abu-Hilal M, Group MotBAPS. Persistent organ failure during the first week as a marker of fatal outcome in acute pancreatitis. *Gut* 2004;53(9):1340-44.
15. Appelros S, Lingren S, Borgstrom A. Short and long-term outcome of severe acute pancreatitis. *Eur J Surg* 2001;167(4):281-86.
16. Zhu A, Shi J, Sun X. Organ failure associated with severe acute pancreatitis. *World Journal of Gastroenterology* 2003;9(11):2570-73.
17. Mole D, Olabi B, Robinson V. Incidence of individual organ dysfunction in fatal acute pancreatitis: analysis of 1024 death records. *HPB* 2009;11(2):166-70.
18. Halonen KI, Leppäniemi AK, Puolakkainen PA, et al. Severe Acute Pancreatitis: Prognostic Factors in 270 Consecutive Patients. *Pancreas* 2000;21(3)
19. Halonen K, Pettilä V, Leppäniemi A, et al. Multiple organ dysfunction associated with severe acute pancreatitis. *Critical Care Medicine* 2002;30(6):1274-79.
20. Banks P, Bollen T, Dervenis C. Classification of acute pancreatitis - 2012: revision of the Atlanta classification and definitions by international consensus. *Gut* 2013;62(1):102-11.
21. Halonen K, Pettila V, Leppaniemi A. Long-term health-related quality of life in survivors of severe acute pancreatitis. *Intensive Care Med* 2003;29(5):782-86.
22. Doepel M, Eriksson J, Halme L, et al. Good long-term results in patients surviving severe acute pancreatitis. *British Journal of Surgery* 1993;80(12):1583-86. doi: 10.1002/bjs.1800801229

For peer review only - http://bmjopen.bmj.com/site/about/guidelines.xhtml

23. Government S. Charter for Safe Havens in Scotland: Handling Unconsented Data from National Health Service Patient Records to Support Research and Statistics. 2015 [Available from: http://www.gov.scot/Publications/2015/11/4783/4.
24. SICSAG [Available from: http://www.sicsag.scot.nhs.uk/index.html accessed January 15 2016.
25. Vital Events Reference Tables 2015 [Available from: http://www.nrscotland.gov.uk/statistics-and-data/statistics/statistics-by-theme/vital-events/vital-events-reference-tables accessed October 30 2015.
26. Society IC. Levels of Critical Care for Adult Patients [Available from: http://www.ics.ac.uk/ICS/Guidelines___Standards/ICS/guidelines-and-standards.aspx?hkey=4ed20a1c-1ff8-46e0-b48e-732f1f4a90e2.
27. Charlson ME, Pompei P, Ales KL, et al. A new method of classifying prognostic comorbidity in longitudinal studies: Development and validation. *Journal of Chronic Diseases* 1987;40(5):373-83. doi: http://dx.doi.org/10.1016/0021-9681(87)90171-8
28. Government S. Scottish Index of Multiple Deprivation 2012: A National Statistics Publication for Scotland. Edinburgh, 2012.
29. Ulvik A, Kvåle R, Wentzel-Larsen T, et al. Multiple organ failure after trauma affects even long-term survival and functional status. *Critical Care* 2007;11(5):R95.
30. Nøjgaard C. Prognosis of acute and chronic pancreatitis: a 30 year follow up of a Danish cohort. *Dan Med Bull* 2010;57(12):B4228.
31. Singer M, De Santis V, Vitale D, et al. Multiorgan failure is an adaptive, endocrine-mediated, metabolic response to overwhelming systemic inflammation. *The Lancet* 2004;364(9433):545-48. doi: http://dx.doi.org/10.1016/S0140-6736(04)16815-3
32. Zilberberg MD, Epstein SK. Acute Lung Injury in the Medical ICU. *American Journal of Respiratory and Critical Care Medicine* 1998;157(4):1159-64. doi: 10.1164/ajrccm.157.4.9704088
33. Williams TA, Dobb GJ, Finn JC, et al. Determinants of long-term survival after intensive care. *Crit Care Med* 2008;36(5):1523-30. doi: 10.1097/CCM.0b013e318170a405
34. Roberts SE, Thorne K, Evans PA, et al. Mortality following acute pancreatitis: social deprivation, hospital size and time of admission: record linkage study. *BMC Gastroenterol* 2014;14:153. doi: 10.1186/1471-230X-14-153
35. van Walraven C, Davis D, Forster AJ, et al. Time-dependent bias was common in survival analyses published in leading clinical journals. *J Clin Epidemiol* 2004;57(7):672-82. doi: 10.1016/j.jclinepi.2003.12.008
36. Statistics on Alcohol 2016 [Available from: https://www.alcoholconcern.org.uk/help-and-advice/statistics-on-alcohol/ accessed January 10 2016.
37. Yancik R, Wesley MN, Ries LAG, et al. Comorbidity and age as predictors of risk for early mortality of male and female colon carcinoma patients. *Cancer* 1998;82(11):2123-34. doi: 10.1002/(SICI)1097-0142(19980601)82:11<2123::AID-CNCR6>3.0.CO;2-W
38. Singh VK, Bollen TL, Wu BU, et al. An Assessment of the Severity of Interstitial Pancreatitis. *Clinical Gastroenterology and Hepatology* 2011;9(12):1098-103. doi: http://dx.doi.org/10.1016/j.cgh.2011.08.026
39. de Beaux AC, Palmer KR, Carter DC. Factors influencing morbidity and mortality in acute pancreatitis; an analysis of 279 cases. *Gut* 1995;37(1):121-26.
40. Farr Institute @ Scotland. About. 2015 [cited 2015 November 14]. Available from: http://www.farrinstitute.org/centre/Scotland/3_About.html accessed November 14 2015.
41. Olmos D, Ang JE, Gomez-Roca C, et al. Pitfalls and limitations of a single-centre, retrospectively derived prognostic score for Phase I oncology trial participants – Reply to Fussenich et al.: A new, simple and objective prognostic score for Phase I cancer patients. *European Journal of Cancer* 2012;48(4):594-96. doi: http://dx.doi.org/10.1016/j.ejca.2011.11.004
42. Altman DG, Bland JM. Generalisation and extrapolation. *BMJ* 1998;317(7155):409-10.
43. Mole D, Boyd J. Resolution of Organ Injury in Acute Pancreatitis - RESORP (RESORP) 2017 [Available from: https://clinicaltrials.gov/ct2/show/NCT03342716 accessed March 14 2018 2018.

For peer review only - http://bmjopen.bmj.com/site/about/guidelines.xhtml

Figure Legends

Figure 1. Study Population Demographics. Visual representation of demographic characteristics for the study cohort (n = 1471 patients) with stacked bar-charts. In all panels, the absolute number of patients per variable category is charted: in red are patients who died in hospital, those who died post-discharge in blue, and those surviving to the end of follow-up in grey. The following attributes of the cohort are depicted sequentially in each respective panel: **A**. Standard Index of Multiple Deprivation (SIMD), **B**. Charlson score, **C.** Gender, **D.** Age (transformed to a categorical variable), and **E.** Number of comorbid conditions contributing to the Charlson score.

Figure 2. Comparison of the Causes of Death between the ccAP cohort and the general population. Bar chart of the causes of death of the post-discharge ccAP cohort (341 deaths – blue colour), and of those of matched controls from the general population of Scotland (381,060 deaths – red colour). The causes of death have been grouped into one of the following categories, according to the ICD-10 code: Cardiovascular/Circulatory, Respiratory, Neoplasia, Digestive/Metabolic, or Other Causes (Suppl. Table 1).

Figure 3. Post-discharge survival of ccAP patients – Patient characteristics. Kaplan-Meier survival plots for post-discharge ccAP patients, grouped by: **A.** Charlson score, **B**. Number of comorbid conditions contributing to each calculated Charlson score, **C**. Gender. The numbers of patients at risk at each time point are displayed. For each plot, in-hospital deaths have been excluded and time zero corresponds to point of discharge. Vertical dashes represent right-censored patients.

Figure 4. Post-discharge survival of ccAP patients – Nature of the critical care admission. Kaplan-Meier survival plots for post-discharge ccAP patients, grouped by: **A.** Duration of critical care admission, **B**. Level of critical care (all ccAP patients), **C.** Level of critical care (post-ccAP patients, excluding in-hospital deaths). The numbers of patients at risk at each time point are displayed for each plot. Vertical dashes represent right-censored patients.

For peer review only - http://bmjopen.bmj.com/site/about/guidelines.xhtml

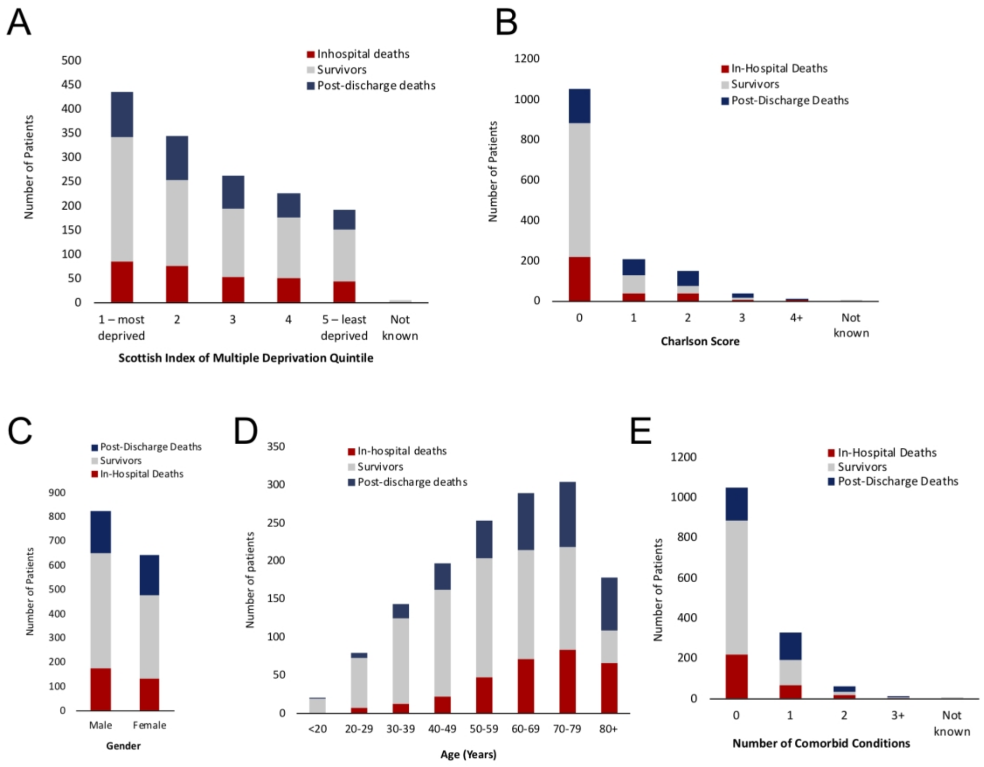

Figure 1. Study Population Demographics. Visual representation of demographic characteristics for the study cohort (n = 1471 patients) with stacked bar-charts. In all panels, the absolute number of patients per variable category is charted: in red are patients who died in hospital, those who died post-discharge in blue, and those surviving to the end of follow-up in grey. The following attributes of the cohort are depicted sequentially in each respective panel: A. Standard Index of Multiple Deprivation (SIMD), B. Charlson score, C. Gender, D. Age (transformed to a categorical variable), and E. Number of comorbid conditions contributing to the Charlson score.

171x135mm (300 x 300 DPI)

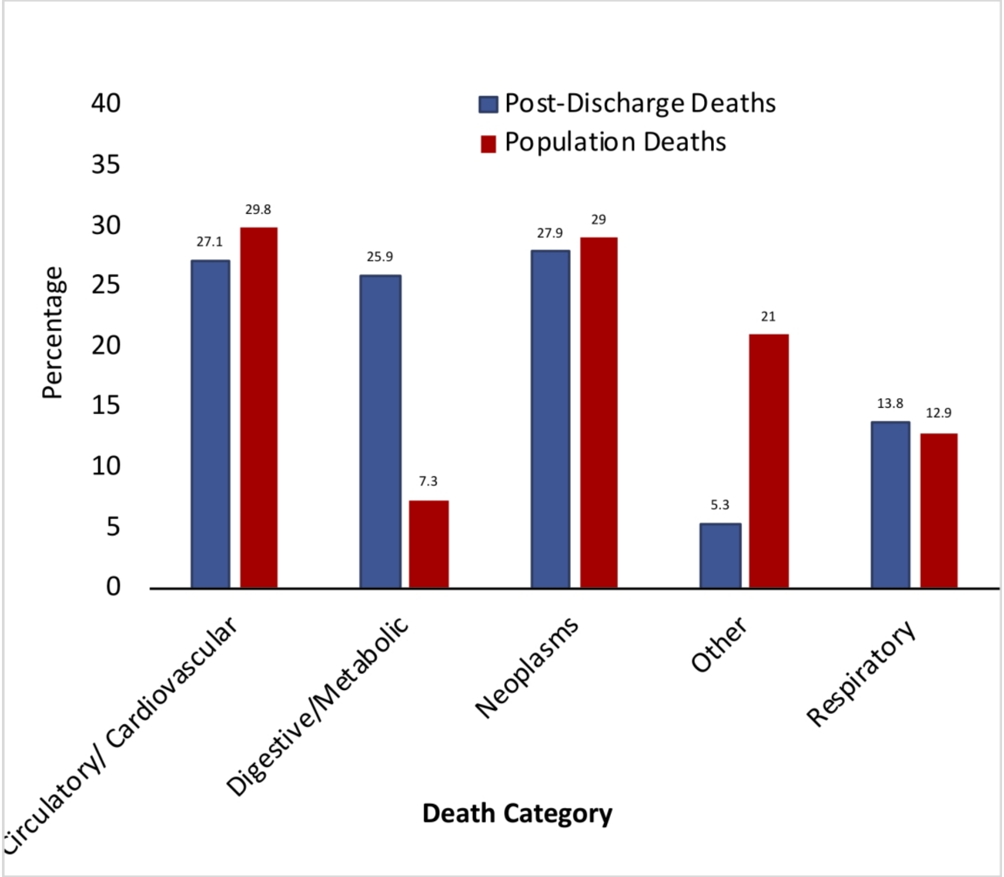

Figure 2. Comparison of the Causes of Death between the ccAP cohort and the general population. Bar chart of the causes of death of the post-discharge ccAP cohort (341 deaths – blue colour), and of those of matched controls from the general population of Scotland (381,060 deaths – red colour). The causes of death have been grouped into one of the following categories, according to the ICD-10 code: Cardiovascular/Circulatory, Respiratory, Neoplasia, Digestive/Metabolic, or Other Causes (Suppl. Table 1).

168x148mm (300 x 300 DPI)

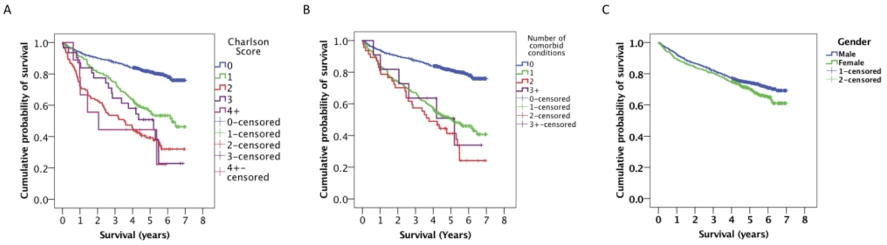

Number at risk/year

	0	1	2	3	4	5	6	7
0	833	782	745	717	685	443	192	0
1	168	152	133	122	104	63	34	0
2	112	80	70	59	47	26	9	0
3	31	26	23	19	17	9	2	0
4+	9	6	4	3	2	1	1	0

Number at risk/year

	0	1	2	3	4	5	6	7
0	833	782	745	717	685	443	192	0
1	261	219	191	172	147	86	42	0
2	47	37	32	26	22	11	2	0
3+	11	9	8	6	5	3	2	0

Number at risk/year

	0	1	2	3	4	5	6	7
Male	650	600	558	523	490	311	136	0
Female	506	452	423	403	374	237	138	0

Figure 3. Post-discharge survival of ccAP patients – Patient characteristics. Kaplan-Meier survival plots for post-discharge ccAP patients, grouped by: A. Charlson score, B. Number of comorbid conditions contributing to each calculated Charlson score, C. Gender. The numbers of patients at risk at each time point are displayed. For each plot, in-hospital deaths have been excluded and time zero corresponds to point of discharge. Vertical dashes represent right-censored patients.

297x209mm (300 x 300 DPI)

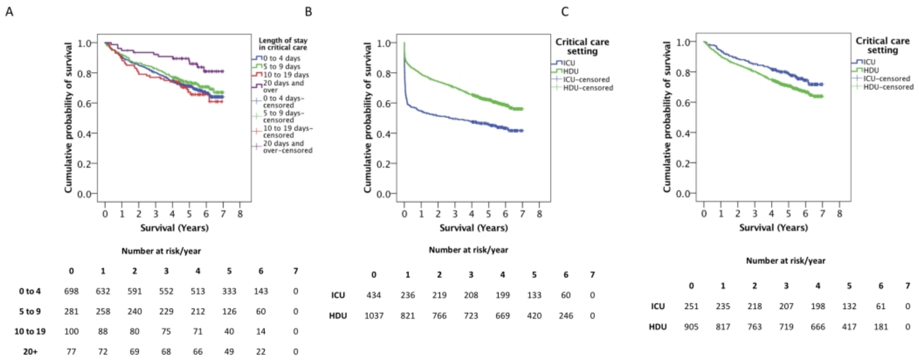

Figure 4. Post-discharge survival of ccAP patients – Nature of the critical care admission. Kaplan-Meier survival plots for post-discharge ccAP patients, grouped by: A. Duration of critical care admission, B. Level of critical care (all ccAP patients), C. Level of critical care (post-ccAP patients, excluding in-hospital deaths). The numbers of patients at risk at each time point are displayed for each plot. Vertical dashes represent right-censored patients.

297x209mm (300 x 300 DPI)

Supplementary data.

Title: Survival and new-onset morbidity after critical care admission for acute pancreatitis: a national electronic healthcare record linkage study.

Authors: Chiara Ventre, Sian Nowell, Cat Graham, Doug Kidd, Christos Skouras and Damian J. Mole

Supplementary material includes:

1 table

11 supplementary figures

On 13 pages

For peer review only

Supplementary Table 1. Categorisation of Causes of Death. The ICD-10 categories for cause of death were allocated to six groups as shown.

Category of death	ICD 10 Code
Circulatory/Cardiovascular system	IX Diseases of the circulatory system
Digestive/Metabolic System	III Diseases of the blood and blood-forming organs and certain disorders involving the immune mechanism<br>*D50-D53 Nutritional anaemias*
	IV Endocrine, nutritional and metabolic diseases
	XI Diseases of the digestive system
	XVIII Symptoms, signs and abnormal clinical and laboratory findings, not elsewhere classified<br>*R10-R19 Symptoms and Signs involving the digestive system and abdomen*
Neoplasms	II Neoplasms
Respiratory System	X Diseases of the Respiratory System
Other	I Certain infectious and parasitic diseases
	III Diseases of the blood and blood-forming organs and certain disorders involving the immune mechanism<br>*Excluding D50-D53*
	V Mental and behavioural disorders
	VI Diseases of the nervous system
	VII Diseases of the eye and adnexa
	VIII Diseases of the ear and mastoid process
	XII Diseases of the skin and subcutaneous tissue
	XIII Diseases of the musculoskeletal system and connective tissue
	XIV Diseases of the genitourinary system
	XV Pregnancy, childbirth and the puerperium
	XVI Certain conditions originating in the perinatal period
	XVII Congenital malformations, deformations and chromosomal abnormalities
	XVIII Symptoms, signs and abnormal clinical and laboratory findings, not elsewhere classified<br>*Excluding R10-R19*
	XIX Injury, poisoning and certain other consequences of external causes
	XX External causes of morbidity and mortality
	XXI Factors influencing health status and contact with health services
	XXII Codes for special purposes

For peer review only

A

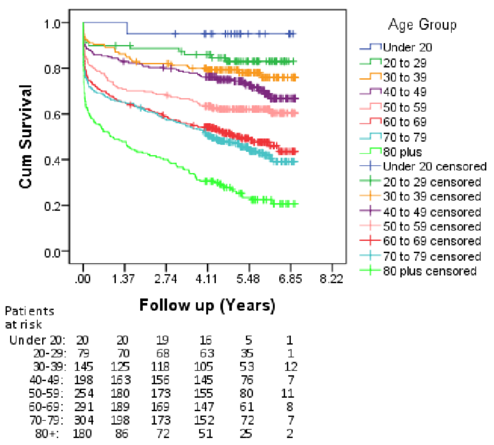

B

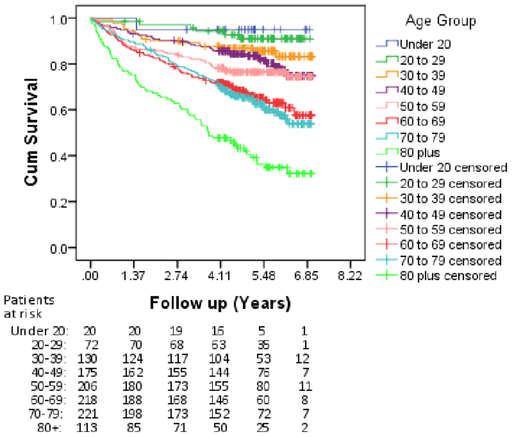

Pairwise Comparisons – including in hospital deaths

	Age group	Under 20		20 to 29		30 to 39		40 to 49		50 to 59		60 to 69		70 to 79		80 plus	
		Chi-Square	Sig.	Chi-Square	Sig.	Chi-Square	Sig.	Chi-Square	Sig.	Chi-Square	Sig.	Chi-Square	Sig.	Chi-Square	Sig.	Chi-Square	Sig.
Log Rank (Mantel-Cox)	Under 20			1.614	.204	2.907	.088	4.199	.040	7.380	.007	11.599	.001	13.092	.000	24.091	.000
	20 to 29	1.614	.204			.954	.329	3.962	.047	11.683	.001	25.914	.000	32.015	.000	66.499	.000
	30 to 39	2.907	.088	.954	.329			1.486	.223	10.898	.001	31.203	.000	40.173	.000	92.223	.000
	40 to 49	4.199	.040	3.962	.047	1.486	.223			5.256	.022	25.194	.000	35.378	.000	92.343	.000
	50 to 59	7.380	.007	11.683	.001	10.898	.001	5.256	.022			8.366	.004	15.589	.000	63.022	.000
	60 to 69	11.599	.001	25.914	.000	31.203	.000	25.194	.000	8.366	.004			1.094	.296	30.182	.000
	70 to 79	13.092	.000	32.015	.000	40.173	.000	35.378	.000	15.589	.000	1.094	.296			21.152	.000
	80 plus	24.091	.000	66.499	.000	92.223	.000	92.343	.000	63.022	.000	30.182	.000	21.152	.000		

Pairwise Comparisons

		Under 20		20 to 29		30 to 39		40 to 49		50 to 59		60 to 69		70 to 79		80 plus	
	Age group	Chi-Square	Sig.	Chi-Square	Sig.	Chi-Square	Sig.	Chi-Square	Sig.	Chi-Square	Sig.	Chi-Square	Sig.	Chi-Square	Sig.	Chi-Square	Sig.
Log Rank (Mantel-Cox)	Under 20			.195	.659	1.212	.271	1.981	.159	3.281	.070	5.997	.014	6.718	.010	15.130	.000
	20 to 29	.195	.659			1.730	.188	4.552	.033	7.764	.005	17.852	.000	21.251	.000	49.740	.000
	30 to 39	1.212	.271	1.730	.188			.986	.321	4.151	.042	16.184	.000	20.474	.000	59.844	.000
	40 to 49	1.981	.159	4.552	.033	.986	.321			1.272	.259	12.308	.000	17.081	.000	60.511	.000
	50 to 59	3.281	.070	7.764	.005	4.151	.042	1.272	.259			5.838	.016	9.458	.002	47.244	.000
	60 to 69	5.997	.014	17.852	.000	16.184	.000	12.308	.000	5.838	.016			.326	.568	22.791	.000
	70 to 79	6.718	.010	21.251	.000	20.474	.000	17.081	.000	9.458	.002	.326	.568			19.713	.000
	80 plus	15.130	.000	49.740	.000	59.844	.000	60.511	.000	47.244	.000	22.791	.000	19.713	.000		

Supplementary Figure 1. Survival by age. Kaplan-Meier plots of the proportion of surviving patients over time in years. Patients were categorised by age. Vertical dashes represent right-censored patients. The number of patients remaining at risk at each time point are presented under the chart. Log-rank pair-wise analyses are presented above the corresponding chart **a.** Analyses including in-hospital deaths. **b.** Analyses excluding in-hospital deaths

A

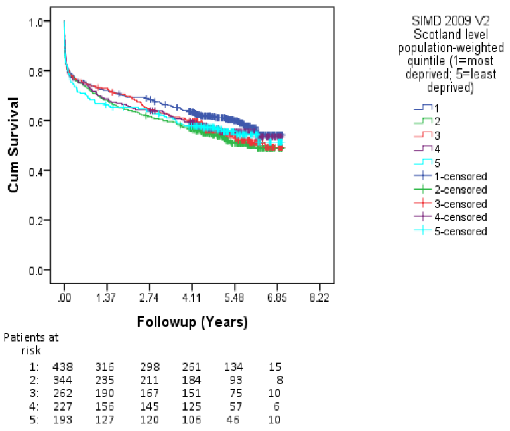

B

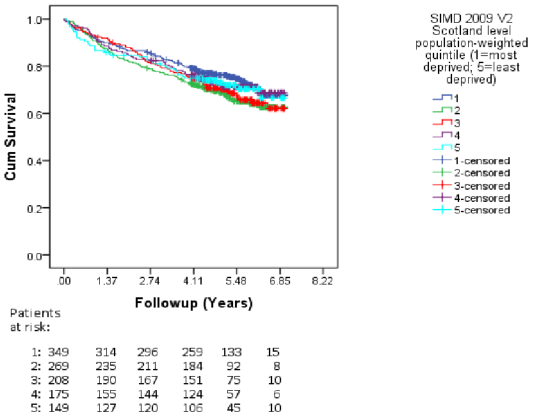

Pairwise Comparisons – including in hospital deaths

		1		2		3		4		5	
	SIMD 2009 V2 Scotland level population-weighted quintile (1= most deprived; 5=least deprived)	Chi-Square	Sig.	Chi-Square	Sig.	Chi-Square	Sig.	Chi-Square	Sig.	Chi-Square	Sig.
Log Rank (Mantel-Cox)	1			3.634	.057	1.801	.180	.248	.618	.456	.499
	2	3.634	.057			.175	.676	1.207	.272	.695	.405
	3	1.801	.180	.175	.676			.447	.504	.229	.632
	4	.248	.618	1.207	.272	.447	.504			.030	.864
	5	.456	.499	.695	.405	.229	.632	.030	.864		

Pairwise Comparisons – excluding in hospital deaths

		1		2		3		4		5	
	SIMD 2009 V2 Scotland level population-weighted quintile (1 = most deprived; 5 = least deprived)	Chi-Square	Sig.	Chi-Square	Sig.	Chi-Square	Sig.	Chi-Square	Sig.	Chi-Square	Sig.
Log Rank (Mantel-Cox)	1			3.473	.062	1.339	.247	.602	.438	1.130	.288
	2	3.473	.062			.293	.588	.643	.423	.205	.650
	3	1.339	.247	.293	.588			.075	.784	.000	.993
	4	.602	.438	.643	.423	.075	.784			.075	.785
	5	1.130	.288	.205	.650	.000	.993	.075	.785		

Supplementary Figure 2. Survival by Scottish Index of Multiple Deprivation (SIMD) quintile. Kaplan-Meier plots of the proportion of surviving patients over time in years. Patients were categorised by SIMD quintile. Vertical dashes represent right-censored patients. The number of patients remaining at risk at each time point are presented under the chart. Log-rank pair-wise analyses are presented above the corresponding chart **a.** Analyses including in-hospital deaths. **b.** Analyses excluding in-hospital deaths

A

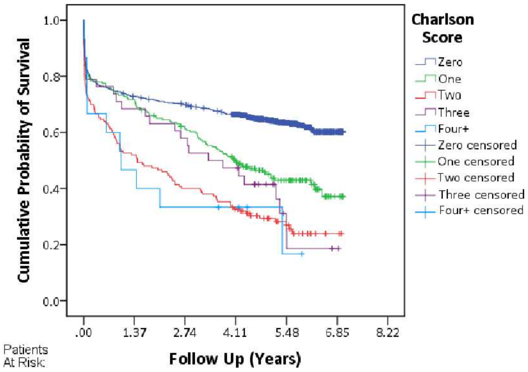

Patients At Risk:

Zero:	1055	766	732	663	328	39
One:	209	150	126	98	52	6
Two:	150	78	60	47	20	3
Three:	38	26	22	18	5	1
Four+:	15	7	5	4	1	0

B

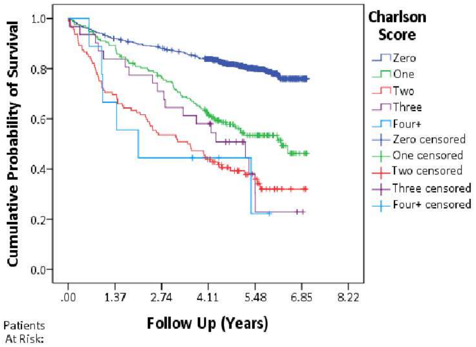

Patients At Risk:

Zero:	833	764	730	661	328	39
One:	168	150	126	98	52	6
Two:	112	78	60	47	20	3
Three:	31	26	22	18	5	1
Four+:	9	6	4	3	1	0

Pairwise Comparisons – including in-hospital deaths

	Charlson score	0		1		2		3		4+	
		Chi-Square	Sig.	Chi-Square	Sig.	Chi-Square	Sig.	Chi-Square	Sig.	Chi-Square	Sig.
Log Rank (Mantel-Cox)	0			22.485	.000	74.261	.000	12.801	.000	9.426	.002
	1	22.485	.000			13.189	.000	1.166	.280	3.167	.075
	2	74.261	.000	13.189	.000			1.147	.284	.038	.846
	3	12.801	.000	1.166	.280	1.147	.284			.782	.376
	4+	9.426	.002	3.167	.075	.038	.846	.782	.376		

Pairwise Comparisons – excluding in hospital deaths

		0		1		2		3		4+	
	Charlson score	Chi-Square	Sig.	Chi-Square	Sig.	Chi-Square	Sig.	Chi-Square	Sig.	Chi-Square	Sig.
Log Rank (Mantel-Cox)	0			52.398	.000	135.915	.000	35.007	.000	19.238	.000
	1	52.398	.000			12.621	.000	2.097	.148	3.383	.066
	2	135.915	.000	12.621	.000			.495	.482	.067	.796
	3	35.007	.000	2.097	.148	.495	.482			.441	.507
	4+	19.238	.000	3.383	.066	.067	.796	.441	.507		

Supplementary Figure 3. Survival by Charlson Score. Kaplan-Meier plots of the proportion of surviving patients over time in years. Patients were categorised by Charlson score. Vertical dashes represent right-censored patients. The number of patients remaining at risk at each time point are presented under the chart. Log-rank pair-wise analyses are presented above the corresponding chart **a.** Analyses including in-hospital deaths. **b.** Analyses excluding in-hospital deaths

A

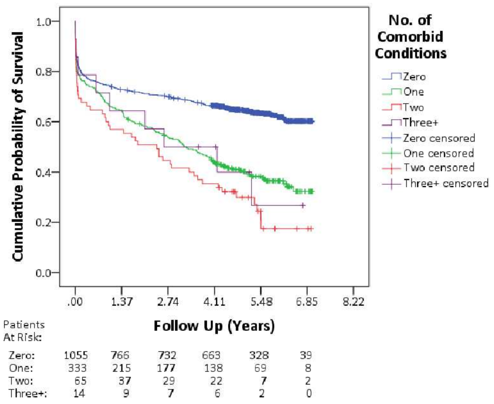

B

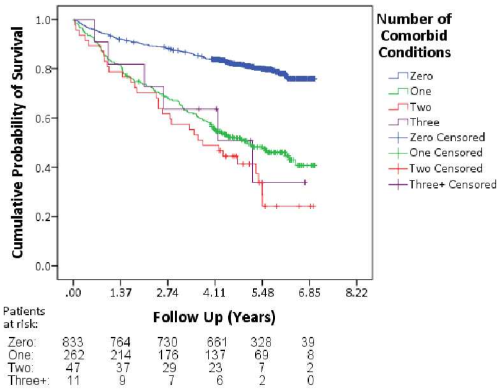

Pairwise Comparisons – including in hospital deaths									
		0		1		2		3+	
	Charlson No. of Conditios	Chi-Square	Sig.	Chi-Square	Sig.	Chi-Square	Sig.	Chi-Square	Sig.
Log Rank (Mantel-Cox)	0			57.146	.000	40.233	.000	3.749	.053
	1	57.146	.000			4.247	.039	.002	.968
	2	40.233	.000	4.247	.039			.607	.436
	3+	3.749	.053	.002	.968	.607	.436		

Pairwise Comparisons – excluding in hospital deaths									
	Charlson No. of Conditions	.00		1		2		3+	
		Chi-Square	Sig.	Chi-Square	Sig.	Chi-Square	Sig.	Chi-Square	Sig.
Log Rank (Mantel-Cox)	.00			113.399	.000	69.509	.000	9.289	.002
	1	113.399	.000			2.654	.103	.003	.954
	2	69.509	.000	2.654	.103			.324	.569
	3+	9.289	.002	.003	.954	.324	.569		

Supplementary Figure 4. Survival by number of conditions contributing to the Charlson Score. Kaplan-Meier plots of the proportion of surviving patients over time in years. Patients were categorised by the number of conditions contributing to their Charlson score. Vertical dashes represent right-censored patients. The number of patients remaining at risk at each time point are presented under the chart. Log-rank pair-wise analyses are presented above the corresponding chart **a.** Analyses including in-hospital deaths. **b.** Analyses excluding in-hospital deaths

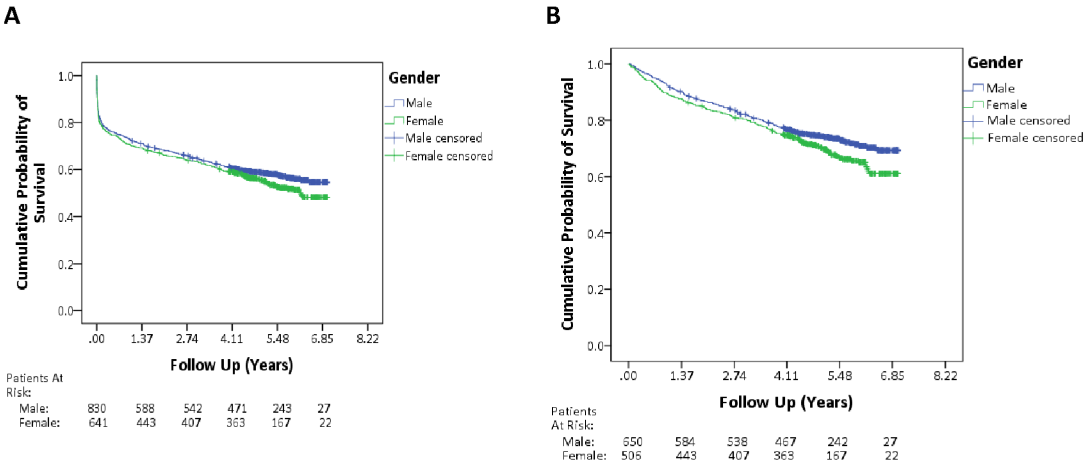

Supplementary Figure 5. Gender. Kaplan-Meier plots of the proportion of surviving patients over time in years. Patients were categorised by gender. Vertical dashes represent right-censored patients. The number of patients remaining at risk at each time point are presented under the chart. Log-rank analyses are presented to the right of the corresponding chart **a.** Analyses including in-hospital deaths (Log Rank test, P = 0.134). **b.** Analyses excluding in-hospital deaths (Log Rank test, P = 0.049)

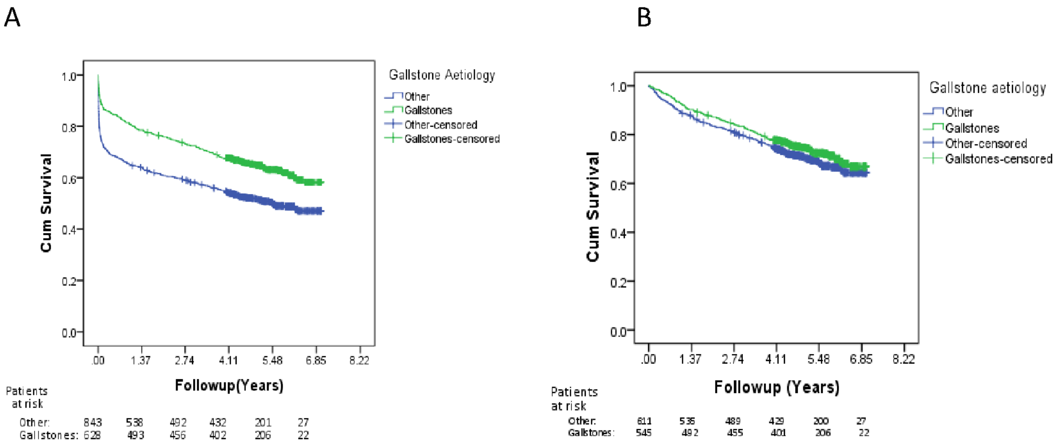

Supplementary Figure 6. Survival by aetiology of pancreatitis. Kaplan-Meier plots of the proportion of surviving patients over time in years. Patients were categorised by aetiology – gallstones or other causes. Vertical dashes represent right-censored patients. The number of patients remaining at risk at each time point are presented under the chart. Log-rank analyses are presented to the right of the corresponding chart **a.** Analyses including in-hospital deaths. (Log Rank test, Gallstones vs Other, P<0.001) **b.** Analyses excluding in-hospital deaths (Log Rank test, Gallstones vs Other, P=0.139)

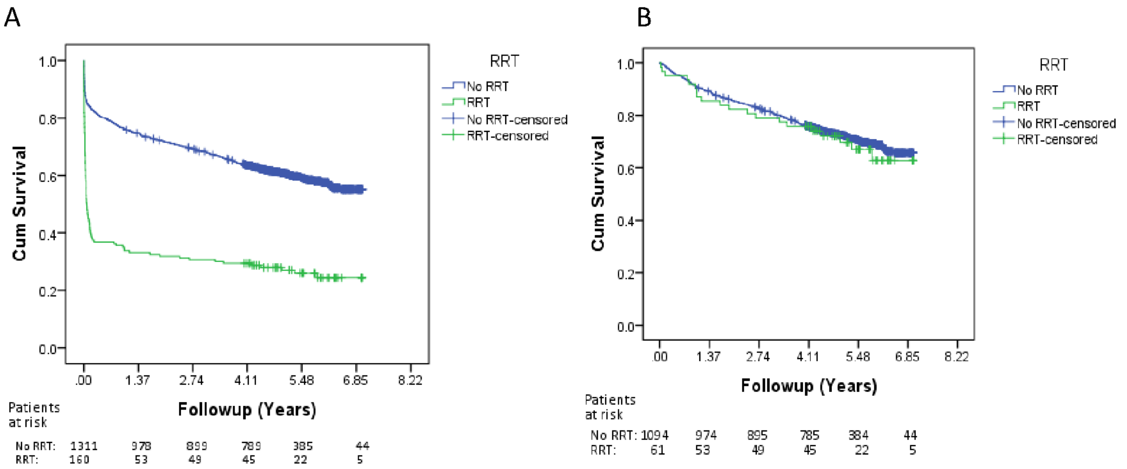

Supplementary Figure 7. Survival comparing use of renal replacement therapy (RRT). Kaplan-Meier plots of the proportion of surviving patients over time in years. Patients were categorised by whether they required RRT during the index admission. Vertical dashes represent right-censored patients. The number of patients remaining at risk at each time point are presented under the chart. Log-rank analyses are presented to the right of the corresponding chart **a.** Analyses including in-hospital deaths (Log Rank test RRT vs no RRT, P <0.001). **b.** Analyses excluding in-hospital deaths (Log Rank test RRT vs no RRT, P = 0.634).

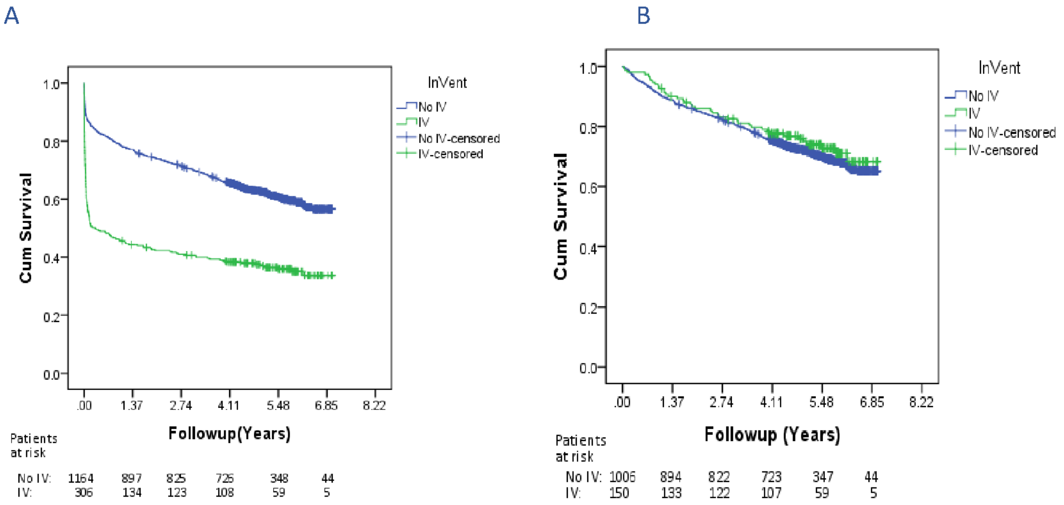

Supplementary Figure 8. Survival according to use of invasive ventilation. Kaplan-Meier plots of the proportion of surviving patients over time in years. Patients were categorised by whether they required invasive ventilation during the index admission. Vertical dashes represent right-censored patients. The number of patients remaining at risk at each time point are presented under the chart. Log-rank analyses are presented to the right of the corresponding chart **a.** Analysis including in-hospital deaths (Log Rank test inv. vent vs no inv. vent, P < 0.001). **b.** Analysis excluding in-hospital deaths (Log Rank test inv. vent vs no inv. vent, P = 0.428).

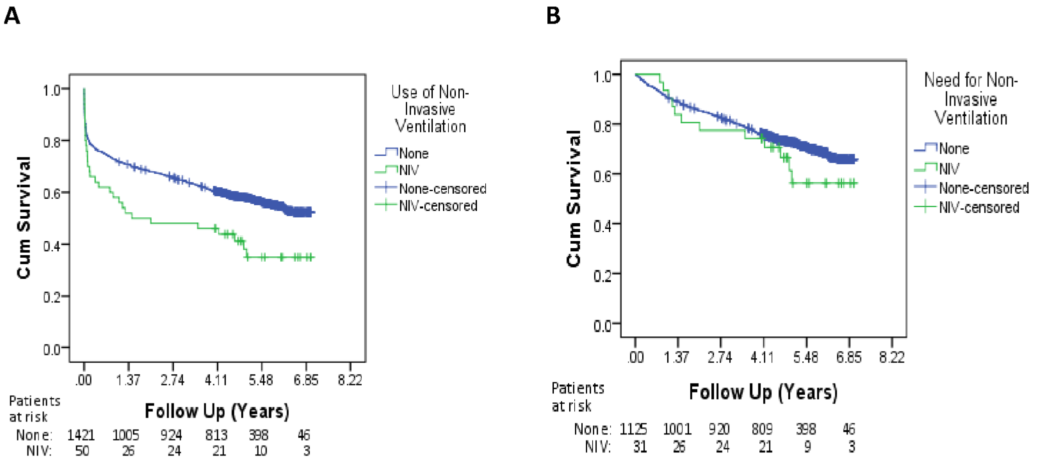

Supplementary Figure 9. Use of non-invasive ventilation (NIV). Kaplan-Meier plots of the proportion of surviving patients over time in years. Patients were categorised by whether or not they required NIV during the index admission. Vertical dashes represent right-censored patients. The number of patients remaining at risk at each time point are presented under the chart. Log-rank analyses are presented to the right of the corresponding chart **a.** Analyses including in-hospital deaths (Log Rank test NIV vs no NIV, P = 0.008). **b.** Analysis excluding in-hospital deaths (Log Rank test NIV vs no NIV, P = 0.301)

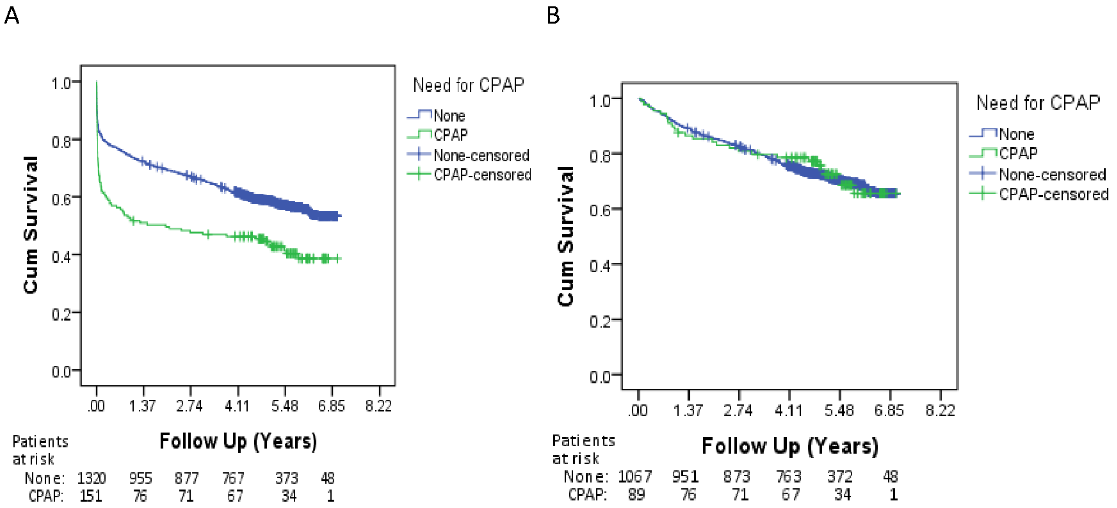

Supplementary Figure 10. Survival according to use of continuous positive airway pressure (CPAP). Kaplan-Meier plots of the proportion of surviving patients over time in years. Patients were categorised by whether they required CPAP during the index admission. Vertical dashes represent right-censored patients. The number of patients remaining at risk at each time point are presented under the chart. Log-rank analyses are presented to the right of the corresponding chart **a.** Analyses including in-hospital deaths (Log Rank test CPAP vs no CPAP, P < 0.001). **b.** Analysis excluding in-hospital deaths (Log Rank test CPAP vs no CPAP, P = 0.930)

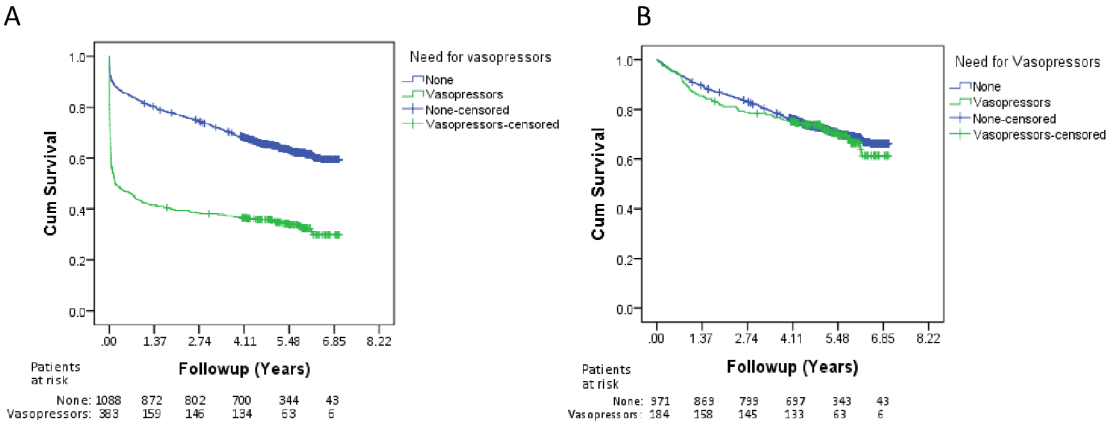

Supplementary Figure 11. Survival according to use of vasopressors. Kaplan-Meier plots of the proportion of surviving patients over time in years. Patients were categorised by whether they required vasopressors during the index admission. Vertical dashes represent right-censored patients. The number of patients remaining at risk at each time point are presented under the chart. Log-rank analyses are presented above the corresponding chart **a.** Analyses including in-hospital deaths (Log Rank test Vasopressors vs no Vasopressors, $P < 0.001$). **b.** Analysis excluding in-hospital deaths (Log Rank test Vasopressors vs no Vasopressors, $P = 0.579$)

Reporting checklist for cohort study.

Based on the STROBE cohort guidelines.

Instructions to authors

Complete this checklist by entering the page numbers from your manuscript where readers will find each of the items listed below.

Your article may not currently address all the items on the checklist. Please modify your text to include the missing information. If you are certain that an item does not apply, please write "n/a" and provide a short explanation.

Upload your completed checklist as an extra file when you submit to a journal.

In your methods section, say that you used the STROBE cohort reporting guidelines, and cite them as:

von Elm E, Altman DG, Egger M, Pocock SJ, Gotzsche PC, Vandenbroucke JP. The Strengthening the Reporting of Observational Studies in Epidemiology (STROBE) Statement: guidelines for reporting observational studies.

		Reporting Item	Page Number
Title	#1a	Indicate the study’s design with a commonly used term in the title or the abstract	1
Abstract	#1b	Provide in the abstract an informative and balanced summary of what was done and what was found	2
Background / rationale	#2	Explain the scientific background and rationale for the investigation being reported	4
Objectives	#3	State specific objectives, including any prespecified hypotheses	4
Study design	#4	Present key elements of study design early in the paper	5
Setting	#5	Describe the setting, locations, and relevant dates, including periods of recruitment, exposure, follow-up, and data collection	5
Eligibility criteria	#6a	Give the eligibility criteria, and the sources and methods of selection of participants. Describe methods of follow-up.	5

For peer review only

	#6b	For matched studies, give matching criteria and number of exposed and unexposed	6
Variables	#7	Clearly define all outcomes, exposures, predictors, potential confounders, and effect modifiers. Give diagnostic criteria, if applicable	5
Data sources / measurement	#8	For each variable of interest give sources of data and details of methods of assessment (measurement). Describe comparability of assessment methods if there is more than one group. Give information separately for for exposed and unexposed groups if applicable.	5
Bias	#9	Describe any efforts to address potential sources of bias	5
Study size	#10	Explain how the study size was arrived at	5
Quantitative variables	#11	Explain how quantitative variables were handled in the analyses. If applicable, describe which groupings were chosen, and why	6
Statistical methods	#12a	Describe all statistical methods, including those used to control for confounding	6
	#12b	Describe any methods used to examine subgroups and interactions	6
	#12c	Explain how missing data were addressed	5
	#12d	If applicable, explain how loss to follow-up was addressed	5
	#12e	Describe any sensitivity analyses	n/a
Participants	#13a	Report numbers of individuals at each stage of study—eg numbers potentially eligible, examined for eligibility, confirmed eligible, included in the study, completing follow-up, and analysed. Give information separately for for exposed and unexposed groups if applicable.	7
	#13b	Give reasons for non-participation at each stage	7
	#13c	Consider use of a flow diagram	7
Descriptive data	#14a	Give characteristics of study participants (eg demographic, clinical, social) and information on exposures and potential	18

For peer review only

		confounders. Give information separately for exposed and unexposed groups if applicable.	
	#14b	Indicate number of participants with missing data for each variable of interest	7
	#14c	Summarise follow-up time (eg, average and total amount)	7
Outcome data	#15	Report numbers of outcome events or summary measures over time. Give information separately for exposed and unexposed groups if applicable.	7
Main results	#16a	Give unadjusted estimates and, if applicable, confounder-adjusted estimates and their precision (eg, 95% confidence interval). Make clear which confounders were adjusted for and why they were included	19
	#16b	Report category boundaries when continuous variables were categorized	n/a
	#16c	If relevant, consider translating estimates of relative risk into absolute risk for a meaningful time period	8
Other analyses	#17	Report other analyses done—e.g., analyses of subgroups and interactions, and sensitivity analyses	8
Key results	#18	Summarise key results with reference to study objectives	9
Limitations	#19	Discuss limitations of the study, taking into account sources of potential bias or imprecision. Discuss both direction and magnitude of any potential bias.	10
Interpretation	#20	Give a cautious overall interpretation considering objectives, limitations, multiplicity of analyses, results from similar studies, and other relevant evidence.	11
Generalisability	#21	Discuss the generalisability (external validity) of the study results	10
Funding	#22	Give the source of funding and the role of the funders for the present study and, if applicable, for the original study on which the present article is based	1

The STROBE checklist is distributed under the terms of the Creative Commons Attribution License CC-BY. This checklist was completed on 18. April 2018 using http://www.goodreports.org/, a tool made by the EQUATOR Network in collaboration with Penelope.ai

For peer review only

BMJ Open

Survival and new-onset morbidity after critical care admission for acute pancreatitis in Scotland: a national electronic healthcare record linkage cohort study.

Journal:	*BMJ Open*
Manuscript ID	bmjopen-2018-023853.R2
Article Type:	Research
Date Submitted by the Author:	05-Nov-2018
Complete List of Authors:	Ventre, Chiara; The University of Edinburgh,<br>Nowell, Sian; NHS Scotland National Services Division<br>Graham, Catriona<br>Kidd, Doug; NHS Scotland National Services Division<br>Skouras, Christos; The University of Edinburgh,<br>Mole, Damian; Royal Infirmary of Edinburgh, General Surgery Department; The University of Edinburgh,
**Primary Subject Heading**:	Intensive care
Secondary Subject Heading:	Epidemiology, Gastroenterology and hepatology, Surgery, Diabetes and endocrinology
Keywords:	acute pancreatitis, multiple organ failure, long-term outcomes

SCHOLARONE™
Manuscripts

Title:

Survival and new-onset morbidity after critical care admission for acute pancreatitis in Scotland: a national electronic healthcare record linkage cohort study.

Authors: Chiara Ventre[1], Sian Nowell[2], Catriona Graham[3], Doug Kidd[2], Christos Skouras[1] and Damian J. Mole[1,4]

Institutions:

1. Surgery, School of Medicine, University of Edinburgh.
2. Electronic Data Research and Innovation Service (eDRIS), NHS National Services Scotland
3. Edinburgh Clinical Trials Unit, University of Edinburgh.
4. MRC Centre for Inflammation Research, University of Edinburgh

Corresponding author:

Professor Damian J Mole, MRC Senior Clinical Fellow, MRC Centre for Inflammation Research, 47 Little France Crescent, Edinburgh EH16 4SA.

damian.mole@ed.ac.uk

Funding Statement. This study did not receive specific funding. Data retrieval costs were covered by existing research funds. DJM currently holds an Medical Research Council Senior Clinical Fellowship MR/P008887/1.

Original Study Protocol. Not applicable

Competing Interests: None declared.

Data sharing statement for supplementary data: Original data are available on request through the NHS National Services Division Safe Haven, subject to an approval process. Further details may be found at http://www.isdscotland.org/Products-and-Services/eDRIS. Please contact the corresponding author in the first instance.

For peer review only - http://bmjopen.bmj.com/site/about/guidelines.xhtml

Keywords: acute pancreatitis; long-term outcomes; multiple organ failure

For peer review only

For peer review only - http://bmjopen.bmj.com/site/about/guidelines.xhtml

Abstract:

Introduction: Severe acute pancreatitis (AP) requiring critical care admission (ccAP) impacts negatively on long-term survival.

Objective: To document organ-specific new morbidity and identify risk factors associated with premature mortality after an episode of ccAP.

Design: Cohort study

Setting: Electronic healthcare registries in Scotland.

Participants: ccAP cohort: 1471 patients admitted to critical care with AP between 1st January 2008 and 31st December 2010 followed up until 31st December 2014; population cohort: 3450 individuals from the general population of Scotland frequency matched for age, sex and social deprivation.

Methods: Record linkage of routinely-collected electronic health data with population matching.

Primary and secondary outcome measures: Patient demographics, co-morbidity (Charlson Index), acute physiology, organ support and other critical care data were linked to records of mortality (death certificate data) and new-onset morbidity. Kaplan Meier and Cox regression analyses were used to identify risk factors associated with mortality.

Results: 310 patients with AP died during the index admission. Outcomes were not ascertained for 5 patients, and the deprivation quintile was not known for 6 patients. 340 of 1150 patients in the resulting post-discharge ccAP cohort died during the follow-up period. Greater co-morbidity measured by the Charlson score, prior to ccAP, negatively influenced survival in hospital and after discharge. The odds of developing new-onset diabetes mellitus after ccAP compared to the general population was 10.70 (95% C.I. 5.74 to 19.94). A new diagnosis of myocardial infarction, stroke, heart failure, liver disease, peptic ulcer, renal failure, cancer, peripheral vascular disease and lung disease was more frequent in the ccAP cohort than the general population.

Conclusions: The persistent deleterious impact of severe AP on long-term outcome and survival is multifactorial in origin, influenced by pre-existing patient characteristics and acute episode features. Further mechanistic and epidemiologic investigation is warranted.

Abstract Word Count: 288 words

For peer review only - http://bmjopen.bmj.com/site/about/guidelines.xhtml

Article summary

Strengths and limitations of this study

1. This study is in a large contemporary cohort of patient with AP covering a national population (Scotland).
2. Through secure record linkage, post-ccAP morbidity data are analysed in context of episode specific and pre-existing morbidity data
3. The use of pre-existing national databases resulted in low, but not negligible, amounts of missing data. The amount of missing data might have been further reduced had it been possible to prospectively capture all primary data.
4. Only gallstone aetiology could be specifically examined due to data inaccuracies in the recording of other aetiologies of acute pancreatitis, specifically alcohol excess.
5. The analysis of existing and new comorbidities was limited by the relatively small proportion of patients affected by each comorbidity, and, because co-morbidities derived from SMR01 data only reveal diagnoses made at the time of a hospital admission and therefore are an underestimate of the true population prevalence.

Author contributions. DJM, CS and CV conceived the study. Data retrieval, linkage and secure storage was done by DK and SN. Statistical design and analysis was done by SN and CG. CV and DJM drafted the initial version of the manuscript. All authors revised and approved the final version of the paper. DJM is guarantor.

For peer review only

For peer review only - http://bmjopen.bmj.com/site/about/guidelines.xhtml

Introduction

Acute pancreatitis (AP) is the most common gastrointestinal cause of emergency hospital admission. The incidence of AP is increasing, and in Scotland is 31.8 per 100,000[1-5]. Overall case fatality in AP is 5%[5]. Although most cases are mild and self-limiting, 1 in 4 patients with AP develop multiple organ dysfunction syndrome (AP-MODS) and require critical care admission[6]. AP-MODS is the single most important determinant of death from AP[7], with mortality in patients with AP-MODS reaching 21.7% [5]. Recently, we reported that AP-MODS has detrimental consequences even for those who survive the acute episode, who have a reduced overall survival compared to AP without MODS[8]. Prevention of AP-MODS in humans remains an elusive goal[9], and it is therefore important to characterise the lasting impact on survivors to help maximise their long-term well-being.

AP has many potential causes, of which gallstones and alcohol are most frequently implicated[6 10]. The resulting inflammatory reaction within the pancreas may become over-amplified and precipitate a systemic inflammatory response, shock and organ dysfunction[6 10-13]. There is marked inter-individual heterogeneity in the number of organ systems involved and AP-MODS can affect any organ system, with the respiratory and renal systems most frequently affected [14-17]. Moreover, the severity of organ dysfunction is highly variable, and interventions including invasive ventilation and renal replacement therapy can be required for durations raging from 1 day to 10 weeks[18 19]. AP-MODS determines mortality during the index admission[20] but it is not certain which organ-specific failures are particularly associated with deterioration to death. One study linked hepatic and renal failures with the highest mortality risk[19], whereas another placed greater negative influence after failure of the cardiovascular, pulmonary and gastrointestinal systems[16].

Importantly, it is not completely understood which specific organ deficits may persist in survivors of AP-MODS. AP-MODS has been associated with an increased incidence of diabetes in AP survivors[15 21 22], and age and working status are important in predicting recovery of quality of life and functional capacity[21]. Moreover, given the heterogeneity of the course of AP-MODS, it is unclear if a subgroup of AP-MODS survivors is at particularly high risk of a poor outcome. In the absence of an intervention to prevent AP-MODS, a deeper understanding of the persistent pathophysiological impact left by AP-MODS is needed. Therefore, our aim in this study was to integrate routinely-collected data to investigate the causes and predictors of mortality in the years following an episode of AP requiring critical care admission.

For peer review only - http://bmjopen.bmj.com/site/about/guidelines.xhtml

Methods

Study Design, Data Security and Patient Confidentiality

This retrospective cohort study was conducted in collaboration with eDRIS to facilitate record linkage from multiple national databases. Approval was obtained from the Privacy Advisory Committee of Information Services Division (ISD) Scotland. Information governance and security protocols were adhered throughout the investigation. All primary data was stored securely. Research ethical committee review was not required for this study after consulting the guidance applicable to Scotland publicly available from the United Kingdom NHS Health Research Authority. Individual informed consent was not required or sought for this study.

Patient and Public involvement

We work closely with our patient and public involvement group, APPLe (Acute Pancreatitis Patient Liaison), to develop our research projects and strategies. This study received general input from members of APPLe as part of a consortium building workshop for the APPreSci Consortium (Acute Pancreatitis Precision Science, www.appresci.com), but APPLe was not involved in the data collection, analysis or manuscript preparation.

Patient Identification & Data Collection

All data were handled according to the Charter for Safe Havens in Scotland[23]. The Scottish Intensive Care Society Audit Group (SICSAG) WardWatcher database[24] was used to identify all patients admitted to critical care with AP between 1st January 2008 and 31st December 2010. AP was defined as any admission to critical care where the primary diagnosis coded by the intensive care senior clinician on duty was recorded as ICD-10 classification K85 (acute pancreatitis). Where an individual was admitted with AP on more than one occasion, the earliest AP episode was taken as the index episode. There were no additional exclusion criteria. We performed a record-linkage analysis of Scottish Morbidity Record (SMR) 01 (General Acute Inpatient and Day Cases), General Register Office (GRO) death records, SICSAG (critical care) and Community Health Index (CHI) databases. General population of Scotland causes of death were obtained from National Records of Scotland Vital Events Tables[25]. Patient outcomes were recorded from the date of their index admission until the end of the follow-up period on 31st December 2014. Those lost to follow up were censored at the point of last known contact. Prior to analysis, data records were linked using unique patient identifiers in order to maintain confidentiality.

For peer review only - http://bmjopen.bmj.com/site/about/guidelines.xhtml

Variables of Interest

The primary outcome of interest was death. The secondary outcomes were cause of death and new-onset morbidity. The following details of the index AP episode were recorded for each patient: gallstone aetiology (from SMR01 data), APACHE II score (acute physiology and chronic health evaluation score, version 2), length of stay in critical care, level of critical care admission (high-dependency unit (HDU) or intensive care unit (ICU))[26], and the requirement for renal replacement therapy, invasive ventilation, non-invasive ventilation, continuous positive airways pressure (CPAP) or vasopressor support (all from SICSAG data). In addition, the following patient characteristics were recorded: age on admission (from SICSAG), gender (from the CHI database), Scottish Index of Multiple Deprivation (SIMD), Charlson score for comorbidity (calculated from SMR01 records for each patient in the 5 years prior to admission)[27] and the number of comorbid conditions contributing to the Charlson score. The cause of death was obtained for each deceased patient and sorted according to ICD-10 code into one of five categories: Cardiovascular/Circulatory, Respiratory, Neoplasia, Digestive/Metabolic, or Other Causes as shown in **Supplementary Table 1**.

Statistical Analysis

IBM SPSS Statistics Version 21 (IBM Corp., Armonk, New York) was used for all analyses. Categorical variables were reported as the absolute frequency and percentage. Continuous variables were reported as the mean ± standard deviation (SD) or the median ± interquartile range (IQR). Kaplan-Meier analysis was used to demonstrate survival with respect to demographic and clinical factors, with the significance of differences assessed using the log-rank (Mantel-Cox) test. Survival was calculated as the time from the index admission to hospital with AP to death; analyses and plots were done for the whole cohort, and for the cohort excluding those members who died during the index episode of AP in order to allow for analysis of long-term outcomes in survivors of the index episode, as specified in the Results and Figures.

The proportional hazards assumption was tested using log(-log) plots of the survival function over time, to confirm that the curves were approximately parallel. A multivariate Cox regression model was constructed to account for potential interactions between predictor variables. Covariates were added to the model using a forward stepwise method. At each step, the covariate found to be most significant was retained in the model. The threshold for retention in models created using SPSS was p=0.01. After each addition, the covariates already present in the model were tested for removal depending on the significance of the likelihood ratio with and without each covariate.

A P value of 0.05 or less was considered significant. Where multiple pairwise comparisons were made – age group (< 20, 20 to 29, 30 to 39, 40 to 49, 50 to 59, 60 to 69, 70-79, > 80 years), Charlson score (0, 1, 2,

For peer review only - http://bmjopen.bmj.com/site/about/guidelines.xhtml

3, 4+), number of comorbid conditions (0, 1, 2, 3+) – the Bonferroni correction was applied to account for the quantity of comparisons being made.

Secondary Analysis of Associated Comorbidities

A control group was created from the general population using the CHI database register of all patients in Scotland. Controls were frequency matched on deprivation quintile, age (by year of birth) and sex. Three controls were selected for every member of the exposed group. Comorbidities at index admission for AP were obtained from the SMR01 computerised acute hospital discharge records (day cases or inpatients) in the 5-year look back period from date of admission for the index AP episode. Comorbidities that developed after discharge were ascertained from admissions after the index admission for AP up to 31st December 2014. The comorbidities that developed after the index event were then compared in the exposed and unexposed groups with a two-sample z test. We calculated the odds ratio (and 95% C.I) of developing each comorbidity given previous admission for AP needing critical care, compared to people with no previous AP admission.

Results

Follow-up and Survival

Between 1st January 2008 and 31st December 2010, 1471 patients were admitted to HDU or ICU with AP. The length of the follow-up period ranged from 4.0 to 7.0 years. The median duration of follow-up from the date of index admission for all AP patients was 4.4 years (IQR 0.6 to 5.6 years) and 4.9 years (IQR 4.0 to 5.8 years) when patients who died in hospital during the index admission were excluded. 16 patients moved to another country and were censored at the point of last known contact. **Figure 1** outlines the demographics of the study population. Demographic data for the cohort are presented in **Table 1**.

		Died during index admission	(% of total)	Survived index admission	(% of total)	Died after hospital discharge	(% of total)
**Gender**							
	**Male**	175	21	475	58	175	21
	**Female**	135	21	341	53	165	26
**Age group**							
	**<20**	0	0	19	95	1	5
	**20-29**	7	9	66	84	6	8
	**30-39**	13	9	112	78	19	13
	**40-49**	22	11	141	72	34	17
	**50-59**	47	19	157	62	49	19

For peer review only - http://bmjopen.bmj.com/site/about/guidelines.xhtml

**60-69**	72	25	143	49	75	26
**70-79**	83	27	135	44	86	28
**80+**	66	37	43	24	70	39
**Scottish Index of Multiple Deprivation (SIMD) Quintile**						
**1 – most deprived**	86	20	257	59	92	21
**2**	75	22	179	52	90	26
**3**	54	21	141	54	67	26
**4**	51	23	126	56	49	22
**5 – least deprived**	44	23	107	55	42	22
**Not known**	0	0	6	100	0	0
**Charlson comorbidity index**						
**0**	219	21	666	63	167	16
**1**	41	20	91	44	77	37
**2**	38	25	41	27	71	47
**3**	7	18	12	32	19	50
**4+**	5	36	3	21	6	43
**Not known**	0	0	3	100	0	0
**Number of comorbid conditions contributing to the Charlson Index**						
**0**	219	21	666	63	167	16
**1**	70	21	126	38	136	41
**2**	18	28	16	25	31	48
**3+**	3	21	5	36	6	43
**Not known**	0	0	3	100	0	0

Table 1. Demographic characteristics of the ccAP cohort. The absolute number of patients and row percentages per each category for the following variables of interest are presented: gender, age group, Scottish Index of Multiple Deprivation (SIMD)[28], Charlson co-morbidity index[27], and number of co-morbid conditions contributing to the Charlson index.

For peer review only

During the follow-up period 651 of 1471 (44.3%) patients died. 310 of 651 (47.8%) deaths occurred during the index admission, the outcome of 5 was not known and the deprivation quintile for 6 other patients was not known. The post-discharge critical care AP (ccAP) cohort therefore included 1150 patients, of which 340 died during the follow-up period. As over half of the study cohort survived to the end of follow-up, the median survival time could not be determined. The mean (± standard deviation) survival time in the whole cohort was 4.4 ± 0.1 years and 5.6 ± 0.1 years once in-hospital deaths were excluded.

Cause of death

In the post-discharge ccAP cohort, neoplasms were the leading cause of death (27.9%), followed by cardiovascular (27.1%) and digestive/metabolic deaths (25.9%) **(Figure 2).** Other causes contributed only

For peer review only - http://bmjopen.bmj.com/site/about/guidelines.xhtml

5.3% of the total. This contrasted with the general population of Scotland for which a lower proportion of deaths was attributed to digestive causes (7.3%) while a markedly greater proportion of the general population controls were due to other causes (21.0%) (**Figure 2**).

Predictors of mortality in the post-discharge cohort

Independent negative risk factors for long term survival included age **(Suppl. Fig. 1),** a Charlson score of 1 or greater (**Table 2** and **Figure 3a**), and the number of comorbid conditions contributing to the Charlson score (**Table 2** and **Figure 3b**). Survival did not differ significantly with the degree of social deprivation **(Suppl. Fig 2).** Female gender was associated with a shorter survival on univariate analysis, but gender as a risk factor on the multivariate analysis was not significant (**Table 3** and **Figure 3c**). Gallstone aetiology was associated with a lower mortality after discharge (**Table 3**). Comparison with analyses that included in-hospital deaths indicated that these differences emerged post-discharge (**Suppl. Fig 1-6**). Multivariate Cox regression analysis also identified increased age group and Charlson comorbidity score as poor prognostic factors (**Table 3**).

For peer review only - http://bmjopen.bmj.com/site/about/guidelines.xhtml

Risk factor		*n*	Hazard Ratio	95% CI	p value
**Age**	**under 20**	20	–		
(reference <20 years)	**20 - 29**	72	1.6	0.2, 13.1	0.674
	**30 - 39**	131	2.9	0.4, 21.8	0.296
	**40 - 49**	175	3.9	0.5, 28.5	0.180
	**50 - 59**	206	5.0	0.7, 36.5	0.110
	**60 - 69**	218	7.9	1.1, 57.0	0.040
	**70 - 79**	221	8.7	1.2, 62.4	0.032
	**80+**	113	17.3	2.4, 124.4	0.005
**Gender**	**Male**	650	–		
(reference Male)	**Female**	506	1.2	1.0, 1.5	0.049
**Charlson Score**	**0**	833	–		
(reference 0)	**1**	168	2.6	2.0, 3.4	<0.001
	**2**	112	4.7	3.6, 6.2	<0.001
	**3**	31	3.8	2.4, 6.1	<0.001
	**4 or more**	9	5.3	2.4, 12.0	<0.001
**Number of comorbid conditions**	**0**	833	–		
(reference 0)	**1**	262	3.2	2.6, 4.0	<0.001
	**2**	47	4.5	3.0, 6.7	<0.001
	**3**	11	3.3	1.5, 7.5	0.004
**Length of stay**	**less than 20 days**	1079	–		
(reference <20 days)	**longer than 20 days**	77	0.4	0.2, 0.8	0.006
**Level of critical care**	**ICU**	251	–		
(reference ICU)	**HDU**	905	1.2	1.0, 1.4	0.019

Table 2. Predictors of long-term mortality - univariate regression analysis. The hazard ratio, 95% confidence intervals (CI) and p value of Wald's test are presented for each variable found to significantly affect post-discharge survival on univariate regression analysis. p<0.05 was considered significant. The reference category for each variable is appended. Age has been transformed to a categorical variable for the purposes of the analysis. ***n****: number of patients per category;* ***HDU****: High-Dependency Unit;* ***ICU****: Intensive Care Unit.*

For peer review only

For peer review only - http://bmjopen.bmj.com/site/about/guidelines.xhtml

Risk Factor	Hazard Ratio	95% CI	p value
**Age**	1.0	1.0, 1.1	<0.001
**Charlson score**	1.5	1.4, 1.7	<0.001
**Female gender**	1.2	1.0, 1.5	0.058
**Gallstone aetiology**	0.7	0.6, 0.9	0.003

Control variables not in the final model: renal replacement therapy, invasive ventilation, non-invasive ventilation, vasopressor use and SIMD (deprivation index)

*Age group as defined in Table 2

Table 3. Final model of prognostic factors of post-discharge mortality. The hazard ratio, 95% confidence intervals and p value of Wald's test are presented for each variable retained in the final multivariate Cox regression model. $p<0.05$ was considered significant.

A significant relationship was observed between the requirement for renal replacement therapy, respiratory or circulatory support and an increased risk of death when in-hospital deaths were included **(Suppl. Fig 7-11)**. However, no correlation between mortality and any of the aforementioned medical interventions, or gallstone aetiology, was observed when considering only post-discharge outcomes **(Suppl. Figs 6-11)**. Long-term survival of those who survived the index episode was significantly better where the length of stay in critical care during the index episode exceeded 20 days, compared to admissions of 0-4 or 10-19 days (**Figure 4a**). The critical care setting was important – patients with AP-MODS admitted to ICU, and who survived that event, had better long-term survival compared to those who were admitted to HDU and survived (**Figs 4b** and **4c,** and **Table 2**).

Development of new specific comorbidities

Patients in the ccAP cohort were significantly more likely to develop a range of cardiovascular, gastrointestinal, pulmonary and neoplastic conditions than matched controls (**Table 4**). A particularly high risk of developing new-onset diabetes was noted (OR 10.70, 95% C.I. 5.74 to 19.94), with 3.9% of the ccAP cohort developing new diabetes during the follow-up period compared to 0.4% of the matched control group. The risk of developing renal disease requiring hospital admission was also markedly increased (OR 9.15, 95% C.I. 2.95 to 28.43), but whether this was confounded by new or existing diabetes could not be ascertained, and the numbers of people affected by renal disease was small in both cohorts. Risks for developing other comorbidities are presented in **Table 4**.

For peer review only - http://bmjopen.bmj.com/site/about/guidelines.xhtml

	Co-morbidities at SICSAG admission			New co-morbidities developed after discharge					
Comorbidity	% of AP cohort	% of controls	P value	% of AP cohort	% of controls	P value	Odds Ratio[1]	95% CI Lower	95% CI Upper
AMI	2.7	1.2	<0.001	3.7	1.8	<0.001	2.09	1.4	3.12
cerebral vascular accident	1.9	1.7	0.555	3.5	2.2	0.021	1.58	1.07	2.35
Congestive heart failure	2.3	0.4	<0.001	2.5	0.8	<0.001	3.11	1.83	5.28
Connective tissue disorder	0.8	0.3	0.024	0.1	0.1	0.641	0.6	0.07	5.16
Diabetes & Diabetes complications	1.8	0.2	<0.001	3.9	0.4	<0.001	10.7	5.74	19.94
Liver disease & Severe Liver Disease	0.5	0.1	0.004	0.6	0.1	0.003	5.3	1.55	18.14
Peptic ulcer	1.9	0.3	<0.001	1.6	0.3	<0.001	5.06	2.38	10.74
Peripheral vascular disease	1.5	0.6	0.007	1.3	0.5	0.002	2.86	1.41	5.81
Pulmonary disease	3.8	1.1	<0.001	2.5	1.6	0.033	1.65	1.04	2.62
Cancer & Metastatic cancer	7.2	2.8	<0.001	8.4	5.4	<0.001	1.62	1.25	2.11
Renal disease	1	0.2	0.001	1.1	0.1	<0.001	9.15	2.95	28.43

Table 4. Baseline comorbid status and risk of developing new comorbidities after the index AP episode. The percentage of patients and controls who developed each specified comorbidity in the 5 years before and 5 years after the index AP episode are presented. The odds ratio, as well as the 95% confidence intervals for the development of each comorbidity after the AP episode are included. Total number of patients from the AP cohort: 1150, total number of controls: 3450. The p values were obtained by applying the 2-sample Z-test. $p<0.05$ was considered significant. ***SICSAG****: The Scottish Intensive Care Society Audit Group software;* ***AMI****: acute myocardial infarction.*

In addition to evaluating the risk of new onset co-morbidity, we examined whether the baseline comorbidities of the population who experience an episode of AP might be different to the general population (**Table 4**). At the time of presentation with their index episode, patients with AP needing critical care were significantly more likely to have existing co-morbidities that included cardiac, lung, renal or peripheral vascular disease, heart failure, connective tissue disorders, peptic ulcers and cancer than matched general population controls. ccAP therefore appears to be a feature associated with members of the population that are already less healthy.

For peer review only - http://bmjopen.bmj.com/site/about/guidelines.xhtml

Discussion

This retrospective data linkage cohort study aims to investigate the causes and predictors of mortality in the years following an episode of AP requiring critical care admission. In so doing, statistically significant differences in frequency of the causes of death have been demonstrated between the ccAP patient cohort and the general population. In addition, the results indicate that long-term prognosis after a critical care admission for AP is influenced to a greater extent by age at the time of index AP admission and existing comorbidity than by specific features of the index AP episode. New-onset comorbidity, particularly diabetes, is more frequent following ccAP than in the general population. We acknowledge that ccAP patients may have additional diagnoses made because these individuals seek more frequent contact with healthcare and therefore have the opportunity to get diagnosed with comorbidities. Furthermore, co-morbidities derived from SMR01 data only reveal diagnoses made at the time of a hospital admission and are therefore an underestimate of the true population prevalence. From our analysis it is not possible to discern whether those individuals were destined to develop those co-morbidities regardless of their episode of AP, especially given that the AP cohort is less healthy overall than the matched general population. A similar association between MODS and mortality has been demonstrated in patients who have sustained trauma[29]. Our results support our previously-observed concept that ccAP is associated with a persistent deleterious impact on survivors. Inter-individual heterogeneity in the clinical course of the AP critical care episode was not associated with any organ-specific long-term outcomes in this analysis, but we acknowledge that our approach was limited in the ability to discriminate these with certainty.

Together, these findings lend weight to the hypothesis that severe AP episodes do not fully resolve, with particular emphasis on the impact of the associated systemic dysfunction. Our study has added data and analysis to underpin this concept by investigating the specific details of the deleterious legacy of ccAP. Given that the variation in causes of death is largely due to an increased proportion of deaths from metabolic disease, it is reasonable to infer that AP mediates the long-term effects primarily through ongoing metabolic pathology. This result concurs with outcomes in a Danish cohort that demonstrated a marked increase in deaths from digestive system causes in AP survivors compared to the general population[30]. Exact mechanisms underpinning the metabolic disturbance remain to be elucidated and will almost certainly require a prospective experimental medicine study. Taken together, the reported high incidence of diabetes mellitus after AP, the correlation of AP severity with lasting pancreatic exocrine dysfunction (as shown by others), and the negative effect of exocrine pancreatic insufficiency, suggest that impairment of the endocrine and exocrine pancreas is the main driver of the lasting overall dysfunction[15 21 22]. Additionally, it is reasonable to expect that aspects of the acute systemic dysfunction associated with MODS, for example insulin resistance and mitochondrial dysfunction, fail to resolve entirely[31], although we have not tested this experimentally in this study.

For peer review only - http://bmjopen.bmj.com/site/about/guidelines.xhtml

Identifying predictors of post-discharge mortality will facilitate appropriate targeting of preventative interventions. The identification of greater pre-existing comorbidity as a key negative predictive factor is consistent with previous research correlating more extensive comorbid disease with a worse prognosis after critical illness[32]. In the present study, our observation that post-discharge outcomes were better for ICU than HDU patients by univariate analysis could be explained by comorbidity – those requiring ICU admission theoretically experience the worst AP episodes, and therefore only relatively fitter individuals may survive to discharge. In contrast, those with greater comorbidity, and hence a higher risk of later mortality, may survive an AP episode managed in HDU. We acknowledge that the preceding statement is somewhat speculative, despite being highly plausible. A similar argument may explain the association of better long-term outcomes with a critical care stay exceeding 20 days, in that individuals with less associated comorbidity at AP onset may be more resilient to a prolonged critical care admission. This finding is in contrast to data on long-term survival in all ICU patients, where prolonged admission was associated with a shorter long-term survival[33 34]. However, the positive association between duration of organ support in ICU and post-ccAP survival is likely subject to iatrogenic influences. For example, a willingness to persist with organ support in critical care by the physician-led multidisciplinary team in those without significant medical comorbidity prior to ICU admission, may result organ support being continued for longer, a form of survivor treatment selection bias[35].

We observed that gallstone aetiology had a less negative effect on prognosis. While this might be explained by the additional burden of morbidity and mortality carried by alcohol misuse, the other key cause of AP[36-38], our data implies that gallstone AP requiring critical care has less severe long-term consequences. This is in contrast to previous studies by others, where, in acute AP, a gallstone aetiology was associated with more severe MODS than alcohol-induced cases [39], and separately, no effect of gallstone aetiology on long-term prognosis after accounting for the detrimental impact of alcohol[30]. It is important to note that alcohol-related AP was not specifically known in our study population.

A strength of this study is that the applicability of these observations to all ccAP survivors has been enhanced by using primary data from a population basis rather than a single centre, in collaboration with Farr@Scotland. This UK-wide network was created to facilitate the storage, sharing and analysis of population and health-related datasets in an environment that protects patient confidentiality and data security[40]. The employment of this resource facilitated the achievement of larger sample population than would have been possible with a single-centre study and reduced the risk of the results being modified by, for example, regional variations in treatment or population demographics[41 42]. Matching each member of the ccAP cohort by year of birth, deprivation and sex to three individuals sampled from the remaining general population diminished any potential influence of national secular trends in the population incidence on the specific outcomes measured.

We acknowledge specific limitations of our study. Firstly, the use of pre-existing national databases requires an acceptance of low amounts of missing data that might have been avoided had it been possible

For peer review only - http://bmjopen.bmj.com/site/about/guidelines.xhtml

to prospectively capture all primary data. However, the expense and time needed to do that would make a study of this size extremely unwieldy, and we regard our approach to be preferable to that, at this stage. The incidence of missing data was very low, with the exception of APACHE II scores. In order to overcome data inaccuracies, only gallstone aetiology was specifically noted. Our experience of using these records to correctly attribute alcohol aetiology have been not sufficiently reliable as a foundation for a robust analysis. Though not possible within the limitations of the current study, this will be an important consideration in advancing this research. Because there was uncertainty in our attribution of ccAP aetiology, with the exception of those diagnosed with gallstones, coupled with our use of relatively healthy controls from the general population, we were unable to analyse future causes of death and survival bias based on that factor. Insufficient detail in this dataset precluded a robust analysis regarding the frequency of recurrent episodes of AP in the cohort, because it was not possible to distinguish repeat hospital admissions due to complications arising from the index episode from true *de novo* recurrent episodes. This would be addressed by a prospective study. Finally, the analysis of existing and new comorbidities was limited by relatively low proportions of patients affected by each comorbidity. The value of replicating these findings using larger patient cohorts would need to be weighed against the practical challenges but should be considered. Further clarification of this phenomenon, and the impact on other body systems, is in progress through a prospective experimental medicine cohort study[43]. The identification of specific goals for intervention in the follow-up period after AP will require that detailed assessment of alterations in patients' physiological status over time.

In conclusion, long-term outcomes after AP requiring critical care are influenced by pre-existing patient characteristics and specific factors associated with an episode of critical care admission. Persisting metabolic derangement after ccAP is associated with premature death. The persistent deleterious impact of severe AP on survival is multifactorial, and further mechanistic and epidemiologic investigation is required.

Acknowledgments

DJM acknowledges the support of the MRC through a Senior Clinical Fellowship. We are grateful to members of APPLe for their personal insight and advise on our overall programme of work.

For peer review only - http://bmjopen.bmj.com/site/about/guidelines.xhtml

References

1. O'Farrell A, Allwright S, Toomey D, et al. Hospital admission for acute pancreatitis in the Irish population, 1997–2004: could the increase be due to an increase in alcohol-related pancreatitis? *Journal of Public Health* 2007;29(4):398-404.
2. Fagenholz PJ, Castillo CF-D, Harris NS, et al. Increasing United States Hospital Admissions for Acute Pancreatitis, 1988–2003. *Annals of Epidemiology* 2007;17(7):491.e1-91.e8. doi: http://dx.doi.org/10.1016/j.annepidem.2007.02.002
3. Sandzén B, Rosenmüller M, Haapamäki MM, et al. First attack of acute pancreatitis in Sweden 1988 – 2003: incidence, aetiological classification, procedures and mortality – a register study. *BMC Gastroenterology* 2009;9:18-18. doi: 10.1186/1471-230X-9-18
4. McKay C, Evan S, Sinclair M, et al. High early mortality rate from acute pancreatitis in Scotland: 1984-1995. *Br J Surg* 1999;86(10):1302-05.
5. Mole D, Gungabissoon U, Johnston P, et al. Identifying risk factors for progression to critical care admission and death among individuals wih acute pancreatitis by utilizing linked Scottish healthcare databases. *Lancet* 2015
6. Hayden P, Wyncoll D. Severe acute pancreatitis. *Current Anaesthesia & Critical Care* 2008;19(1):1-7. doi: http://dx.doi.org/10.1016/j.cacc.2007.01.009
7. Andersson R, Andersson B, Haraldsen P, et al. Incidence, management and recurrence rate of acute pancreatitis. *Scandinavian Journal of Gastroenterology* 2004;39(9):891-94.
8. Skouras C, Hayes AJ, Williams L, et al. Early organ dysfunction affects long-term survival in acute pancreatitis patients. *HPB* 2014;16(9):789-96. doi: 10.1111/hpb.12259
9. Johnson C. Organ Failure and Acute Pancreatitis. In: Forsmark CE, Gardner TB, eds. Prediction and Management of Severe Acute Pancreatitis: Springer New York 2015:15-27.
10. Wang G-J, Gao C-F, Wei D, et al. Acute pancreatitis: Etiology and common pathogenesis. *World Journal of Gastroenterology : WJG* 2009;15(12):1427-30. doi: 10.3748/wjg.15.1427
11. Raraty MT, Connor S, Criddle D, et al. Acute pancreatitis and organ failure: Pathophysiology, natural history, and management strategies. *Current Gastroenterology Reports* 2004;6(2):99-103. doi: 10.1007/s11894-004-0035-0
12. Norman Md J. The Role of Cytokines in the Pathogenesis of Acute Pancreatitis. *The American Journal of Surgery* 1998;175(1):76-83. doi: http://dx.doi.org/10.1016/S0002-9610(97)00240-7
13. Neoptolemos JP. Compartments That Cause the Real Damage in Severe Acute Pancreatitis. *American Journal of Respiratory and Critical Care Medicine* 2003;168(2):141-42. doi: 10.1164/rccm.2305003
14. Johnson C, Abu-Hilal M, Group MotBAPS. Persistent organ failure during the first week as a marker of fatal outcome in acute pancreatitis. *Gut* 2004;53(9):1340-44.
15. Appelros S, Lingren S, Borgstrom A. Short and long-term outcome of severe acute pancreatitis. *Eur J Surg* 2001;167(4):281-86.
16. Zhu A, Shi J, Sun X. Organ failure associated with severe acute pancreatitis. *World Journal of Gastroenterology* 2003;9(11):2570-73.
17. Mole D, Olabi B, Robinson V. Incidence of individual organ dysfunction in fatal acute pancreatitis: analysis of 1024 death records. *HPB* 2009;11(2):166-70.
18. Halonen KI, Leppäniemi AK, Puolakkainen PA, et al. Severe Acute Pancreatitis: Prognostic Factors in 270 Consecutive Patients. *Pancreas* 2000;21(3)
19. Halonen K, Pettilä V, Leppäniemi A, et al. Multiple organ dysfunction associated with severe acute pancreatitis. *Critical Care Medicine* 2002;30(6):1274-79.
20. Banks P, Bollen T, Dervenis C. Classification of acute pancreatitis - 2012: revision of the Atlanta classification and definitions by international consensus. *Gut* 2013;62(1):102-11.
21. Halonen K, Pettila V, Leppaniemi A. Long-term health-related quality of life in survivors of severe acute pancreatitis. *Intensive Care Med* 2003;29(5):782-86.
22. Doepel M, Eriksson J, Halme L, et al. Good long-term results in patients surviving severe acute pancreatitis. *British Journal of Surgery* 1993;80(12):1583-86. doi: 10.1002/bjs.1800801229

For peer review only - http://bmjopen.bmj.com/site/about/guidelines.xhtml

23. Government S. Charter for Safe Havens in Scotland: Handling Unconsented Data from National Health Service Patient Records to Support Research and Statistics. 2015 [Available from: http://www.gov.scot/Publications/2015/11/4783/4.
24. SICSAG [Available from: http://www.sicsag.scot.nhs.uk/index.html accessed January 15 2016.
25. Vital Events Reference Tables 2015 [Available from: http://www.nrscotland.gov.uk/statistics-and-data/statistics/statistics-by-theme/vital-events/vital-events-reference-tables accessed October 30 2015.
26. Society IC. Levels of Critical Care for Adult Patients [Available from: http://www.ics.ac.uk/ICS/Guidelines___Standards/ICS/guidelines-and-standards.aspx?hkey=4ed20a1c-1ff8-46e0-b48e-732f1f4a90e2.
27. Charlson ME, Pompei P, Ales KL, et al. A new method of classifying prognostic comorbidity in longitudinal studies: Development and validation. *Journal of Chronic Diseases* 1987;40(5):373-83. doi: http://dx.doi.org/10.1016/0021-9681(87)90171-8
28. Government S. Scottish Index of Multiple Deprivation 2012: A National Statistics Publication for Scotland. Edinburgh, 2012.
29. Ulvik A, Kvåle R, Wentzel-Larsen T, et al. Multiple organ failure after trauma affects even long-term survival and functional status. *Critical Care* 2007;11(5):R95.
30. Nøjgaard C. Prognosis of acute and chronic pancreatitis: a 30 year follow up of a Danish cohort. *Dan Med Bull* 2010;57(12):B4228.
31. Singer M, De Santis V, Vitale D, et al. Multiorgan failure is an adaptive, endocrine-mediated, metabolic response to overwhelming systemic inflammation. *The Lancet* 2004;364(9433):545-48. doi: http://dx.doi.org/10.1016/S0140-6736(04)16815-3
32. Zilberberg MD, Epstein SK. Acute Lung Injury in the Medical ICU. *American Journal of Respiratory and Critical Care Medicine* 1998;157(4):1159-64. doi: 10.1164/ajrccm.157.4.9704088
33. Williams TA, Dobb GJ, Finn JC, et al. Determinants of long-term survival after intensive care. *Crit Care Med* 2008;36(5):1523-30. doi: 10.1097/CCM.0b013e318170a405
34. Roberts SE, Thorne K, Evans PA, et al. Mortality following acute pancreatitis: social deprivation, hospital size and time of admission: record linkage study. *BMC Gastroenterol* 2014;14:153. doi: 10.1186/1471-230X-14-153
35. van Walraven C, Davis D, Forster AJ, et al. Time-dependent bias was common in survival analyses published in leading clinical journals. *J Clin Epidemiol* 2004;57(7):672-82. doi: 10.1016/j.jclinepi.2003.12.008
36. Statistics on Alcohol 2016 [Available from: https://www.alcoholconcern.org.uk/help-and-advice/statistics-on-alcohol/ accessed January 10 2016.
37. Yancik R, Wesley MN, Ries LAG, et al. Comorbidity and age as predictors of risk for early mortality of male and female colon carcinoma patients. *Cancer* 1998;82(11):2123-34. doi: 10.1002/(SICI)1097-0142(19980601)82:11<2123::AID-CNCR6>3.0.CO;2-W
38. Singh VK, Bollen TL, Wu BU, et al. An Assessment of the Severity of Interstitial Pancreatitis. *Clinical Gastroenterology and Hepatology* 2011;9(12):1098-103. doi: http://dx.doi.org/10.1016/j.cgh.2011.08.026
39. de Beaux AC, Palmer KR, Carter DC. Factors influencing morbidity and mortality in acute pancreatitis; an analysis of 279 cases. *Gut* 1995;37(1):121-26.
40. Farr Institute @ Scotland. About. 2015 [cited 2015 November 14]. Available from: http://www.farrinstitute.org/centre/Scotland/3_About.html accessed November 14 2015.
41. Olmos D, Ang JE, Gomez-Roca C, et al. Pitfalls and limitations of a single-centre, retrospectively derived prognostic score for Phase I oncology trial participants – Reply to Fussenich et al.: A new, simple and objective prognostic score for Phase I cancer patients. *European Journal of Cancer* 2012;48(4):594-96. doi: http://dx.doi.org/10.1016/j.ejca.2011.11.004
42. Altman DG, Bland JM. Generalisation and extrapolation. *BMJ* 1998;317(7155):409-10.
43. Mole D, Boyd J. Resolution of Organ Injury in Acute Pancreatitis - RESORP (RESORP) 2017 [Available from: https://clinicaltrials.gov/ct2/show/NCT03342716 accessed March 14 2018 2018.

For peer review only - http://bmjopen.bmj.com/site/about/guidelines.xhtml

Figure Legends

Figure 1. Study Population Demographics. Visual representation of demographic characteristics for the study cohort (n = 1471 patients) with stacked bar-charts. In all panels, the absolute number of patients per variable category is charted: in red are patients who died in hospital, those who died post-discharge in blue, and those surviving to the end of follow-up in grey. The following attributes of the cohort are depicted sequentially in each respective panel: **A**. Standard Index of Multiple Deprivation (SIMD), **B**. Charlson score, **C.** Gender, **D.** Age (transformed to a categorical variable), and **E.** Number of comorbid conditions contributing to the Charlson score.

Figure 2. Comparison of the Causes of Death between the ccAP cohort and the general population. Bar chart of the causes of death of the post-discharge ccAP cohort (341 deaths – blue colour), and of those of matched controls from the general population of Scotland (381,060 deaths – red colour). The causes of death have been grouped into one of the following categories, according to the ICD-10 code: Cardiovascular/Circulatory, Respiratory, Neoplasia, Digestive/Metabolic, or Other Causes (Suppl. Table 1).

Figure 3. Post-discharge survival of ccAP patients – Patient characteristics. Kaplan-Meier survival plots for post-discharge ccAP patients, grouped by: **A.** Charlson score, **B**. Number of comorbid conditions contributing to each calculated Charlson score, **C**. Gender. The numbers of patients at risk at each time point are displayed. For each plot, in-hospital deaths have been excluded and time zero corresponds to point of discharge. Vertical dashes represent right-censored patients.

Figure 4. Post-discharge survival of ccAP patients – Nature of the critical care admission. Kaplan-Meier survival plots for post-discharge ccAP patients, grouped by: **A.** Duration of critical care admission, **B**. Level of critical care (all ccAP patients), **C.** Level of critical care (post-ccAP patients, excluding in-hospital deaths). The numbers of patients at risk at each time point are displayed for each plot. Vertical dashes represent right-censored patients.

For peer review only - http://bmjopen.bmj.com/site/about/guidelines.xhtml

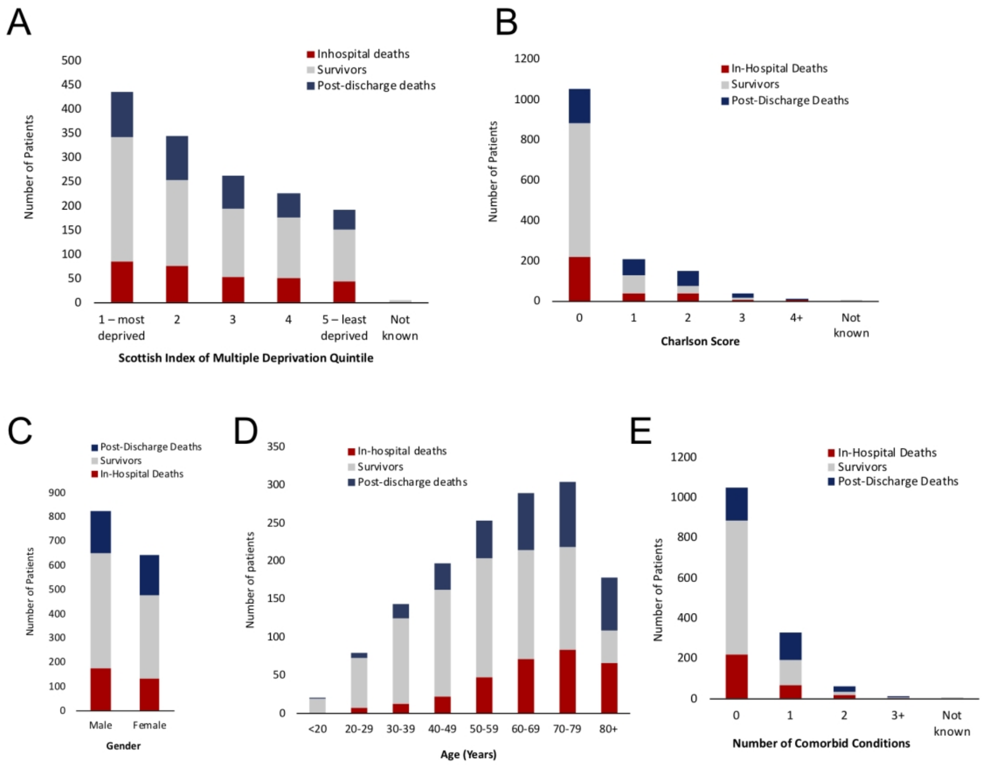

Figure 1. Study Population Demographics. Visual representation of demographic characteristics for the study cohort (n = 1471 patients) with stacked bar-charts. In all panels, the absolute number of patients per variable category is charted: in red are patients who died in hospital, those who died post-discharge in blue, and those surviving to the end of follow-up in grey. The following attributes of the cohort are depicted sequentially in each respective panel: A. Standard Index of Multiple Deprivation (SIMD), B. Charlson score, C. Gender, D. Age (transformed to a categorical variable), and E. Number of comorbid conditions contributing to the Charlson score.

171x135mm (300 x 300 DPI)

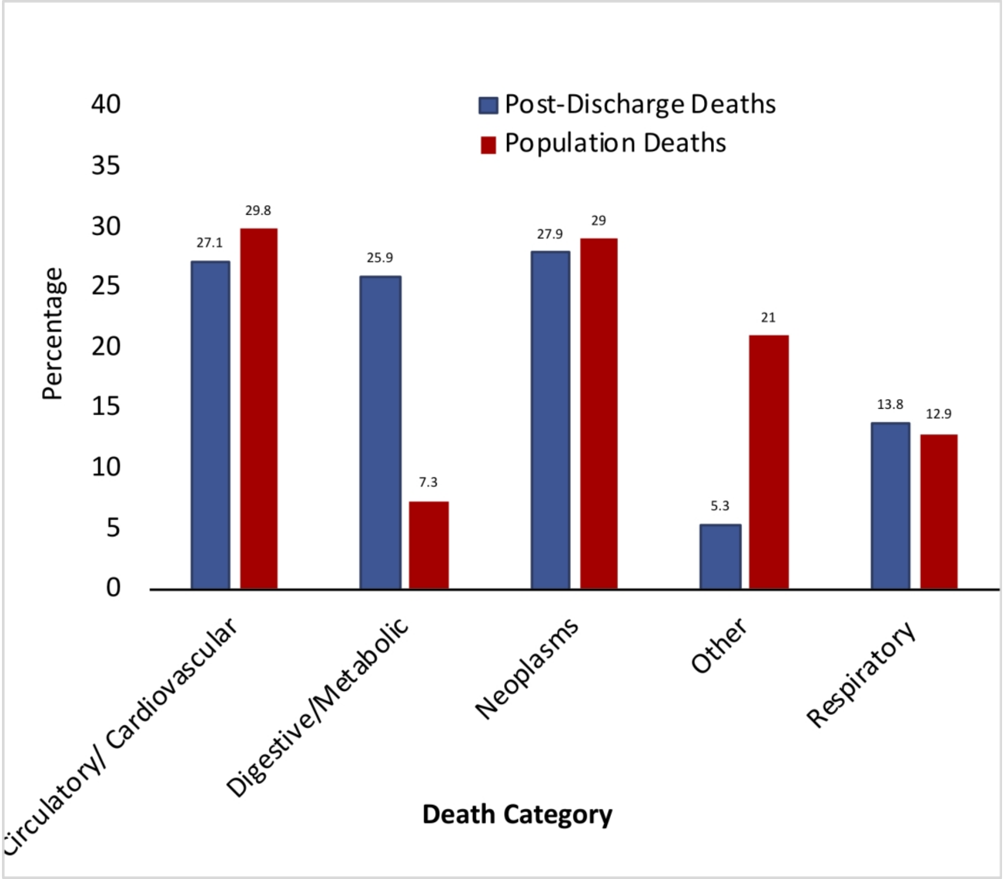

Figure 2. Comparison of the Causes of Death between the ccAP cohort and the general population. Bar chart of the causes of death of the post-discharge ccAP cohort (341 deaths – blue colour), and of those of matched controls from the general population of Scotland (381,060 deaths – red colour). The causes of death have been grouped into one of the following categories, according to the ICD-10 code: Cardiovascular/Circulatory, Respiratory, Neoplasia, Digestive/Metabolic, or Other Causes (Suppl. Table 1).

168x148mm (300 x 300 DPI)

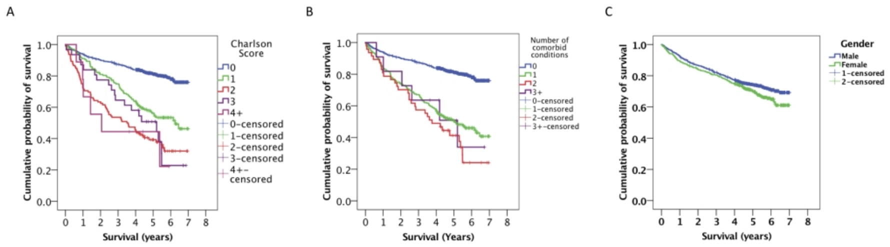

Number at risk/year

	0	1	2	3	4	5	6	7
0	833	782	745	717	685	443	192	0
1	168	152	133	122	104	63	34	0
2	112	80	70	59	47	26	9	0
3	31	26	23	19	17	9	2	0
4+	9	6	4	3	2	1	1	0

Number at risk/year

	0	1	2	3	4	5	6	7
0	833	782	745	717	685	443	192	0
1	261	219	191	172	147	86	42	0
2	47	37	32	26	22	11	2	0
3+	11	9	8	6	5	3	2	0

Number at risk/year

	0	1	2	3	4	5	6	7
Male	650	600	558	523	490	311	136	0
Female	506	452	423	403	374	237	138	0

Figure 3. Post-discharge survival of ccAP patients – Patient characteristics. Kaplan-Meier survival plots for post-discharge ccAP patients, grouped by: A. Charlson score, B. Number of comorbid conditions contributing to each calculated Charlson score, C. Gender. The numbers of patients at risk at each time point are displayed. For each plot, in-hospital deaths have been excluded and time zero corresponds to point of discharge. Vertical dashes represent right-censored patients.

297x209mm (300 x 300 DPI)

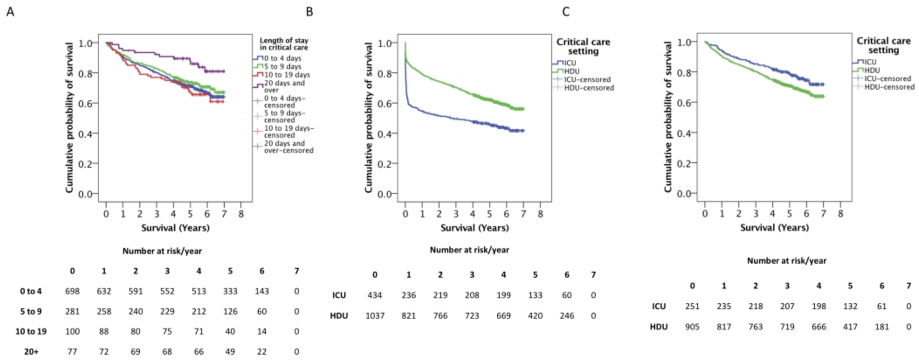

Figure 4. Post-discharge survival of ccAP patients – Nature of the critical care admission. Kaplan-Meier survival plots for post-discharge ccAP patients, grouped by: A. Duration of critical care admission, B. Level of critical care (all ccAP patients), C. Level of critical care (post-ccAP patients, excluding in-hospital deaths). The numbers of patients at risk at each time point are displayed for each plot. Vertical dashes represent right-censored patients.

297x209mm (300 x 300 DPI)

Supplementary data.

Title: Survival and new-onset morbidity after critical care admission for acute pancreatitis: a national electronic healthcare record linkage study.

Authors: Chiara Ventre, Sian Nowell, Cat Graham, Doug Kidd, Christos Skouras and Damian J. Mole

Supplementary material includes:

1 table

11 supplementary figures

On 13 pages

For peer review only

Supplementary Table 1. Categorisation of Causes of Death. The ICD-10 categories for cause of death were allocated to six groups as shown.

Category of death	ICD 10 Code
Circulatory/Cardiovascular system	IX Diseases of the circulatory system
Digestive/Metabolic System	III Diseases of the blood and blood-forming organs and certain disorders involving the immune mechanism<br>*D50-D53 Nutritional anaemias*
	IV Endocrine, nutritional and metabolic diseases
	XI Diseases of the digestive system
	XVIII Symptoms, signs and abnormal clinical and laboratory findings, not elsewhere classified<br>*R10-R19 Symptoms and Signs involving the digestive system and abdomen*
Neoplasms	II Neoplasms
Respiratory System	X Diseases of the Respiratory System
Other	I Certain infectious and parasitic diseases
	III Diseases of the blood and blood-forming organs and certain disorders involving the immune mechanism<br>*Excluding D50-D53*
	V Mental and behavioural disorders
	VI Diseases of the nervous system
	VII Diseases of the eye and adnexa
	VIII Diseases of the ear and mastoid process
	XII Diseases of the skin and subcutaneous tissue
	XIII Diseases of the musculoskeletal system and connective tissue
	XIV Diseases of the genitourinary system
	XV Pregnancy, childbirth and the puerperium
	XVI Certain conditions originating in the perinatal period
	XVII Congenital malformations, deformations and chromosomal abnormalities
	XVIII Symptoms, signs and abnormal clinical and laboratory findings, not elsewhere classified<br>*Excluding R10-R19*
	XIX Injury, poisoning and certain other consequences of external causes
	XX External causes of morbidity and mortality
	XXI Factors influencing health status and contact with health services
	XXII Codes for special purposes

For peer review only

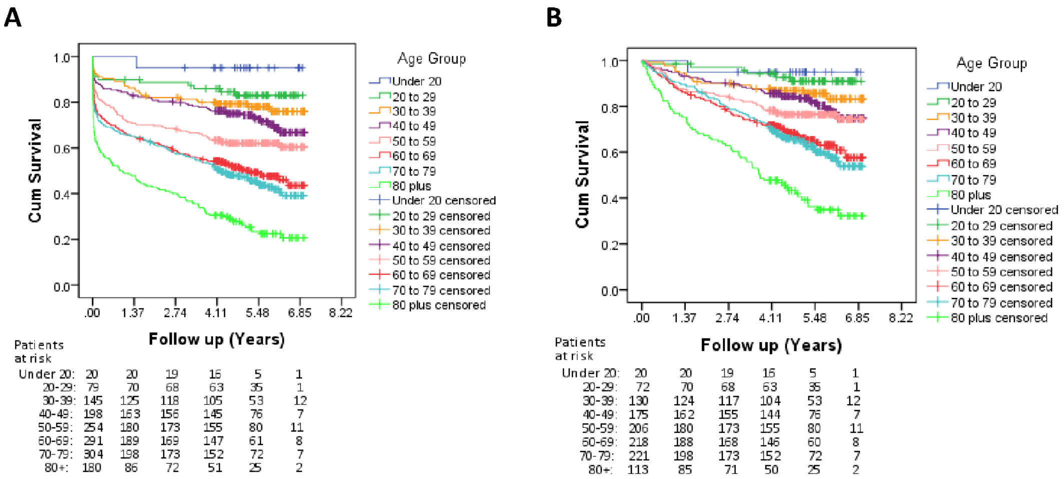

Pairwise Comparisons – including in hospital deaths

	Age group	Under 20		20 to 29		30 to 39		40 to 49		50 to 59		60 to 69		70 to 79		80 plus	
		Chi-Square	Sig.	Chi-Square	Sig.	Chi-Square	Sig.	Chi-Square	Sig.	Chi-Square	Sig.	Chi-Square	Sig.	Chi-Square	Sig.	Chi-Square	Sig.
Log Rank (Mantel-Cox)	Under 20			1.614	.204	2.907	.088	4.199	.040	7.380	.007	11.599	.001	13.092	.000	24.091	.000
	20 to 29	1.614	.204			.954	.329	3.962	.047	11.683	.001	25.914	.000	32.015	.000	66.499	.000
	30 to 39	2.907	.088	.954	.329			1.486	.223	10.898	.001	31.203	.000	40.173	.000	92.223	.000
	40 to 49	4.199	.040	3.962	.047	1.486	.223			5.256	.022	25.194	.000	35.378	.000	92.343	.000
	50 to 59	7.380	.007	11.683	.001	10.898	.001	5.256	.022			8.366	.004	15.589	.000	63.022	.000
	60 to 69	11.599	.001	25.914	.000	31.203	.000	25.194	.000	8.366	.004			1.094	.296	30.182	.000
	70 to 79	13.092	.000	32.015	.000	40.173	.000	35.378	.000	15.589	.000	1.094	.296			21.152	.000
	80 plus	24.091	.000	66.499	.000	92.223	.000	92.343	.000	63.022	.000	30.182	.000	21.152	.000		

Pairwise Comparisons

	Age group	Under 20		20 to 29		30 to 39		40 to 49		50 to 59		60 to 69		70 to 79		80 plus	
		Chi-Square	Sig.	Chi-Square	Sig.	Chi-Square	Sig.	Chi-Square	Sig.	Chi-Square	Sig.	Chi-Square	Sig.	Chi-Square	Sig.	Chi-Square	Sig.
Log Rank (Mantel-Cox)	Under 20			.195	.659	1.212	.271	1.981	.159	3.281	.070	5.997	.014	6.718	.010	15.130	.000
	20 to 29	.195	.659			1.730	.188	4.552	.033	7.764	.005	17.852	.000	21.251	.000	49.740	.000
	30 to 39	1.212	.271	1.730	.188			.986	.321	4.151	.042	16.184	.000	20.474	.000	59.844	.000
	40 to 49	1.981	.159	4.552	.033	.986	.321			1.272	.259	12.308	.000	17.081	.000	60.511	.000
	50 to 59	3.281	.070	7.764	.005	4.151	.042	1.272	.259			5.838	.016	9.458	.002	47.244	.000
	60 to 69	5.997	.014	17.852	.000	16.184	.000	12.308	.000	5.838	.016			.326	.568	22.791	.000
	70 to 79	6.718	.010	21.251	.000	20.474	.000	17.081	.000	9.458	.002	.326	.568			19.713	.000
	80 plus	15.130	.000	49.740	.000	59.844	.000	60.511	.000	47.244	.000	22.791	.000	19.713	.000		

Supplementary Figure 1. Survival by age. Kaplan-Meier plots of the proportion of surviving patients over time in years. Patients were categorised by age. Vertical dashes represent right-censored patients. The number of patients remaining at risk at each time point are presented under the chart. Log-rank pair-wise analyses are presented above the corresponding chart **a.** Analyses including in-hospital deaths. **b.** Analyses excluding in-hospital deaths

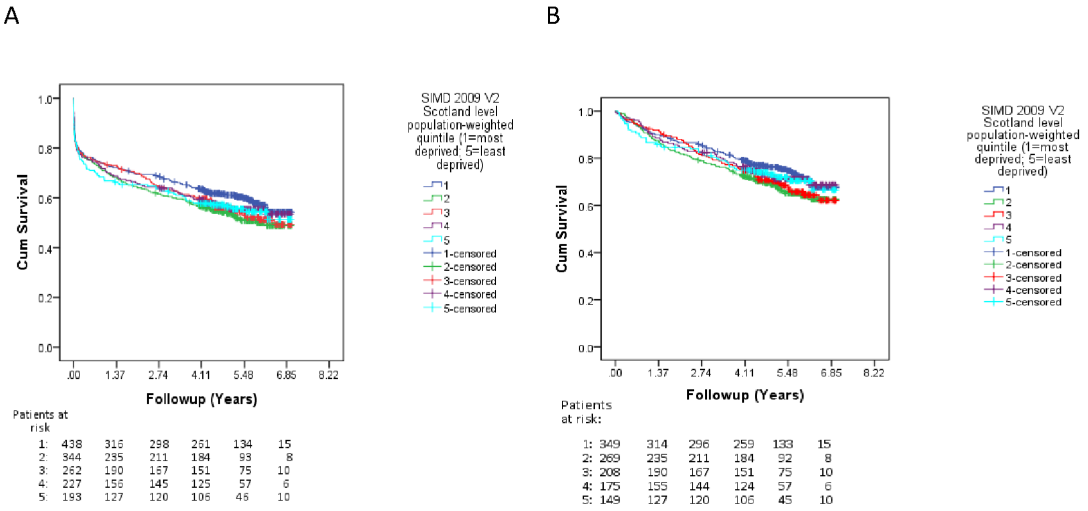

Pairwise Comparisons – including in hospital deaths											
		1		2		3		4		5	
	SIMD 2009 V2 Scotland level population-weighted quintile (1= most deprived; 5=least deprived)	Chi-Square	Sig.	Chi-Square	Sig.	Chi-Square	Sig.	Chi-Square	Sig.	Chi-Square	Sig.
Log Rank (Mantel-Cox)	1			3.634	.057	1.801	.180	.248	.618	.456	.499
	2	3.634	.057			.175	.676	1.207	.272	.695	.405
	3	1.801	.180	.175	.676			.447	.504	.229	.632
	4	.248	.618	1.207	.272	.447	.504			.030	.864
	5	.456	.499	.695	.405	.229	.632	.030	.864		

Pairwise Comparisons – excluding in hospital deaths											
	SIMD 2009 V2 Scotland level population-weighted quintile (1 = most deprived; 5 = least deprived)	1		2		3		4		5	
		Chi-Square	Sig.	Chi-Square	Sig.	Chi-Square	Sig.	Chi-Square	Sig.	Chi-Square	Sig.
Log Rank (Mantel-Cox)	1			3.473	.062	1.339	.247	.602	.438	1.130	.288
	2	3.473	.062			.293	.588	.643	.423	.205	.650
	3	1.339	.247	.293	.588			.075	.784	.000	.993
	4	.602	.438	.643	.423	.075	.784			.075	.785
	5	1.130	.288	.205	.650	.000	.993	.075	.785		

Supplementary Figure 2. Survival by Scottish Index of Multiple Deprivation (SIMD) quintile. Kaplan-Meier plots of the proportion of surviving patients over time in years. Patients were categorised by SIMD quintile. Vertical dashes represent right-censored patients. The number of patients remaining at risk at each time point are presented under the chart. Log-rank pair-wise analyses are presented above the corresponding chart **a.** Analyses including in-hospital deaths. **b.** Analyses excluding in-hospital deaths

A

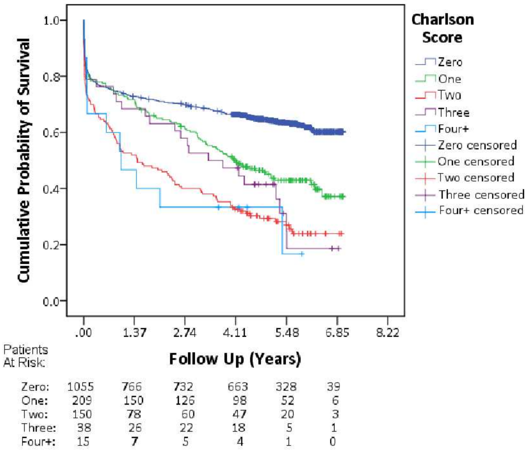

B

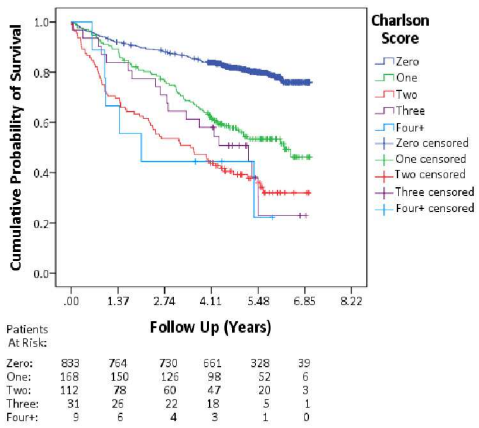

Pairwise Comparisons – including in-hospital deaths

	Charlson score	0		1		2		3		4+	
		Chi-Square	Sig.	Chi-Square	Sig.	Chi-Square	Sig.	Chi-Square	Sig.	Chi-Square	Sig.
Log Rank (Mantel-Cox)	0			22.485	.000	74.261	.000	12.801	.000	9.426	.002
	1	22.485	.000			13.189	.000	1.166	.280	3.167	.075
	2	74.261	.000	13.189	.000			1.147	.284	.038	.846
	3	12.801	.000	1.166	.280	1.147	.284			.782	.376
	4+	9.426	.002	3.167	.075	.038	.846	.782	.376		

Pairwise Comparisons – excluding in hospital deaths

		0		1		2		3		4+	
	Charlson score	Chi-Square	Sig.	Chi-Square	Sig.	Chi-Square	Sig.	Chi-Square	Sig.	Chi-Square	Sig.
Log Rank (Mantel-Cox)	0			52.398	.000	135.915	.000	35.007	.000	19.238	.000
	1	52.398	.000			12.621	.000	2.097	.148	3.383	.066
	2	135.915	.000	12.621	.000			.495	.482	.067	.796
	3	35.007	.000	2.097	.148	.495	.482			.441	.507
	4+	19.238	.000	3.383	.066	.067	.796	.441	.507		

Supplementary Figure 3. Survival by Charlson Score. Kaplan-Meier plots of the proportion of surviving patients over time in years. Patients were categorised by Charlson score. Vertical dashes represent right-censored patients. The number of patients remaining at risk at each time point are presented under the chart. Log-rank pair-wise analyses are presented above the corresponding chart **a.** Analyses including in-hospital deaths. **b.** Analyses excluding in-hospital deaths

A

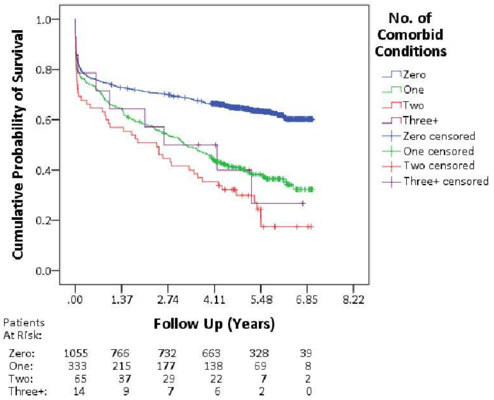

B

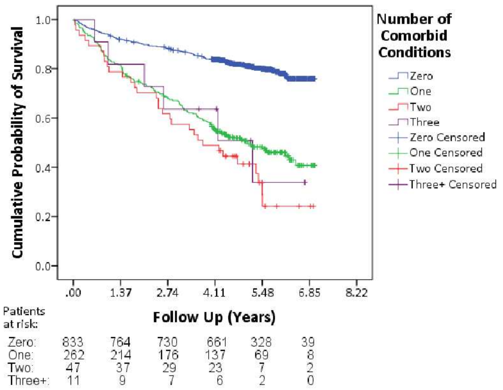

Pairwise Comparisons – including in hospital deaths									
		0		1		2		3+	
	Charlson No. of Conditios	Chi-Square	Sig.	Chi-Square	Sig.	Chi-Square	Sig.	Chi-Square	Sig.
Log Rank (Mantel-Cox)	0			57.146	.000	40.233	.000	3.749	.053
	1	57.146	.000			4.247	.039	.002	.968
	2	40.233	.000	4.247	.039			.607	.436
	3+	3.749	.053	.002	.968	.607	.436		

Pairwise Comparisons – excluding in hospital deaths									
	Charlson No. of Conditions	.00		1		2		3+	
		Chi-Square	Sig.	Chi-Square	Sig.	Chi-Square	Sig.	Chi-Square	Sig.
Log Rank (Mantel-Cox)	.00			113.399	.000	69.509	.000	9.289	.002
	1	113.399	.000			2.654	.103	.003	.954
	2	69.509	.000	2.654	.103			.324	.569
	3+	9.289	.002	.003	.954	.324	.569		

Supplementary Figure 4. Survival by number of conditions contributing to the Charlson Score. Kaplan-Meier plots of the proportion of surviving patients over time in years. Patients were categorised by the number of conditions contributing to their Charlson score. Vertical dashes represent right-censored patients. The number of patients remaining at risk at each time point are presented under the chart. Log-rank pair-wise analyses are presented above the corresponding chart **a.** Analyses including in-hospital deaths. **b.** Analyses excluding in-hospital deaths

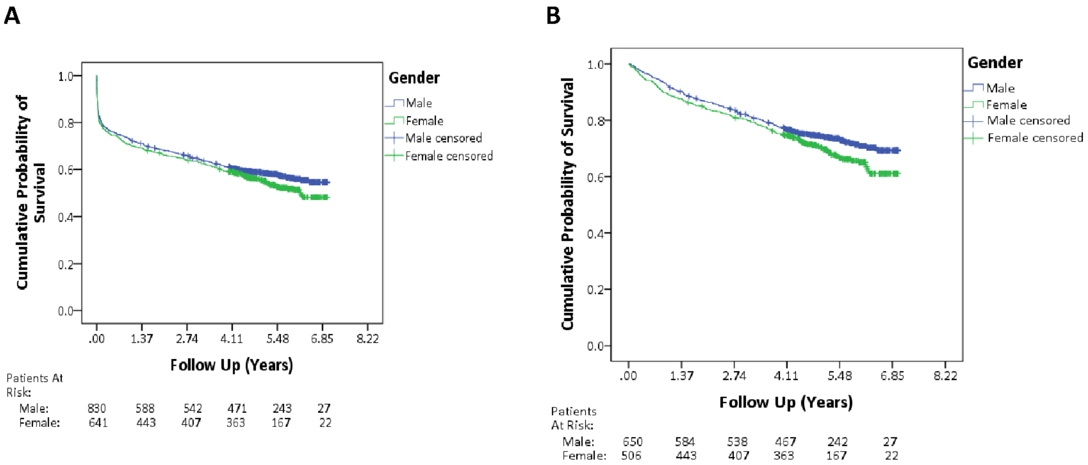

Supplementary Figure 5. Gender. Kaplan-Meier plots of the proportion of surviving patients over time in years. Patients were categorised by gender. Vertical dashes represent right-censored patients. The number of patients remaining at risk at each time point are presented under the chart. Log-rank analyses are presented to the right of the corresponding chart **a.** Analyses including in-hospital deaths (Log Rank test, P = 0.134). **b.** Analyses excluding in-hospital deaths (Log Rank test, P = 0.049)

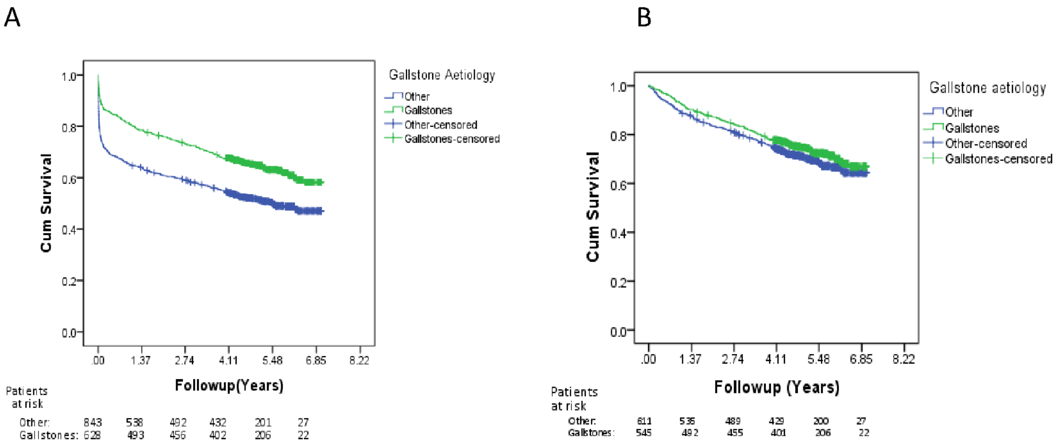

Supplementary Figure 6. Survival by aetiology of pancreatitis. Kaplan-Meier plots of the proportion of surviving patients over time in years. Patients were categorised by aetiology – gallstones or other causes. Vertical dashes represent right-censored patients. The number of patients remaining at risk at each time point are presented under the chart. Log-rank analyses are presented to the right of the corresponding chart **a.** Analyses including in-hospital deaths. (Log Rank test, Gallstones vs Other, P<0.001) **b.** Analyses excluding in-hospital deaths (Log Rank test, Gallstones vs Other, P=0.139)

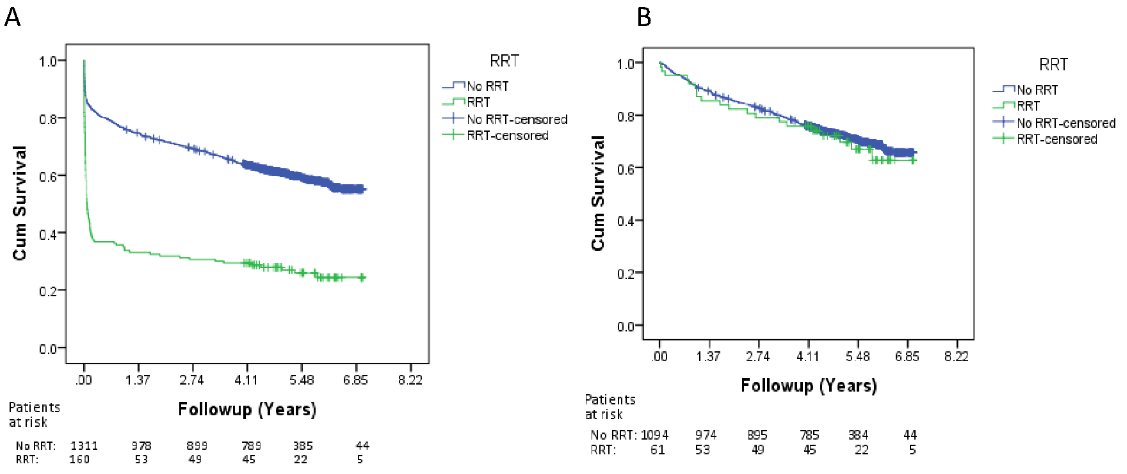

Supplementary Figure 7. Survival comparing use of renal replacement therapy (RRT). Kaplan-Meier plots of the proportion of surviving patients over time in years. Patients were categorised by whether they required RRT during the index admission. Vertical dashes represent right-censored patients. The number of patients remaining at risk at each time point are presented under the chart. Log-rank analyses are presented to the right of the corresponding chart **a.** Analyses including in-hospital deaths (Log Rank test RRT vs no RRT, P <0.001). **b.** Analyses excluding in-hospital deaths (Log Rank test RRT vs no RRT, P = 0.634).

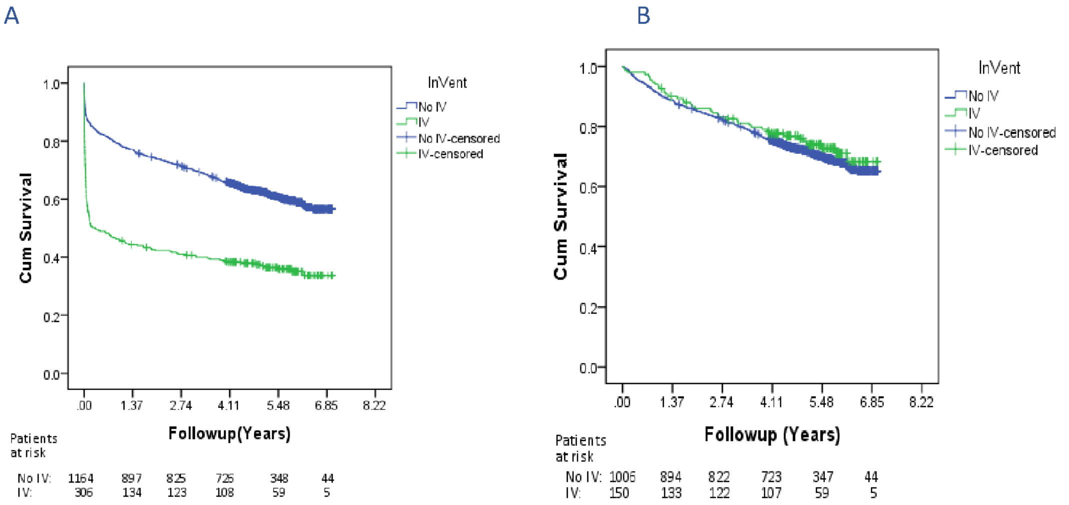

Supplementary Figure 8. Survival according to use of invasive ventilation. Kaplan-Meier plots of the proportion of surviving patients over time in years. Patients were categorised by whether they required invasive ventilation during the index admission. Vertical dashes represent right-censored patients. The number of patients remaining at risk at each time point are presented under the chart. Log-rank analyses are presented to the right of the corresponding chart **a.** Analysis including in-hospital deaths (Log Rank test inv. vent vs no inv. vent, P < 0.001). **b.** Analysis excluding in-hospital deaths (Log Rank test inv. vent vs no inv. vent, P = 0.428).

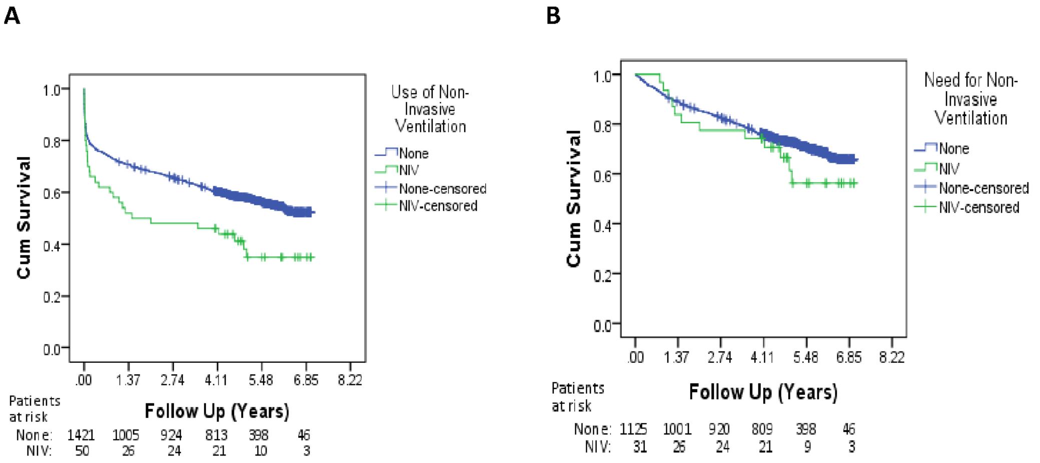

Supplementary Figure 9. Use of non-invasive ventilation (NIV). Kaplan-Meier plots of the proportion of surviving patients over time in years. Patients were categorised by whether or not they required NIV during the index admission. Vertical dashes represent right-censored patients. The number of patients remaining at risk at each time point are presented under the chart. Log-rank analyses are presented to the right of the corresponding chart **a.** Analyses including in-hospital deaths (Log Rank test NIV vs no NIV, P = 0.008). **b.** Analysis excluding in-hospital deaths (Log Rank test NIV vs no NIV, P = 0.301)

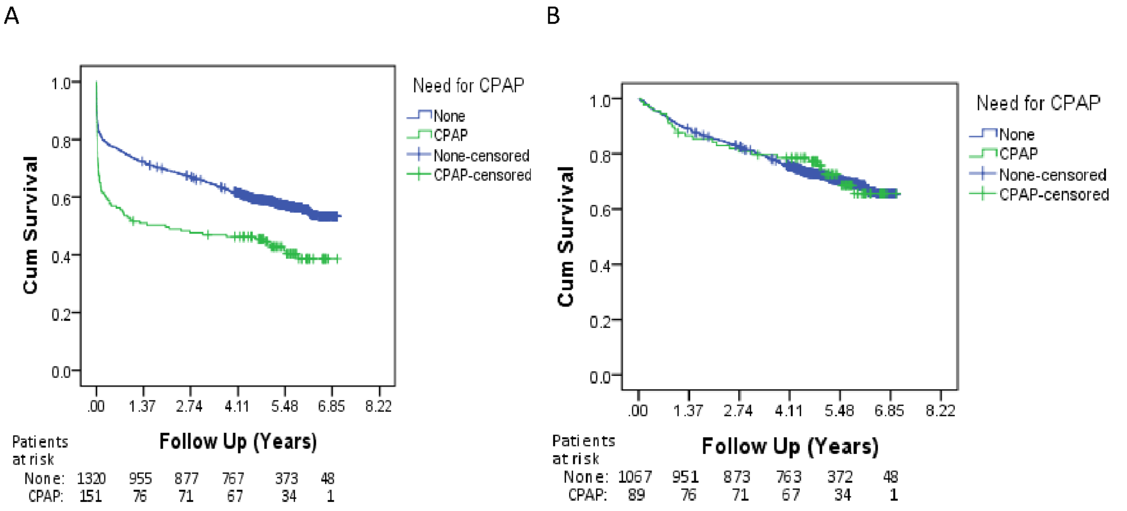

Supplementary Figure 10. Survival according to use of continuous positive airway pressure (CPAP). Kaplan-Meier plots of the proportion of surviving patients over time in years. Patients were categorised by whether they required CPAP during the index admission. Vertical dashes represent right-censored patients. The number of patients remaining at risk at each time point are presented under the chart. Log-rank analyses are presented to the right of the corresponding chart **a.** Analyses including in-hospital deaths (Log Rank test CPAP vs no CPAP, P < 0.001). **b.** Analysis excluding in-hospital deaths (Log Rank test CPAP vs no CPAP, P = 0.930)

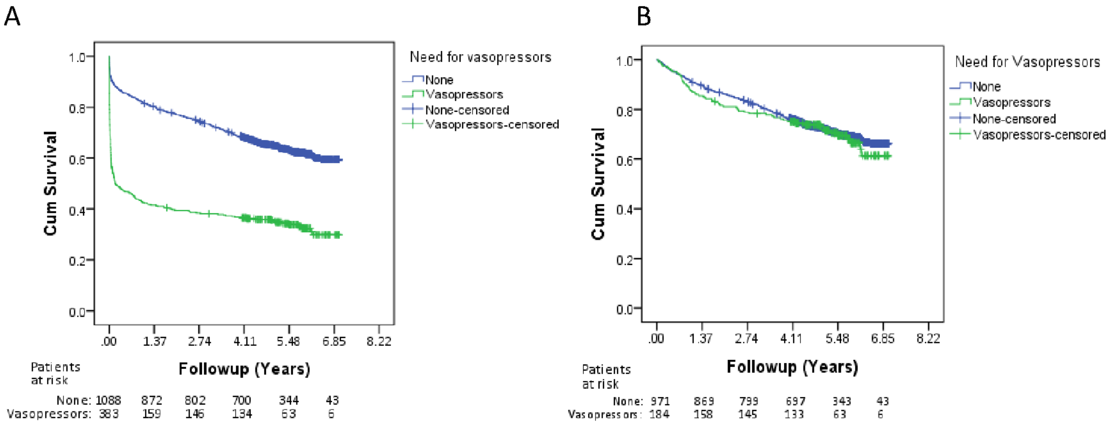

Supplementary Figure 11. Survival according to use of vasopressors. Kaplan-Meier plots of the proportion of surviving patients over time in years. Patients were categorised by whether they required vasopressors during the index admission. Vertical dashes represent right-censored patients. The number of patients remaining at risk at each time point are presented under the chart. Log-rank analyses are presented above the corresponding chart **a.** Analyses including in-hospital deaths (Log Rank test Vasopressors vs no Vasopressors, P < 0.001). **b.** Analysis excluding in-hospital deaths (Log Rank test Vasopressors vs no Vasopressors, P = 0.579)

Reporting checklist for cohort study.

Based on the STROBE cohort guidelines.

Instructions to authors

Complete this checklist by entering the page numbers from your manuscript where readers will find each of the items listed below.

Your article may not currently address all the items on the checklist. Please modify your text to include the missing information. If you are certain that an item does not apply, please write "n/a" and provide a short explanation.

Upload your completed checklist as an extra file when you submit to a journal.

In your methods section, say that you used the STROBE cohort reporting guidelines, and cite them as:

von Elm E, Altman DG, Egger M, Pocock SJ, Gotzsche PC, Vandenbroucke JP. The Strengthening the Reporting of Observational Studies in Epidemiology (STROBE) Statement: guidelines for reporting observational studies.

		Reporting Item	Page Number
Title	#1a	Indicate the study's design with a commonly used term in the title or the abstract	1
Abstract	#1b	Provide in the abstract an informative and balanced summary of what was done and what was found	2
Background / rationale	#2	Explain the scientific background and rationale for the investigation being reported	4
Objectives	#3	State specific objectives, including any prespecified hypotheses	4
Study design	#4	Present key elements of study design early in the paper	5
Setting	#5	Describe the setting, locations, and relevant dates, including periods of recruitment, exposure, follow-up, and data collection	5
Eligibility criteria	#6a	Give the eligibility criteria, and the sources and methods of selection of participants. Describe methods of follow-up.	5

For peer review only

	#6b	For matched studies, give matching criteria and number of exposed and unexposed	6
Variables	#7	Clearly define all outcomes, exposures, predictors, potential confounders, and effect modifiers. Give diagnostic criteria, if applicable	5
Data sources / measurement	#8	For each variable of interest give sources of data and details of methods of assessment (measurement). Describe comparability of assessment methods if there is more than one group. Give information separately for for exposed and unexposed groups if applicable.	5
Bias	#9	Describe any efforts to address potential sources of bias	5
Study size	#10	Explain how the study size was arrived at	5
Quantitative variables	#11	Explain how quantitative variables were handled in the analyses. If applicable, describe which groupings were chosen, and why	6
Statistical methods	#12a	Describe all statistical methods, including those used to control for confounding	6
	#12b	Describe any methods used to examine subgroups and interactions	6
	#12c	Explain how missing data were addressed	5
	#12d	If applicable, explain how loss to follow-up was addressed	5
	#12e	Describe any sensitivity analyses	n/a
Participants	#13a	Report numbers of individuals at each stage of study—eg numbers potentially eligible, examined for eligibility, confirmed eligible, included in the study, completing follow-up, and analysed. Give information separately for for exposed and unexposed groups if applicable.	7
	#13b	Give reasons for non-participation at each stage	7
	#13c	Consider use of a flow diagram	7
Descriptive data	#14a	Give characteristics of study participants (eg demographic, clinical, social) and information on exposures and potential	18

For peer review only

		confounders. Give information separately for exposed and unexposed groups if applicable.	
	#14b	Indicate number of participants with missing data for each variable of interest	7
	#14c	Summarise follow-up time (eg, average and total amount)	7
Outcome data	#15	Report numbers of outcome events or summary measures over time. Give information separately for exposed and unexposed groups if applicable.	7
Main results	#16a	Give unadjusted estimates and, if applicable, confounder-adjusted estimates and their precision (eg, 95% confidence interval). Make clear which confounders were adjusted for and why they were included	19
	#16b	Report category boundaries when continuous variables were categorized	n/a
	#16c	If relevant, consider translating estimates of relative risk into absolute risk for a meaningful time period	8
Other analyses	#17	Report other analyses done—e.g., analyses of subgroups and interactions, and sensitivity analyses	8
Key results	#18	Summarise key results with reference to study objectives	9
Limitations	#19	Discuss limitations of the study, taking into account sources of potential bias or imprecision. Discuss both direction and magnitude of any potential bias.	10
Interpretation	#20	Give a cautious overall interpretation considering objectives, limitations, multiplicity of analyses, results from similar studies, and other relevant evidence.	11
Generalisability	#21	Discuss the generalisability (external validity) of the study results	10
Funding	#22	Give the source of funding and the role of the funders for the present study and, if applicable, for the original study on which the present article is based	1

The STROBE checklist is distributed under the terms of the Creative Commons Attribution License CC-BY. This checklist was completed on 18. April 2018 using http://www.goodreports.org/, a tool made by the EQUATOR Network in collaboration with Penelope.ai

For peer review only
